# Phylodynamics unveils invading and diffusing patterns of dengue virus serotype-1 in Guangdong, China from 1990 to 2019 under a global genotyping framework

**DOI:** 10.1186/s40249-024-01211-6

**Published:** 2024-06-11

**Authors:** Lingzhai Zhao, Xiang Guo, Liqiang Li, Qinlong Jing, Jinmin Ma, Tian Xie, Dechun Lin, Li Li, Qingqing Yin, Yuji Wang, Xiaoqing Zhang, Ziyao Li, Xiaohua Liu, Tian Hu, Minling Hu, Wenwen Ren, Jun Li, Jie Peng, Lei Yu, Zhiqiang Peng, Wenxin Hong, Xingyu Leng, Lei Luo, Jone Jama Kpanda Ngobeh, Xiaoping Tang, Rangke Wu, Wei Zhao, Benyun Shi, Jiming Liu, Zhicong Yang, Xiao-Guang Chen, Xiaohong Zhou, Fuchun Zhang

**Affiliations:** 1 Institute of Infectious Diseases, Guangzhou Eighth People’s Hospital, Guangzhou Medical University, Guangzhou, 510440 Guangdong China; 2 Department of Pathogen Biology, School of Public Health, Institute of Tropical Medicine, Southern Medical University; Guangdong Provincial Key Laboratory of Tropical Disease Research; Key Laboratory of Prevention and Control for Emerging Infectious Diseases of Guangdong Higher Institutes; Key Laboratory of Infectious Diseases Research in South China of Ministry of Education, Guangzhou, 510515 China; 3 Department of Clinical Laboratory, The Third People’s Hospital of Shenzhen, Southern University of Science and Technology, National Clinical Research Center for Infectious Diseases, Guangdong Provincial Clinical Research Center for Infectious Diseases (Tuberculosis), Shenzhen Clinical Research Center for Tuberculosis, Shenzhen, China; 4 Guangzhou Center for Disease Control and Prevention, Guangzhou, 510440 China; 5 BGI-Shenzhen, Shenzhen, 518083 China; 6 Department of Biostatistics, School of Public Health, State Key Laboratory of Organ Failure Research, Guangdong Provincial Key Laboratory of Tropical Disease Research, Southern Medical University, Guangzhou, 510515 China; 7 Guangdong Provincial Key Laboratory of Research On Emergency in TCM, Guangdong Provincial Hospital of Traditional Chinese Medicine, Guangzhou, 510120 China; 8 Department of Infectious Diseases, Nanfang Hospital, Southern Medical University, Guangzhou, 510515 China; 9 Guangdong Provincial Center for Disease Control and Prevention, Guangzhou, 511430 China; 10 The School of Foreign Studies, Southern Medical University, Guangzhou, 510515 China; 11 BSL-3 Laboratory(Guangdong), School of Public Health, Southern Medical University, Guangzhou, 510515 China; 12 College of Computer and Information Engineering, Nanjing Tech University, Nanjing, 211816 China; 13 Department of Computer Science, Hong Kong Baptist University, Hong Kong, 999077 China; 14 Guangzhou Medical Research Institute of Infectious Diseases, Infectious Disease Center, Guangzhou Eighth People’s Hospital, Guangzhou Medical University, Guangzhou, 510440 China

**Keywords:** Dengue serotype-1, Molecular epidemiology, Genetic population structure, Phylogeography, Phylodynamics, Epidemic periodicity, Global genotyping framework, China

## Abstract

**Background:**

The strong invasiveness and rapid expansion of dengue virus (DENV) pose a great challenge to global public health. However, dengue epidemic patterns and mechanisms at a genetic scale, particularly in term of cross-border transmissions, remain poorly understood. Importation is considered as the primary driver of dengue outbreaks in China, and since 1990 a frequent occurrence of large outbreaks has been triggered by the imported cases and subsequently spread to the western and northern parts of China. Therefore, this study aims to systematically reveal the invasion and diffusion patterns of DENV-1 in Guangdong, China from 1990 to 2019.

**Methods:**

These analyses were performed on 179 newly assembled genomes from indigenous dengue cases in Guangdong, China and 5152 E gene complete sequences recorded in Chinese mainland. The genetic population structure and epidemic patterns of DENV-1 circulating in Chinese mainland were characterized by phylogenetics, phylogeography, phylodynamics based on DENV-1 E-gene-based globally unified genotyping framework.

**Results:**

Multiple serotypes of DENV were co-circulating in Chinese mainland, particularly in Guangdong and Yunnan provinces. A total of 189 transmission clusters in 38 clades belonging to 22 subgenotypes of genotype I, IV and V of DENV-1 were identified, with 7 Clades of Concern (COCs) responsible for the large outbreaks since 1990. The epidemic periodicity was inferred from the data to be approximately 3 years. Dengue transmission events mainly occurred from Great Mekong Subregion-China (GMS-China), Southeast Asia (SEA), South Asia Subcontinent (SASC), and Oceania (OCE) to coastal and land border cities respectively in southeastern and southwestern China. Specially, Guangzhou was found to be the most dominant receipting hub, where DENV-1 diffused to other cities within the province and even other parts of the country. Genome phylogeny combined with epidemiological investigation demonstrated a clear local consecutive transmission process of a 5C1 transmission cluster (5C1-CN4) of DENV-1 in Guangzhou from 2013 to 2015, while the two provinces of Guangdong and Yunnan played key roles in ongoing transition of dengue epidemic patterns. In contextualizing within Invasion Biology theories, we have proposed a derived three-stage model encompassing the stages of invasion, colonization, and dissemination, which is supposed to enhance our understanding of dengue spreading patterns.

**Conclusions:**

This study demonstrates the invasion and diffusion process of DENV-1 in Chinese mainland within a global genotyping framework, characterizing the genetic diversities of viral populations, multiple sources of importation, and periodic dynamics of the epidemic. These findings highlight the potential ongoing transition trends from epidemic to endemic status offering a valuable insight into early warning, prevention and control of rapid spreading of dengue both in China and worldwide.

**Graphical Abstract:**

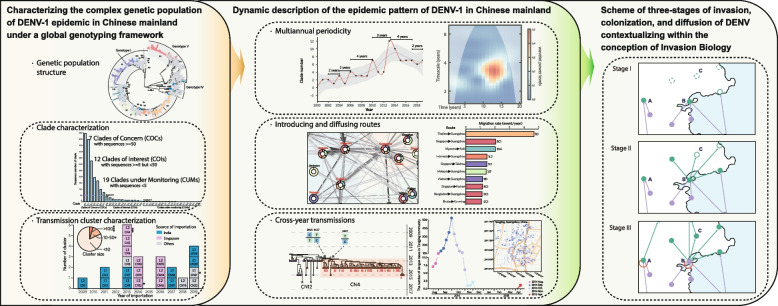

**Supplementary Information:**

The online version contains supplementary material available at 10.1186/s40249-024-01211-6.

## Background

Dengue, an acute febrile disease transmitted by *Aedes aegypti* or *Ae. albopictus* mosquitoes, is caused by dengue virus (DENV) infection and poses a great challenge to global public health. Annually, the newly infected cases are estimated to amount to a number of 390 million, putting the health of 2.5 to 4 billion people at risk worldwide [1]. The escalating disease burden of dengue is broadly attributed to such intricate factors as unplanned urbanization, climatic warming, international trade, and traffic integration, all of which contribute to its irreversible trend [2]. The rapid spreading of dengue has resulted in a series of invasive outbreaks in temperate regions, such as Italy [3] and northern China [4] in recent years. In China, for example, since its re-emergence in 1978 in Foshan of Guangdong Province, the disease has developed to become an annual epidemic and spread even from the southeast coastal areas to the northern and western parts of the country [5]. Meanwhile, DENV has been identified and documented with four dengue serotypes in the country during this period, among which DENV-1 has emerged as the predominant serotype in recent years [6].

The global genotyping framework of DENV-1, based on the E-gene, established in our previous study demonstrates a robust geographic constraint and reveals stratified spatial-genetic epidemic pairs at the Continent-Genotype, Region-Subgenotype, and Nation-Clade levels [7]. Additionally, it highlights the increasing cross-transmission and rapid global spread of dengue driven by traditional endemic sourcing, emerging epidemic diffusion, and potentially hidden epidemics [7]. Herein, the emerging dengue epidemic in China serves as an example, illustrating a scenario that previous dengue outbreaks in Chinese mainland predominantly resulted from local transmissions triggered by imported cases, particularly from South and Southeast Asia, and these local transmission were contingent upon the suitability of the local bio-socioecological environment [8]. Although the invasion and diffusion characteristics of DENV have been assessed based on epidemiology, biostatistics, and virus phylogeny [8–10], our understanding of DENV epidemic patterns and mechanisms at a genetic level, particularly in terms of cross-border transmissions, remains limited. The urgency of curbing DENV expansion calls for accurate tracking of the transmission chains and the establishment of an outbreak mapping based on recent advances in virus genome sequencing as well as phylogenetics under a unified genotyping framework [11].

Therefore, in the present study, we conducted whole genome sequencing of DENV from viral RNA extracted from blood samples collected from 179 indigenous dengue cases during the epidemics in Guangdong Province of China from 2006 to 2019. We systematically revealed that the complex genetics population structure of DENV-1 epidemic in Chinese mainland based on the unified global genotyping scheme of DENV-1 established in our previous work [7]. By contextualizing this study within Invasion Biology theories, specially taking dengue epidemics in Guangdong as an example, we aim to provide novel insights into the process of DENV introduction and diffusion in emerging epidemic areas, thereby contributing to the development of a stratified coordinated strategy for blocking rapid spread of dengue.

## Methods

### Epidemiological data collection

In this study, the dengue surveillance data during the period of 1990‒2019 were obtained from archived records (China Notifiable Disease Surveillance System) of the Guangdong Provincial Center and the Guangzhou Center for Diseases Control and Prevention (Additional file 1: Table S1) because dengue was recognized as a national notifiable infectious disease in Chinese mainland since 1989. Dengue cases are diagnosed according to the China National Diagnostic criteria for dengue fever (WS216), enacted by the National Health Commission of China referring to WHO diagnostics criteria in consideration of China conditions. In brief, dengue cases were categorized as epidemiological history investigation, clinical diagnosed and laboratory-confirmed cases. Clinically diagnosed cases were identified by experienced local physician according to the clinical manifestations as acute onset of rash, fever, itching, anorexia, or arthralgia. Laboratory-confirmed cases were determined with following lab test results: (1) decreased white blood cell count and/or decreased platelet count; (2) positive result of specific IgM antibody; (3) positive result of NS1 antigen within 5 days of onset; (4) The convalescent serum of dengue virus shows a specific increase in IgG antibody titer of at least four times or more compared to the acute phase, or converts from negative to positive; (5) positive on virus isolation test; (6) positive on a reverse transcription-polymerase chain reaction (RT-PCR) test or real-time reverse transcription-polymerase chain reaction (qRT-PCR) test.

### Sample collection and ethics statement

The sera samples for DENV-1 genome sequencing were conveniently collected from 179 indigenous dengue cases hospitalized at the Guangzhou Eighth People’s Hospital between 2006 and 2019 (Additional file 2: Table S2). All the patients were tested to be DENV-1 positive by RT-qPCR using dengue serotype-specific RT-qPCR Kit (DAAN Ltd, Guangzhou, China). The ethical committee of the Guangzhou Eighth People’s Hospital of Guangzhou Medical University approved the study (No. 20160264). The study conformed to the principles of the Declaration of Helsinki. All the adult subjects and parents or guardians in case of minors provided written informed consents.

### Viral RNA extraction, library preparation, sequencing and genome assembly

Total RNAs were extracted from 200 μl of DENV-1 positive serum using the Qiagen RNeasy kit (Qiagen, Dusseldorf, Germany). RNA concentration and quality were measured using the Agilent 2100 Bioanalyzer (Agilent,Santa Clara, USA). Approximately 2 μg of total RNA was fragmented with Covaris E210 (Covaris, Woburn, USA). Using these short fragments as templates, random hexamer primers were used to synthesize the first-strand cDNA. The second-strand cDNA was synthesized in the reaction buffer containing dNTPs, RNase H, and DNA polymerase I. Short double-stranded cDNA fragments were purified with a QIA Quick PCR extraction kit (Qiagen, Dusseldorf, Germany) and end-repaired with the addition of 3’-A overhangs.

In brief, we used IonTorrent and Illumina sequencing for genome acquisition. The library and sequencing progress of IonTorrent is as follow: the short DNA fragments were ligated to Ion Torrent-compatible barcoded adapters. DNA fragments of a selected size (200 bp) were gel-purified and amplified. AMPure beads (Beckman Coulter, Pasadena, USA) were used to purify the resulting library, and an Agilent 2100 BioAnalyzer (Agilent Technologies, Santa Clara, USA) and an Agilent BioAnalyzer DNA High-Sensitivity LabChip (Agilent Technologies, Santa Clara, USA) were used to determine the concentration and size of the library. The libraries were pooled in equal volumes and emulsion PCR-amplified on Ion Sphere Particles (ISPs) using the Ion One Touch instrument (Thermo Fisher Scientific, Waltham, USA). The template-positive ISPs were enriched on the Ion One Touch ES instrument (Thermo Fisher Scientific, Waltham, USA) using Ion PI™Template OT2 200 Kit v3. Ion PI™Chips (Thermo Fisher Scientific, Waltham, USA) were used for sequencing on Ion Torrent Proton platform (Thermo Fisher Scientific, Waltham, USA) by the Beijing Genomics Institute. Ion PI™Sequencing 200 Kit v3 was used for sequencing reactions upon the manufacturer’s instructions. The raw reads generated from the Ion Torrent Proton instrument were sorted by barcode, with each sample producing an average of 28 million reads. Subsequently, these reads from the Proton sequencing platform were mapped to the human genome (hg19) using TMAP 3.4.1 (https://github.com/iontorrent/TMAP) for filtering out the human genome related reads. Unmapped reads were mapped to the dengue virus reference sequence downloaded from the National Center of Biotechnology Information (NCBI) through TMAP 3.4.1 (https://github.com/iontorrent/TMA). Finally, the raw reads were assembled into contigs using IDBA-trans 1.1.1 (http://www.cs.hku.hk/~alse/idba_tran).

The library and sequencing progress of Illumina is as follow: the first-strand cDNA was synthesized by reverse transcription using random hexamers tagged with a known sequence. Double-stranded cDNA was developed by Klenow polymerase, and then the product was subsequently used as the template for PCR amplification. The amplified DNA was purified and used for high-throughput sequencing by Hiseq 2500 (Illumina San Diego, USA) platform (paired-end, 2 × 125 bp). DENV genomes were assembled from the clean reads by CLC Genomics Workbench v11.0 (QIAGEN, USA).

### Phylogenetic identification of DENV-1 indigenous strains circulated in Chinese mainland using the E-gene-based global genotyping framework

Based on the unified global genotyping framework of DENV-1 [7], a total of 5152 E complete sequences of DENV-1 including 179 newly assembled genomes (Additional file 2: Table S2) in this study and previous studies reported from 1978 to 2019 in Chinese mainland were utilized for phylogenic genotyping by maximum likelihood algorithm using a GTR + G substitution model with 10,000 usltrafast boostrap with IQTREE [12]. Then, the included sequences were distributed on the phylogenic branches of DENV-1 global framework proposed in our previous study [7], thereby their subgenotypes and clades were assigned.

### Time-resolved phylogenetic tree reconstruction

The newly assembled genomes from indigenous dengue cases were integrated with reported genotypes of E gene sequences and whole genomes to create novel datasets. Meanwhile, the dataset was deduplicated by removing redundant sequences such as those with same genetic information of same spatiotemporal distribution or same strains. The datasets for Clades 1E1, 1H4, 1J7, 1K1, 1L1, 1L2, and 5C1 consisted of E gene and whole genome data comprising respectively: 251, 98, 126, 56, 178, 143, and 206 (E genes); 90, 27,63, 42, 79,39, and 216(Genomes). Subsequently, separate analyses were conducted on the E genes and genomes of these clades. The correlation between root-to-tip genetic divergence and sequence sampling dates was first estimated using TempEst [13]. Time-resolved phylogenic trees were inferred by BEAST v1.10.4 using a GTR + I + G nucleotide substitution model and an uncorrelated lognormal relaxed molecular clock model [14]. All the other priors used were left at their default values. Then, the BEAGLE library was used to accelerate computation [15]. For each data set, parallel two independent analyses of 200 million generations were performed, sampling parameters and trees every 10,000 generations. Finally, all the results of analyses were combined using LogCombiner after the removal of a burn-in of 10% of the samples and were checked visually in Tracer [16].

### Phylogeographic migration analyses

To infer the history of viral lineage migration among locations, we performed a Bayesian stochastic search variable selection (BSSVS) approach with a non-reversible discrete-state continuous time Markov chain (CTMT) model using BEAST v1.10.4 [14]. Then we calculated the binary indicator (I) and Bayesian factor (BF) value and explore the potential of cross transmission using SpreaD3 [17]. The country pairs estimated with I > 0.50 and the BF > 6 denoted a migration pathway, with 6 ≤ BF < 10, 10 ≤ BF < 30, 30 ≤ BF < 100, 100 ≤ BF < 1000, and BF > 1000, indicating support, substantial support, strong support, very strong support, and decisive statistical support, respectively [18]. Finally, the migration rates in terms of transmission events per lineage per year were retrieved from the output files of CTMT computation using Tracer [16].

### Phylodynamics analyses of epidemic dynamics

Epidemic dynamics were then reconstructed using posterior analysis of coalescent trees (PACT) v0.9.5 [19] by 0.1 year step ward from these posterior trees clade by clade. The trunk location of each clade was inferred through time [19]. According to the evidence that a high trunk probability indicated the status of an epidemic in terms of a clade, the countries or cities with a trunk probability > 50% were considered as the important epidemic locations, while those with the trunk probability > 50% lasting at least 12 consecutive months, were designated as the dominant epidemic locations [8]. With these considerations, all the statistics were summarized and subjected to epidemic analysis. Independent analyses of 100 resampling replicates were performed to balance differences between the overall sampling resolution and temporal sampling pattern [19].

### Wavelet transforms and multiannual cycles

We employed continuous wavelet transforms (WTs) to explore how the oscillatory behaviour of the number of clades, clusters, and sequences changed over time following the method by García-Carreras et al.’s method [20]. The WT as implemented in R package “WaveletComp” was used following the code described in previous study [20]. Conforming to the concept of García-Carreras, et al. on multianual cycles, we focused on timescales more than 1.5 years [20].

### Transmission cluster delimitation and source identification using E-gene-based genotyping combined with genomic epidemiology

To track and quantify the dengue outbreaks in Chinese mainland and characterize their dynamics and epidemic features of the viral population at the clade level, we further delimitated the transmission clusters of DENV-1 at the clade level in Chinese mainland based on their phylogenic relationship under the E-gene-based genotyping framework of DENV-1 [7], with reference to Louis et al.’ study on the epidemic of SARS-CoV-2 [21]. Concretely, the transmission clusters were assigned up to the following multiple criteria: (1) In the topological structure of E-gene-based phylogenic tree of a certain clade, a transmission cluster corresponded to an independent branch which contained at least one DENV-1 strain isolated from the indigenous case in China. (2) If more than two indigenous strains were included, they were identified as a common ancestor or descendant imported from a shared single source into China.

Meanwhile, by assessing the bootstrap value of transmission cluster and its parental strains (potential importation source) supporting by the topological structure, we categorized the robustness of transmission cluster potential importation source into four levels as follows: (1) Strong support: defined as the support by the topological structure with a bootstrap values ≥ 90; (2) Support: defined as the support by the topological structure with a bootstrap values between 70 and 90; (3) Uncertain: defined as the support by the topological structure with a bootstrap values < 70 and; (4) Unknown: defined as both the topology and the bootstrap values cannot give evidences of the importation source. Then, we named the designated transmission cluster following the format of [x]-[y], where [x] denoted the clade name, and the consecutive Arabic letters in [y] represented the cluster along the phylogenic tree from the root to tip. For example, transmission cluster 1E1-CN1 represented the first transmission cluster of clade 1E1 emerging in China.

Furthermore, we focused on the designated transmission clusters with DENV-1 isolates that appeared consecutively in Chinese mainland for years. To discriminate the transmission relationships of these isolates, we performed phylogenic inference under the E-gene-based genotyping framework reconciling with the genome epidemiology using 362 genomes belonging to the clades mainly circulated in Chinese mainland. These genomes were collected from previous studies and assembled in the present study. Among these isolates of the DENV-1 genomes, we further obtained the epidemiological data of dengue cases in Tangjing Street in Guangzhou from 2014 to 2015 from the field survey sponsored by the Guangzhou Center for Disease Control and Prevention.

### Transmission chains reconstruction from the time-resolved genomic phylogeny

The transmission chains of these lineages were reconstructed from the time-resolved genomic phylogeny using enumeration and sampling algorithm developed by Matthew and Caroline, who allow it available in R package ‘STraTUS’ [22]. Then, for the genomes of DENV-1, the construction procedure of transmission trees were generated by the function ‘tt.generator’ with the default parameter and the transmission chains were visualized using R package ‘ggraph’. Finally, the single-nucleotide polymorphisms (SNPs) of DENV-1 E-genes and genomes were analyzed to explore the molecular mechanisms of the lineages epidemic in consecutive years in China.

## Results

### DENV-1 predominantly epidemic in Chinese mainland especially in Guangdong Province from 1990 to 2019

DENV has posed a growing threat on the public health in Chinese mainland since 1990 (Additional file 3: Figure S1a). Before 2013, the epidemics of dengue fluctuated periodically with an obvious peak every 4‒7 years [6] (Additional file 3: Figure S1a). In 1995, 1999, 2002, and 2006, more than 1000 dengue cases were reported in China (Additional file 3: Figure S1a). When it came to the years between 2013 and 2019, however, dengue outbreaks have occurred continuously with more than 1000 reported cases annually in China (Additional file 3: Figure S1a). Notably, four dengue serotypes (DENV-1, 2, 3, and 4) were reported during these epidemics, and DENV-1 became predominant in recent years (Additional file 3: Figure S1b). DENV-1 cases were reported in about 70% (21/30) of the years during 1990‒2019, while the cases occurred even continuously during the two periods of 1997‒2003 and 2013‒2019 (Additional file 3: Figure S1b). It was the same with DENV-2 the second most-frequent serotype detected in China, occurring continuously during the period of 2013‒2019 in Guangdong (Additional file 3: Figure S1b). DENV-3 and DENV-4 have generated 6 local outbreaks as well (Additional file 3: Figure S1b). Noteworthily, there is a trend of co-circulation with multi-serotypes, rather than a simple epidemic trend of cross-serotypes. For example, DENV-1, 2, and 3 occurred in 2013 and 2019, and four serotypes co-circulated simultaneously in 2016 (Additional file 3: Figure S1b).

A total of 92,995 indigenous dengue cases had been reported in Chinese mainland from 1990 to 2019, and Guangdong became a major focal region for dengue epidemic region, accounting for 69.7% (75,350 cases) of the total and representing an increasing epidemic trend of dengue, especially since 1990 (Additional file 3: Figure S1a,c). The epidemic broke out locally across the whole Guangdong Province (Additional file 3: Figure S1d), and 89.7% (56,345 cases) of the cases were reported from Guangdong‒Hong Kong‒Macao Greater Bay Area including Guangzhou, Shenzhen, Foshan, Zhongshan, and Dongguan. Among them, in particular, Guangzhou reported 48,248 cases, accounting for 76.8% of the total (Additional file 3: Figure S1d). The other five provincial-level administrative divisions (PLADs) in China documenting more than 1000 cases reported were all located in the southeast coastal areas or along the southwest border of the country: Yunnan (9890 cases, 10.6%), Fujian (3555 cases, 3.8%), Jiangxi (1018 cases, 1.1%), and Guangxi (1001 cases, 1.1%) (Additional file 3: Figure S1c).

### Complexity of population structure of DENV-1 epidemic revealing under the E-gene-based global genotyping framework

**Fig. 1:**
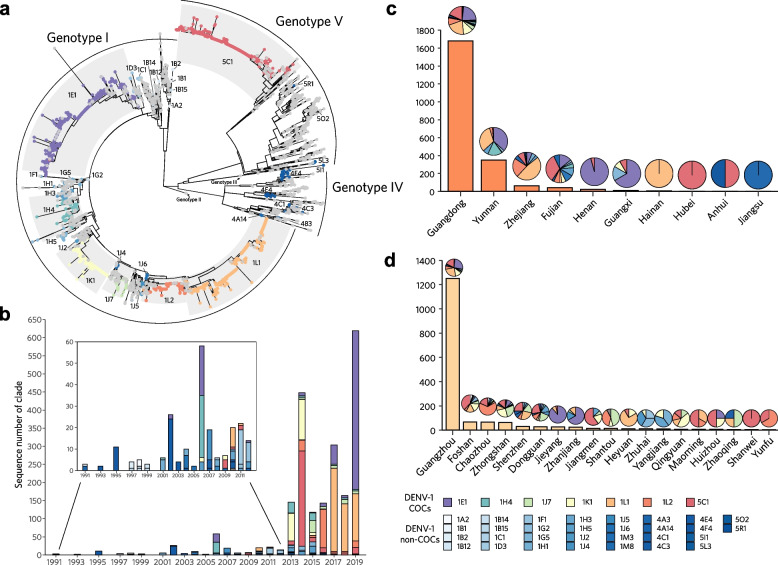
The population structure of DENV-1 circulating in Chinese mainland was designated through the E-gene-based global genotyping framework of DENV-1 [5]. **a** Maximum likelihood phylogenetic analyses show that there are 38 clades labeled in color circulating in Chinese mainland, while the other clades are labeled in gray. **b** Histogram of DENV-1 clades circulating in Chinese mainland from 1990 to 2019. The inset is a partial enlargement view during 1990‒2012. **c, d** Distribution of reported DENV-1 strains in Chinese mainland and Guangdong Province, respectively. Pies are slices colored by the composition of DENV-1 clades

After recognition from the phylogeny of E-gene-based genotyping framework [7], the viral populations in Chinese mainland, were found to contain 38 clades belonging to 22 subgenotypes of all 3 globally and broadly circulating genotypes I, IV and V of DENV-1 (Fig. 1a, Additional file 4: Figure S2, Additional file 5: Table S3). Notably, the subgenotypes and clades of DENV-1 recorded in China were discretely distributed with distinct focuses (Additional file 4: Figure S2a). The number of sequences in the designated clades was distributed with a power-law characteristic, with more than 80% of them concentrated in 7 clades: 1E1, 1L1, 5C1, 1K1, 1L2, 1H4, and 1J7, which were thus named as the Clades of Concern (COCs) in this study. Meanwhile, 12 clades containing sequences between 5 and 50 were classified as the Clades of Interest (COIs), and 19 clades containing sequences < 5 as the Clades under monitoring (CUMs) (Additional file 5: Table S3).

In addition, in order to exploring the cross-transmission mechanisms, we further identified 189 clusters of 38 clades circulating in China, including 126 clusters in the 7 COCs, concretely, 17 clusters in 1E1, 25 in 1H4, 17 in 1J7, 6 in 1k1, 15 in 1L1, 23 in 1L2, and 23 in 5C1 (Additional file 6: Table S4). Similar with the number of clade sequences, the number of cluster sequences also followed a power-law characteristics in its distribution (Additional file 4: Figure S2b and S2c, Additional file 6: Table S4).

The dynamics of DENV-1 population structure at the clade level in the dengue epidemics in Chinese mainland from 1990 to 2019 was showed in Fig. 1b. The main clades responsible for DENV-1 outbreaks were COIs 1B1 and 4A3 in 1991, COI 4F4 in 1995, COI 4E4 in 2002, COCs 1E1 and 1H4 in 2006, COCs 1K1 and 5C1 in 2014, and COCs 1E1 and 1L1 in 2019 (Fig. 1b, Additional file 5: Figure S3a). From 2013 to 2019, both the number of sequences and the diversity of clades of DENV-1 were significantly increased, with several clades showing persistent multi-year prevalence (Fig. 1b, Additional file 5: Figure S3a). Similar to the cases surveillance data analyzed above (Additional file 3: Figure S1), the high diversity of genotypes, subgenotypes, and clades of DENV-1 was found mainly distributed in the PLADs in the southeast coastal regions (Guangdong, Fujian, and Zhejiang) or near the southwest border of China (Yunnan and Guangxi) (Fig. 1c, Additional file 7: Figure S3b; Additional file 8: Figure S4a,b,c), which were recognized as the expanding epidemic regions in previous studies [2]. Meanwhile, the major cities in the Guangdong‒Hong Kong‒Macao Greater Bay Area including Guangzhou, Shenzhen, Foshan, Zhongshan, and Dongguan were also found with higher diversity of DENV-1 population (Fig. 1d; Fig. S3c, Fig. S4d,e,f). Regarding the clade, these provinces and cities were subject to a wide distribution of COCs. Interestingly, the clusters of COCs were focal, clearly as well as 12 cities in the area reported a presence of its 5C1-CN4 (94 sequences), 11 reported a presence of its 1K1-CN6 (125), 9 reported a presence of its 1L1-CN4 (121), and 5 reported a presence of its 1E1-CN16 (343) (Additional file 9: Figure S5, Additional file 6: Table S4).

### Epidemic periodicity of DENV-1 in Chinese mainland

**Fig. 2:**
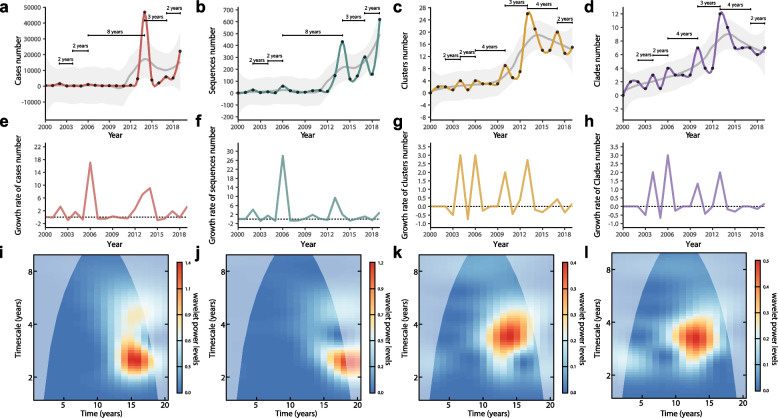
DENV-1 epidemic periodicity in Chinese mainland represented by the dynamics of the number, growth rates, and estimated of multiannual periodicities of the cases, sequences, transmission clusters, and clades. **a-d** Yearly time series of the number of cases (**a**), sequences (**b**), transmission clusters (**c**), and clades (**d**). **e–h** Yearly time series of the growth rates of the number of cases (**e**), sequences (**f**), transmission clusters (**g**), and clades (**h**). **g-i** Estimates of multiannual periodicities of the number of cases (**i**), sequences (**j**), transmission clusters (**k**), and clades (**l**) using the models driven by simple seasonal sine curves. Edge effects in the wavelet transforms may influence results before and after the purple-gray shadow in i, j, k, and l

Further exploring the epidemic periodicity of DENV-1, we found obvious consistent fluctuations in the elevations of cases, clades, clusters, and sequences numbers reported in Chinese mainland during 1990‒2019 (Fig. 2a-d), with the growth rates fluctuating alternately around zero (Fig. 2e-h). The number of clades and clusters in dynamics presented a cyclical pattern of 2 to 4 years, peaking in 2002, 2004, 2006, 2010, 2013, 2017, and 2019, with the most significant growth trend around 2013 (Fig. 2c,d,g,h). But the numbers of cases and sequences were without any peak in 2010, resulting in a long cyclical period between 2006‒2014 (Fig. 2a,b,e,f). The estimated of multiannual periodicities inferred from the number of clades, clusters, cases and sequences were 3.11, 3.45, 2.52 and 2.52 years, respectively (Fig. 2i-l). Compared with the number of cases and sequences (probably influencd by edge effects), the epidemic periodicity inferred from the number of DENV-1 genetic populations including clades and clusters was more robust (Fig. 2i-l). These results indicated that DENV-1 had an epidemic periodicity of about 3 years in China (Fig. 2k-l).

### Phylogeographic inference of introducing and diffusing routes of COCs in Chinese mainland

**Fig. 3:**
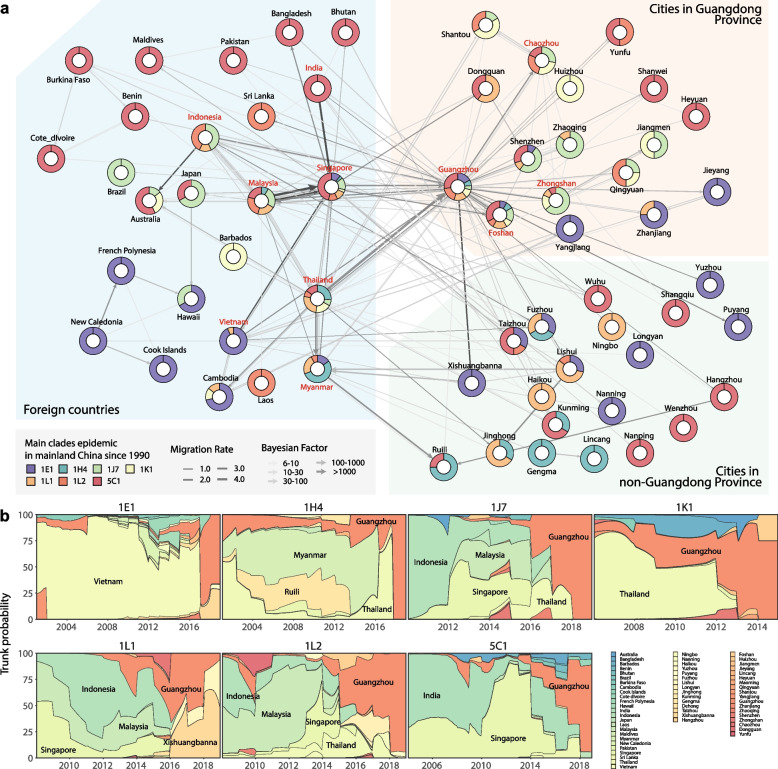
Introducing and diffusing routes of the seven COCs including 1E1, 1H4, 1J7, 1K1, 1L1, 1L2, and 5C1 in Chinese mainland inferred by BSSVS. **a** Phylogeographic reconstruction of the introduction and diffusion events of the seven COCs in Chinese mainland. Myanmar, Vietnam, Thailand, Singapore, Malaysia, Indonesia, and India, as well as Guangzhou, Zhongshan, Foshan, and Chaozhou in China are highlighted in red bold font as these countries/cities occurred more than ten migration events. **b** Dynamics and trends of the epidemic trunks of the seven COCs including 1E1, 1H4, 1J7, 1K1, 1L1, 1L2, and 5C1, representing the proportion of PACT-inferred dominant epidemic countries/cities changed dynamically along with different years

To explore the introducing and diffusing patterns of the seven COCs in Chinese mainland, the seven timed phylogenic trees have been established under the E-gene-based global genotyping framework of DENV-1 using the ancestral state reconstruction of non-reversible discrete spatial locations (Fig. 3a, Additional file 10: Figure S6). The transmission routes belonging to the introduction or diffusion were discerned based on the transmission parameters of the source-sink relationship between two epidemic locations, which were inferred using BSSVS (Fig. 3, Additional file 11: Table S5). The largest number of migration events occurred in the COC 5C1 (56), followed by COCs 1E1 with 34, 1J7 with 26, 1L1 with 25, IL2 with 21, 1H4 with 16, and 1K1 with 14 (Additional file 11: Table S5).

Except for COC 1K1, all the other 6 COCs were introduced on their own routes into Chinese mainland with strong support and migration rates > 1, For instance, 1L1 was introduced from Thailand to Guangzhou with the highest migration rate of 4.68 events/years, 5C1 from Singapore to Guangzhou with the migration rate of 2.04, and 1H4 from Myanmar to Ruili, 2.02 (Fig. 3a, Additional file 12: Figure S7a, Additional file 11: Table S5). After observing the number of involved migration events, as expected, we found that the introduction of seven DENV-1 COCs in Chinese mainland were mainly imported from the attributed countries/areas of the Great Mekong Subregion-China (GMS-China), Southeast Asia (SEA), South Asia Subcontinent (SASC), and Oceania (OCE) [7] (Fig. 3, Additional file 12: Figure S7). Among them, Malaysia, Thailand, and Singapore were indicated to be the most active importing sources, each having more than 8 introduction routes of the COCs into Chinese mainland (Fig. 3, Additional file 11: Table S5, Additional file 12: Figure S7).

On the entry side, the border cities in southwest China and coastal cities in southeast China, were the major receipting hubs (Fig. 3, Additional file 12: Figure S7), and the introduction routes of involved entry cites even differed. Noticeably, Guangzhou was the most dominant receipting hub in China, with the most routes widely from GMS-China, SEA, and SASC countries such as Thailand, Singapore, Vietnam, Malaysia, Indonesia, and India. Meanwhile, the introduction sources to Ruili and Xishuangbanna in Yunnan Province, China were mainly from the countries of GMS-China region like Myanmar, to Taizhou in Zhejiang and Fuzhou in Fujian, China were mainly from the SEA countries like Malaysia, Singapore, and Indonesia (Fig. 3, Additional file 11: Table S5, Additional file 12: Figure S7).

For the diffusion of COCs in Chinese mainland, a pattern was evidently observed that they diffused from the cities in the Guangdong‒Hong Kong‒Macao Greater Bay Area to the cities in the east and west of Guangdong and other PLADs. Among them, the city of Guangzhou had the largest number of introduction routes and internal diffusion routes. It played a crucial role in the process of DENV-1 importation from abroad and diffusion within Guangdong Province and even beyond in China. Specifically, it facilitated diffusion to Foshan, Shenzhen, and Zhongshan in the Guangdong‒Hong Kong‒Macao Greater Bay Area, as well as Yunfu, Chaozhou, Zhaoqing in the eastern and western regions of Guangdong. Additionally, it extended its reach to cities in other PLADs including Hangzhou and Xishuangbanna (Fig. 3, Additional file 11: Table S5, Additional file 12: Figure S7).

### Invasion and diffusion process of the COCs 5C1 and 1K1 responsible for the 2014 large outbreak in Guangdong Province

**Fig. 4:**
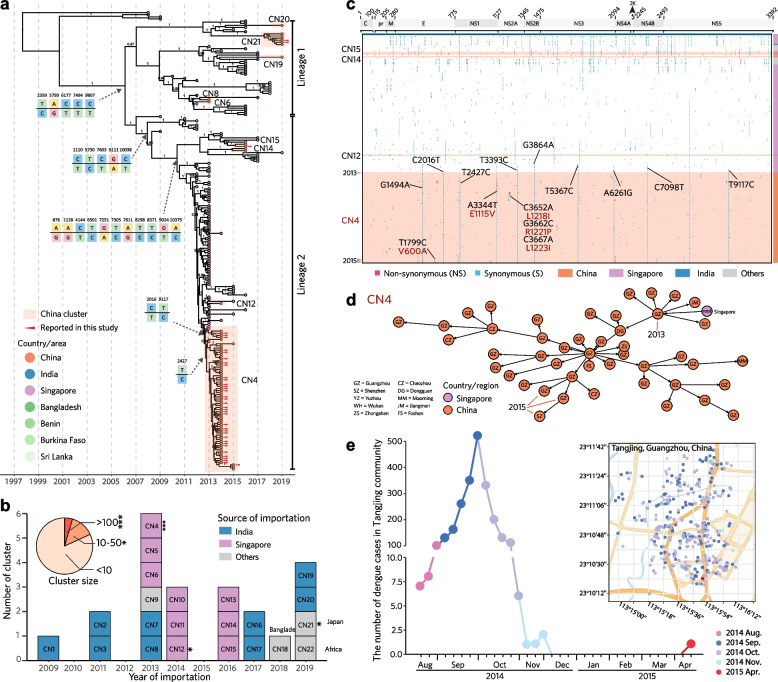
Invasion and diffusion of COC 5C1 of DENV-1 in Chinese mainland. **a** Maximum clade credibility phylogeny of COC 5C1 based on the genomes. According to the topological structure and bootstrap values of the E-gene-based tree, COC 5C1 was further divided into 23 transmission clusters as showed in Chinese mainland showing in Additional file 10: Figure S6g. The present genome-based phylogenetic tree has the same clustering recognition ability as the E-gene-based tree, but it has significantly better resolution for discriminating the exact transmission process of each cluster. The critical SNPs, representing the evolution of clusters during their transmission process, are indicated by arrows at the cluster differentiation nodes on the left of the phylogenetic tree. Taking SNP 2359 as an example, it is T in the genomes of the upper branch, while it is C in the genomes of the lower branch. **b** Temporal dynamics of the designated transmission clusters of 5C1 based on its importation source and year. The inserted pie chart shows the composition ratio of 5C1 clusters with different size. **c** Genome-wide SNPs analysis of the lineage of 5C1 traced from Singapore. Only the positions with specific SNPs found in the strains of cluster 5C1-CN4 isolated in Chinese mainland were shown, with the SNPs of non-synonymous (NS) in blue and synonymous (S) in red. **d** Reconstructed genome-based transmission chain of 5C1-CN4 circulating in Chinese mainland. **e**. Spatiotemporal distribution of the indigenous cases observed in Tangjing Street, Guangzhou, China from Nov. 27th, 2014 to Apr. 4th, 2015. The cases reported in different month are shown in different color. The first indigenous dengue case (2015/KT827378) reported in Guangdong Province, China on Apr. 4th, 2015, is shown in red

COC 5C1 was mainly responsible for a larger DENV-1 outbreak in Guangdong Province, China in 2014 (Fig. 1b), and its strains were isolated for consecutive years from 2013 to 2015 in Guangzhou (Additional file 5: Figure S3a). To explore the invasion and diffusion pattern of 5C1 in the province, we further performed the analyses of phylogeny using E gene and genome, combined with identification of genome-wide SNPs and genome-based transmission chains, after contextualizing them within the epidemiological information, were further performed. We identified a total of 23 clusters were identified from the 292 epidemic strains of COC 5C1 recorded in Chinese mainland, of which the largest cluster 5C1-CN4 had 221 strains isolated in 2014 and spread to other 14 cities in Chinese mainland (Fig. 4a, Additional file 9: Figure S5). Surprisingly, except the origin strain 2005/JQ922548 isolated in India, all the other 5C1 strains exhibited the same distinguishing feature of a 21 nucleotide (nt) deletion in the highly-variable region (HVR) of the 3’-end untranslated region (3’-UTR) (21nt-dHVR-3’-UTR) (Additional file 13: Figure S8). From the phylogenetic tree, 5C1 was observed to originate from India and then obviously evolved into two different lineages (Fig. 4a). The trunk strains of the lineage 1 were traced from India, and in total, its 11 transmission clusters were identified spreading in Chinese mainland from 2010 to 2019 (Fig. 4a). Among them, nine of the clusters were evidently traced from India, but the introducing source of 5C1-CN21 was unknown (Fig. 4a, b). Meanwhile, the trunk strains of the lineage 2 were traced from Singapore, and in total its 12 transmission clusters were identified spreading in Chinese mainland from 2013 to 2019 (Fig. 4a). Among them, nine clusters were evidently traced from Singapore, with the cluster 5C1-CN4 ranked first in 5C1 introduction routes and its strains mostly isolated in the 2014 large outbreak in China (Fig. 4a, b).

Surprisingly, we even observed that the continuous evolution of all 5C1 strains with 21nt-dHVR-3’-UTR had resulted in a series of specific SNPs, which characterized its transmission and adaptation process from India to Singapore and China (Additional file 13: Figure S8). The SNPs T2359C, A5799G, C6177T, C7494T, and C9807T represented the separation of Lineage 1 and Lineage 2 (Fig. 4a). The foundational strains were 2009/JN903581/India in Lineage 1 and 2009/JF960211/Singapore in Lineage 2, while the strain isolated in Guangzhou, China in 2019 persistently conserved these SNPs of Lineage 1 (Fig. 4a). Following the foundation of Lineage 2 sourced from Singapore, the strain JQ917404 isolated in India in 2009 developed with several SNPs including C1110T, T5730C, C7603T, G9111A, and C10038T, indicating a complex cross-transmission and adaptation of 5C1 population (Fig. 4a). Consequently, the master strains caused the large outbreak in Singapore during 2012‒2013 displaying the SNPs including A876G, A1128G, C4144T, T6501C, G7251A, T7305C, A7611G, T8298C, T8371C, G9024T, and A10375C (Fig. 4a). Later, the strains 2013/KX225487 and 2014/GZ8_0027 in 5C1-CN4 isolated in Guangzhou preserved the same SNPs developed in KM403633 and KM403634 isolated in Singapore in 2013, including C2016T and T9117C (Fig. 4a, c). Subsequently, the local master strains of 5C1-CN4 caused the large outbreak in Guangzhou in 2014 and the strain 2015/KT827378 evolved with the specific SNP T2427C (Fig. 4, Additional file 10: Figure S6). Meanwhile, the population size of the Lineage 2 sourced from Singapore was inferred with two rapid population expansions in 2013 and 2014, respectively, in accordance with the respective large outbreaks in Singapore in 2013, and in Chinese mainland in 2014 (Fig. 4, Additional file 13: Figure S8).

Notably, the largest cluster 5C1-CN4 was introduced from Singapore in 2013 and then became a consecutively transmitted in Guangzhou from 2013 to 2015. The evidence was strongly supported by the topology of both E-gene-based and genome-based phylogenic trees (Fig. 4a, b, Additional file 10: Figure S6g). Furthermore, the genome-wide SNPs analyses also verified that 5C1-CN4 was originated from Singapore, showing several same variations including C2016T, T3393C, G3864A, T5367C, A6261G, C7098T, T9117C, A10375C, and 21nt-dHVR-3’-UTR following its root strains. All of them were originally isolated from the large dengue outbreak in Singapore in 2013 [23] (Fig. 4c, Additional file 13: Figure S8). But a distinctive variation T2427C was observed in 5C1-CN4 as well, which indicated that this cluster has been developing to become a local master viral population (Fig. 4c). Moreover, the genome-based transmission chain of 5C1-CN4 further demonstrated that 5C1-CN4 was first introduced from Singapore to Guangzhou in 2013, and consecutively transmitted locally in Guangzhou from 2013 to 2015, during which it diffused from Guangzhou to other cities in Guangdong Province and even other PLADs in China (Fig. 4a, d, Additional file 10: Figure S6g). Moreover, a unique strain of 2015/KT827378 was isolated from the first indigenous dengue case in China through in-depth epidemiological investigation on April 4th, 2015, who resides in Tangjing Street, Baiyun District, Guangzhou. It was for more than four months (Nov 27th, 2014 to Apr 4th,2015) that neither imported nor indigenous dengue cases had been reported in Guangzhou since the 2014 large outbreak, but only between August to November in the year of 2014 had 1338 indigenous dengue cases been reported in this street.

Another important COC of DENV-1 responsible for the 2014 large outbreak in Guangdong Province, China was 1K1 with 6 designated transmission clusters (Additional file 10: Figure S6a, Additional file 14: Figure S9). Despite 1K1 strains were simultaneously reported in Thailand, Cambodia, Barbados, Australia, and Chinese mainland, the precise sources of all six transmission clusters of 1K1 in Chinese mainland could not be inferred by reconstruction of fine-scale genome phylogeny combining with the E-gene-based phylogenic tree, which thereby marked as unknown in the figure (Additional file 10: Figure S6a, Additional file 14: Figure S9). The largest cluster 1K1-CN6 was circulated in the core cities in the Guangdong‒Hong Kong‒Macao Greater Bay Area especially in Guangzhou and Foshan from 2014 to 2015, with the majority of its strains reported in 2014 (Additional file 14: Figure S9a). Inferred from both the E-gene-based and genome-based phylogenic trees of 1K1, the strain 2015/MN018295 isolated from the first dengue patient in Foshan on May 23rd, 2015 [24] was originated from the strain KX458013 isolated in Guangzhou on November 10th, 2014 (Additional file 14: Figure S9), which represented the occurrence of cross-year transmission of the cluster 1K1-CN6 in Guangzhou and Foshan, China. This evidence was also verified by the transmission chain established using genomes of 1K1-CN6. At last, the genome-wide SNPs comparison showed that the strain MN018295 preserved all the specific SNPs of 1K1-CN6 including T279C, C756T, A2175G, T3024C, G5073A, G7527A, A8637G, C9144T, A9210G, and A10116G (Additional file 14: Figure S9).

### Dynamic characteristics of the transmission clusters belonging to the other 5 COCs

Besides the COCs 5C1 and 1K1, a total of 97 transmission clusters were identified using the phylogenic trees for COCs 1E1, 1H4, 1J7, 1L1, and 1L2 (Additional file 6: Table S4, Additional file 10: Figure S6, Additional file 14: Figure S9, Additional file 15: Figure S10). Through the comprehensive analysis of phylogeny combining with epidemiological information for the importation sources of COCs clusters, it was found that 18.8% –100% of the clusters designated in 7 COCs could not be inferred, thereby marked as unknown in this study (Additional file 6: Table S4, Additional file 16: Figure S11). Among those with unknown sources, 44 clusters were from Guangdong Province, a number significantly larger than from other PLADs in China (Additional file 6: Table S4, Additional file 16: Figure S11). Followed by Yunnan Province, it had 13 clusters with unknown sources, of which 9 were in 1H4 and 3 were in IL2 (Additional file 6: Table S4). In terms of the temporal distribution, 8 clusters had cross-year transmission, 7 in Guangdong Province and 1 in Yunnan Province, including 5C1-CN4 and 1K1-CN6 as described above, 1E1-CN16 in Guangdong with unknown sources, and 1L1-CN7 in Yunnan and Zhejiang with the sources from Myanmar (Additional file 15: Figure S10).

As for 1E1-CN16, it was found transmit in Guangzhou during 2018‒2019, and largely reported in 2019 (Additional file 15: Figure S10). The E-gene-based phylogenic tree of 1E1 showed that several strains reported in Guangzhou in 2019 including MN921341 might be originated from the strain MN933727 isolated in Guangzhou in 2018, but the genome of MN921341 was unavailable for in-depth inferring (Additional file 15: Figure S10). Meanwhile, both the genome-based and E-gene-based phylogenic trees inferrably uncovered that the tMRCA of MN933669 isolated in Guangzhou in 2018 with other strains isolated in 2019 were similar to those isolated in 2017, indicating the evidence of these strains continuously circulating in Guangzhou were unconvinced (Additional file 15: Figure S10). Although the genome-wide SNPs analyses verified that the strain 2018/MN933669 gathered within the strains isolated in 2019 and represented several same variations including T2286C, Y886H, and C8034T, there were several specific amino acid variations in E-gene region of the strain MN933669 (Additional file 15: Figure S10). Due to the insufficiency of information of genomes of the strains involved in the other five cross-year transmissions, no further analyses on their transmission were carried out in this study (Additional file 16: Figure S11).

## Discussion

Controlling the expansion of DENV epidemics is challenging. Understanding the mechanism of these expansion process will guide us searching for suitable and sufficient tools to monitoring and blocking. Under the globally unified E-gene-based framework of DENV-1 [5], the present study elucidated the genetic population structure of DENV-1 circulated in Chinese mainland at the spatial-genetic Nation-Clade level, particularly in Guangdong Province. DENV-1 genetically comprises 189 transmission clusters distributed across 38 clades belonging to genotype I, IV, and V of DENV-1. Although this study acknowledges potential biases in the data collection process, such as advancements in sequencing technology and variations in testing capabilities across different cities. Notably, the seven COCs (1E1, 1L1, 5C1, 1K1, 1L2, 1H4, and 1J7) have been primarily accountable for the large dengue outbreaks in Chinese mainland since 1990. This discriminated genetic scheme of DENV-1 provides a solid and convenient foundation for establishing precise monitoring strategies not only in China, but also for the regional cooperation aimed at curbing the rapid spread of dengue.

Since the reemergence of dengue outbreak in Foshan, Guangdong Province, China in 1978, imported cases have been considered as the key driver of dengue epidemics in Chinese mainland [8]. During the COVID-19 pandemic period, unexpectedly, the implementation of border control measures in 2020 led to a significant decrease in dengue incidence observed both in Guangdong and Yunnan provinces, which also suggests that imported cases play a primary role in triggering dengue transmission in Chinese mainland [10, 11]. Our analyses revealed that the active transmission routes were predominantly linked with countries/areas from GMS-China, SEA, SASC, and OCE. Specifically Thailand, Singapore, Vietnam, Malaysia, India, Indonesia, and Myanmar exhibited strong connections to coastal cities located southeastward as well as border cities situated southwestward within Chinese mainland. For example, 1L1 and 5C1 from Thailand and Singapore, respectively, to Guangzhou with a respective higher migration rates of 4.68 and 2.04 events/years. Consistent with numerous previous epidemiological investigations, the primary sources of DENV-1 transborder transmission were mainly from the countries/areas within GMS-China and SEA [8, 25]. It is evident that all transborder transmission destinations were port cities facilitating international trade and communications. Guangzhou, as the capital of Guangdong Province and the south gate of China, stands out as the predominant receipting hub, from which the disease subsequently diffuses to other cities within the province or the country. It is worth noting that several seaports such as Shantou and Zhanjiang have also became significant transborder destinations, potentially due to their involvement in international shipping and fishery industries. Additionally, cities including Xishuangbanna, Ruili, and Jinghong in Yunnan province engage in frequent cross-border trade activities which may have contributed to a substantial number of undetected imported cases.

Furthermore, our study also unveiled the complexity of dengue epidemics—multiple DENV population simultaneously commonly co-circulated in a single epidemic year or in a single epidemic location. Interestingly, we also observed that dengue outbreaks occurring during some specific years were not solely attributed to these dominant COCs alone; up to 10 different clades were reported during the outbreak in 2014. Furthermore, when compared with SARS-CoV-2 and influenza, DENV-1 populations showed a parallel evolutionary trend, indicating longer epidemic periods which extended over several years associated with dangerous clades/clusters confined to certain epidemic countries/regions [19]. Taking clade 5C1 as an example, a total of 23 clusters were identified in Chinese mainland from 2009 to 2019. Among them were the clusters 5C1-CN4 and 5C1-CN21, which were primarily responsible for the large outbreaks in 2014 and 2019, respectively. The presence of disparities in national/regional surveillance and research capacities may lead to the biases in sequence numbering and genetic diversity, thereby rendering a common rule of thumb based solely on source location with higher genetic diversity inadequate for drawing accurate conclusions. Therefore, the high genetic diversity of DENV-1 epidemic in Chinese mainland indicates diverse sources of introduction, with potentially hidden outbreaks in relevant countries/regions. It is urgently necessary to establish an efficient cooperative dengue surveillance system at national/regional scales based on the defined high-resolution viral genetic composition of DENV.

In addition to the annual seasonal dynamics, DENV epidemics exhibit a multiannual periodicity [20, 26, 27]. Willem et al. [26] conducted a study utilizing 18 years of monthly dengue surveillance reports from 273 provinces across 8 countries/areas in Asia, unveiling a periodicity ranging from 2 to 5 years and region-wide synchrony of dengue outbreaks. Specifically, during the periods of 1993‒2002 and 2009‒2010, a strong correlation between the multiannual periodicity and El Niño Southern Oscillation (ENSO) was observed across nearly all provinces. Interestingly, the further clustering based on the synchronization periods of these provinces revealed that city cluster 1 comprises Zamboanga, Davao and Cebu, while city cluster 2 consists of Phnom Penh, Vientiane, Hanoi and Kuala Lumpur [7, 26]. This classification aligns remarkably well with the regional division of DENV established by phylogeny and phylogeography in our previous study [7]. Specifically, cities within city cluster 1 are located in the Philippine region, whereas those within city cluster 2 are in the GMS-China region. Churakov et al. [27] also reported a spatio-temporal synchronized pattern of dengue outbreaks across various Brazilian states from 2001 to 2016. In the present study, an epidemic periodicity of approximately 3 years has been inferred by the number of the designated viral genetic populations including clades and clusters circulating in Chinese mainland. Of course, considering the bias introduced by the underreporting of case notifications and sequencing variations over time and across different regions, further prospective study is needed.

The recent studies on SARS-CoV-2 genome phylogeny have demonstrated the advancements in the quantification of size, spatiotemporal origins, and persistence of genetically distinct transmission clusters. The findings have enhanced our understanding of the rapid epidemic dynamics within pathogen lineages [21, 28]. Meanwhile, the E-gene based global unified framework of DENV-1 establishes a compatible genotyping baseline [7]. We observed that genome phylogeny of DENV-1 exhibited no discernible differences from the E-gene-based baseline in genetic structure identification at the genotype, subgenotype, and clade levels. However, genome epidemiology was also found to demonstrate a significantly enhanced resolution in unraveling ongoing transmission events of dengue and conducting whole-genome SNPs analyses. Therefore, it is convincible for us to combine the genome phylogeney combined with the epidemiological investigation and phylogeographical analyses to elaborate the epidemic mechanisms of dengue in the present study. With these combined means, the transmission clusters 5C1-CN4 and 1K1-CN6 were identified mainly responsible for the large outbreak in 2014 in Guangzhou. 5C1-CN4 was observed to have transmitted definitely locally in Guangzhou from 2013 to 2015. The 5C/5C1 strains, which exhibits a 21nt-dHVR-3’-UTR deletion, was initially identified in India [29] and subsequently played a pivotal role in the significant outbreaks that occurred in Singapore (2013) [23] and China (2014). Furthermore, by discriminating the two lineages of 5C1, it was found that Lineage 1 was originated from India, and Lineage 2 was traced back to Singapore. The finding was strongly supported by the evidences discovered by both the topology of phylogenic trees and the genome-wide SNPs that 5C1-CN4 of Lineage 2 was introduced into Chinese mainland from Singapore in 2013, and subsequently developed to be a local master viral population with a distinctive variation T2427C locally in Guangzhou from 2014 to 2015. What’s more, the strain 2015/KT827378 was also identified to be one of the DENV-1 5C1-CN4 strain isolated from the first indigenous dengue case in Guangzhou on Apr. 4th, 2015. The diapause eggs of *Ae. albopictus* can sustain DENV through vertical transmission [30]. Thereby, these results implicated that 5C1-CN4 of DENV-1 had been successfully preserved and locally transmitted through winter, and then started to colonize in Guangzhou during 2013 to 2015. Nearly at the same time, another cluster 1K1-CN6 came out with cross-year transmission locally in Guangzhou and Foshan from 2014 to 2015. Worrisome, seven of eight clusters with cross-year transmission were found in Guangdong except one in Yunnan. ENSO in the previous year may drive dengue epidemics in Guangdong [31]. Interestingly, apart from 1L1-CN1, the epidemics of the other 7 clusters with cross-year transmissions were found to emerge during the ENSO periods of 2014‒2015 and 2018‒2019. This finding suggests that milder winters influenced by ENSO may create favorable conditions for mosquito and arbovirus survival. In addition, the discrimination of clusters with unknown sourcing in our present study also indicates that Guangdong and Yunnan play a key role in the ongoing transition of dengue epidemic patterns in China.

**Fig. 5:**
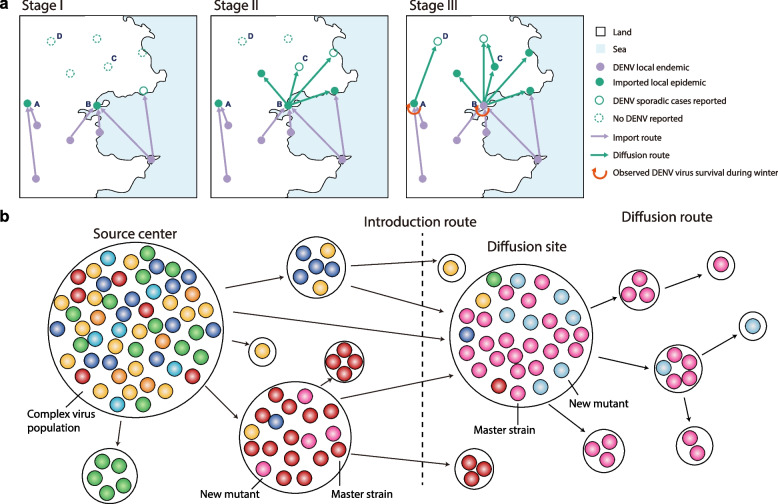
Invasion and diffusion patterns of DENV based on Invasion Biology theories demonstrated in this study. **a** Scheme of three-stages of invasion, colonization, and dissemination of DENV. The white areas indicates lands, and the blue areas indicates oceans. The circles represent the cities, where those in purple indicates the cities are with DENV endemic area or has been transferred to the endemic area, while those in green indicate the cities are without DENV endemic (dengue epidemics driven by the imported cases). The solid circle indicates the presence of large epidemic outbreaks, the hollow circle indicates the presence of sporadic cases, and the dashed circle indicates no reported DENV cases in the city. The purple direction line indicates the importation process from DENV endemic city to the non-endemic. The green direction line indicates the diffusion process of DENV between the non-endemic cities. The red arc arrow indicates that DENV survived the winter in the city and caused a dengue epidemic in the following year. As a result, these cities will be thrust into the ongoing high-risk transition process from epidemic to endemic through DENV’s continuing adaptation. **b** The schematic depicting the population dynamics of DENV during the importation and diffusion process, under the contextualization with the founder effects and population bottlenecks of mosquito-borne arboviruses, developed from Weaver et al. [32]

Weaver et al. [32] illustrated the founder effects and population bottlenecks of mosquito-borne arboviruses. Developed from this conception and contextualized with Invasion Biology theories, we try to explore the invasion and diffusion patterns of DENV. Taking DENV-1 epidemic in Guangdong particularly in Guangzhou as an example, three-stages of invasion, colonization, and dissemination of DENV has been demonstrated (Fig. 5). In stage I, DENV epidemics are featured with multi-point and multi-year importations from endemic areas to non-endemic areas. During this period, local outbreaks are primarily driven by the imported cases. In stage II, following the local outbreaks in the first stage, DENV breaks out in a large-scale, spreading between non-endemic cities may occur due to persistent and robust importations of more cases. In stage III, following an extended period of introduction and multiple outbreaks of DENV, the colonization of pathogens may commence. DENV may survive over winter in the local cities and subsequently trigger a disease epidemic in the following year, thereby propelling it into an ongoing high-risk transition process from epidemic to endemic through continuous adaptation (Fig. 5a). If pathogens successfully colonizes themselves in non-endemic cities during stage III, it may result in further local dissemination and transmission of the disease. This signifies a transition from an "epidemic" to parallel "epidemic" and "endemic" patterns in those non-endemic cities, potentially ending up with its eventual endemicity or localization (Fig. 5a).

Over the past two decades, the presence of cross-year overwintering transmissions and unknown sources of transmission clusters found in China indicate an ongoing process of local dissemination of dengue. This trend can be interpreted as an event that significantly delineates the stages of DENV invasion-colonization-dissemination according to Invasion Biology theory (Fig. 5). Guangdong Province, particularly its capital city of Guangzhou, has experienced a notable influx of imported cases and frequent dengue outbreaks since 1990. In this city, as previously described, emerged a clear local consecutive transmission process of 5C1-CN4 of DENV-1 from 2013 to 2015. Specifically, the introduction of DENV from Singapore in 2013 [23] (the main strain responsible for the large outbreak in Singapore was DIII 13.13, while the strain introduced into China was DIII 13.09—note that this genome-based genotype III is equivalent to genotype V of DENV-1 according to the unified global E-gene-based genotyping framework [7]) led to the emergence of a distinct local main strain with variation T2427C contributing to the large outbreak in Guangzhou in 2014; subsequently, DENV successfully overwintered and resulted in detection of first indigenous dengue case in Guangzhou during 2015.

The conceptual scheme in Fig. 5b illustrates the population dynamics of DENV transmission during the process. These results strongly suggests that dengue epidemics in Guangzhou is running into the stage III of the pattern. Although rare observation of DENV interannual winter events, we still need to attach great importance to take practical epidemic control measures to contain its transition from imported colonization to detrimental dissemination. Meanwhile, epidemic patterns of dengue in China are also influenced not only by the complex interplay of global and regional epidemic trends but also by other factors such as persisting climate changing, periodic occurrence of ENSO, unplanned urbanization, and globalization [2, 31, 33]. This situation makes us aware that China, currently as an important emerging epidemic country of dengue, needs to take actions based on a more intricate epidemic patterns of dengue rather than the simple importation-driven model. It is crucial for the Chinese government to enhance dengue early warning monitoring and control systems for national/regional cooperation to impede DENV transition from epidemic to endemic and ultimately prevent and control the rapid spread of dengue scientifically and efficiently.

## Conclusions

Under the globally unified E-gene-based genotyping framework, this study systematically characterized the complex genetic population structure of DENV-1 epidemic in Chinese mainland from 1990 to 2019. It revealed the main patterns of introduction and diffusion of COCs, as well as the periodic dynamics of dengue epidemics in China. The COC 5C1 was primarily responsible for a large outbreak in Guangdong Province in 2014, and its largest cluster, 5C1-CN4 in lineage 2, showed to have been consecutively transmitted processes in Guangzhou from 2013 to 2015. Contextualized within the Invasion Biology theories, a derived three-stage model enhances our understanding of dengue spreading patterns, which involved the stages of including invasion, colonization, and diffusion. These findings highlight the potential ongoing transition trends from epidemic to endemic, thereby offering a valuable insight into the prevention and control of rapid dengue spread both within China and globally. It is imperative to establish early warning systems and implement appropriate regional or even global collaborative strategies in order to effectively address this issue.

### Supplementary Information

Additional file 1: Table S1. Summary of indigenous dengue cases reported in Chinese mainland during 1990‒2019.

Additional file 2: Table S2. Characterization of the assembled genomes of DENV-1 circulated in Guangdong Province in this study.

Additional file 3: Figure S1. Epidemiological characteristics of DENVs in Chinese mainland during 1990‒2019. a, Dengue cases number reported in Guangdong (red) and other province (blue) in Chinese mainland during 1990‒2019. b, Serotypes of indigenous dengue reported in Guangdong (red) and other provinces (blue) in Chinese mainland during 1990‒2019. Serotypes data were obtained from local authorities archived in Guangdong Provincial Center for Diseases Control and Guangzhou Center for Diseases Control. Serotype identification is accomplished through serum testing techniques or molecular identification methods employed by local authorities. c, Epidemic provinces with indigenous dengue cases in Chinese mainland during 1990‒2019. The left bar shows the number of involved provinces of 4 serotypes of DENV, respectively. d, Epidemic cities with indigenous dengue cases in Guangdong Province during 1990‒2019. The left bar shows the number of involved cities in Guangdong Province circulating 4 serotypes of DENV, respectively. The color scale in inner circle core represents case number. Red, green, blue, and pink quarter pies represent 4 serotypes of DENV, respectively.

Additional file 4: Figure S2. Distribution of the sequence number in the designated clades and clusters circulated in Chinese mainland. a, Blue bars show the sequence number in each clade. The inset shows the corresponding cumulative distribution functions (CDFs) of clade sequence number on double logarithmic axes. Values show coefficients of power-law distributions fitted to the sequence number in clades. Clades of Concern (COCs), Clades of Interest (COIs), and Clades under monitoring (CUMs) were classified based on the thresholds of 50 and 5 sequences, corresponding the respective cumulative 80% and 50% sequences. b, Green bars and inset show the sequence number in each designated transmission cluster and the corresponding CDFs of cluster sequence number on double logarithmic axes. c, Partition of the designated clusters sized by their sequence numbers.

Additional file 5: Table S3. Summary of the Clades of Concern (COCs), Clades of Interest (COIs), and under monitoring (CUMs) of DENV-1 circulated in Chinese mainland.

Additional file 6: Table S4. Characterizing the spatio-temporal distribution and inferring the importing sources of the transmission clusters epidemic in Chinese mainland.

Additional file 7: Figure S3. The composition and distribution of seven COCs of DENV-1 in Chinese mainland. Stacked barplots of proportion are colored by seven COCs along time from 1990 to 2019 (a). Stacked barplots of sequence number are colored by seven COCs showing their distribution in provinces in Chinese mainland (b) and cities in Guangdong Province (c).

Additional file 8: Figure S4. Shannon’s index of genotype (a, d), subgenotype (b, e), and clade (c, f) of DENV-1 circulated in each province in Chinese mainland (a-c) / city of Guangdong Province (d-f).

Additional file 9: Figure S5. The cities were involved in the circulation of seven COCs transmission clusters in Chinese mainland from 1990 to 2019. Different cities are represented by color.

Additional file 10: Figure S6. phylogenetic trees of COCs 1E1 (a), 1H4 (b), 1J7 (c), 1K1 (d), 1L1 (e), 1L2 (f), and 5C1 (g) established under the E-gene-based global unified framework of DENV-1. The designated transmission clusters circulated in Chinese mainland are shaded in apricot. The colored circles indicated the locations of strains.

Additional file 11: Table S5. The introduction and diffusion routs of the seven COCs in Chinese mainland inferred by BSSVS.

Additional file 12: Figure S7. Summarizing the migration events of seven COCs of DENV-1 inferred by BSSVS. a. The estimated migration routes of seven COCs with migration rate > 1.0 events/year. b. Involved countries or cities number of the estimated importation and diffusion routes of seven COCs.

Additional file 13: Figure S8. Sequence alignment and population size estimation of the COC 5C1. a. Multiple sequence alignment for 3’-UTR deletion region of 5C1. b. The dynamic description of the estimated population sizes for 5C1 Lineage 1 (left) and Lineage 2 (right).

Additional file 14: Figure S9. Invasion and diffusion of the COC 1K1 of DENV-1 in Chinese mainland. a. Maximum clade credibility phylogeny of the COC 1K1 based on the genomes. b. Temporal dynamics of the designated transmission clusters of 1K1 based on its importing source and year. The inserted pie chart shows the composition ratio of the size of the clusters of 1K1. c. Genome-wide SNPs analysis of the cluster 1K1-CN6. Only positions with specific SNPs found in the strains of cluster 1K1-CN6 isolated in Chinese mainland were shown, the SNPs of non-synonymous (NS) in blue and synonymous (S) in red.

Additional file 15: Figure S10. Invasion and diffusion of the COC 1E1 of DENV-1 in Chinese mainland. a. Maximum clade credibility phylogeny of the COC 1E1 based on the genomes. b. Temporal dynamics of the designated transmission clusters of 1E1 based on its importing source and year. The inserted pie chart shows the composition ratio of the size of the clusters of 1E1. c. Genome-wide SNPs analysis of the cluster 1E1-CN16. Only positions with specific SNPs found in the strains of cluster 1E1-CN16 isolated in Chinese mainland were shown, the SNPs of non-synonymous (NS) in blue and synonymous (S) in red.

Additional file 16: Figure S11. Characterizing the introduction sources and cross-year transmission of the seven COCs in Chinese mainland. a. Composition of introduction sources were inferred from the transmission clusters of the seven COCs from 2001 to2019. b. Schematic diagram of the transmission clusters of COCs with strains isolated in consecutive years from 2010 to 2019. The isolates number are indicated by pie chart size, and the provinces reported the isolates are showed by pie chart color.

## Data Availability

Data supporting the conclusions of this article are included within the article and its additional fles. The datasets used and/or analyzed during the present study are available from the corresponding author upon reasonable request. The R script used to generate the results in this manuscript are available in the following Git repository: https://github.com/GuoXiang9399/DENV_CN

## Abbreviations

DENV: Dengue virus
kb: Kilobases
RT-PCR: Reverse-transcription polymerase chain reaction
CTMT: Continuous time Markov chain
BSSVS: Bayesian stochastic search variable selection
BF: Bayesian factor
PACT: Posterior analysis of coalescent trees
WTs: Wavelet transforms
SNPs: Single-nucleotide polymorphisms
COCs: Clades of Concern
COIs: Clades of Interest
CUMs: Clades under monitoring
GMS: Great Mekong Subregion countries
SEA: South-East Asia
SASC: South Asia Subcontinent
OCE: Oceania
nt: Nucleotide
HVR: Highly-variable region
3’-UTR: 3’-End untranslated region
NS: Non-synonymous
ENSO: El Niño Southern Oscillation
