# Supplementary material for: Phylodynamics unveils invading and diffusing patterns of dengue virus serotype-1 in Guangdong, China from 1990 to 2019 under a global genotyping framework
Source: Infect Dis Poverty. 2024 Jun 11;13:43. doi: 10.1186/s40249-024-01211-6 (PMC11165891; doi:10.1186/s40249-024-01211-6)
Supplement: Supplementary file 1 — Additional file 1: Table S1. Summary of indigenous dengue cases reported in Chinese mainland during 1990‒2019. [file 40249_2024_1211_MOESM1_ESM.pdf]

Table S1. Summary of dengue cases number in mainland China during 1990-2019.

Year	Total cases no.	Case no. in Guangdong	Case no. in non-guangdong	Guangdong case no./Total no.
1990	376	374	2	99.47
1991	902	371	531	41.13
1992	2	2	0	100
1993	367	359	8	97.82
1994	4	4	0	100
1995	6,836	6,812	24	99.65
1996	2	0	2	0
1997	634	632	2	99.68
1998	490	488	2	99.59
1999	1,868	304	1564	16.27
2000	405	401	4	99.01
2001	375	365	10	97.33
2002	1,606	1,576	30	98.13
2003	93	82	11	88.17
2004	247	49	198	19.84
2005	59	23	36	38.98
2006	1,063	1,022	41	96.14
2007	551	397	154	72.05
2008	254	97	157	38.19
2009	322	19	303	5.9
2010	260	139	121	53.46
2011	160	49	111	30.63
2012	575	474	101	82.43
2013	4,663	2,894	1,796	62.06
2014	46,864	45,189	1,675	96.43
2015	3,858	1,683	2,175	43.62
2016	2,050	544	1,506	26.54
2017	5,893	1,662	4,231	28.20
2018	5,136	3,315	1,821	64.54
2019	22,188	6,024	16,164	27.15
