# Supplementary material for: Phylodynamics unveils invading and diffusing patterns of dengue virus serotype-1 in Guangdong, China from 1990 to 2019 under a global genotyping framework
Source: Infect Dis Poverty. 2024 Jun 11;13:43. doi: 10.1186/s40249-024-01211-6 (PMC11165891; doi:10.1186/s40249-024-01211-6)
Supplement: Supplementary file 2 — Additional file 2: Table S2. Characterization of the assembled genomes of DENV-1 circulated in Guangdong Province in this study. [file 40249_2024_1211_MOESM2_ESM.pdf]

Table S2. Characterizations of the sequences of DENV circulated in Guangdong sequencing in this study.

No.	Severity*	Gender	Age	Accession No.**	Year	Strain name in this study	Sequence	Serotype	Genotype	Clade
1	/	/	/	KX225491	2006	2006|KX225491|China	IonTorrent, Sanger	1	I	1H4
2	/	/	/	KX225490	2013	2013|KX225490|China	IonTorrent, Sanger	1	I	1H4
3	/	/	/	KX621251	2013	2013|KX621251|China	IonTorrent, Sanger	1	I	1K1
4	/	/	/	KX225489	2013	2013|KX225489|China	IonTorrent, Sanger	1	I	1K1
5	/	/	/	KX225488	2013	2013|KX225488|China	IonTorrent, Sanger	1	I	1K1
6	/	/	/	KX458013	2014	2014|KX458013|China	IonTorrent, Sanger	1	I	1K1
7	/	/	/	KX620455	2014	2014|KX620455|China	IonTorrent, Sanger	1	I	1K1
8	/	/	/	KX458014	2014	2014|KX458014|China	IonTorrent, Sanger	1	I	1K1
9	/	/	/	KX620454	2014	2014|KX620454|China	IonTorrent, Sanger	1	I	1G5
10	/	/	/	NMDCN0002PHC	2016	2016|GZ-14861|China	Sanger	1	I	1L2
11	/	/	/	NMDCN0002PHD	2016	2016|GZ-15334|China	Sanger	1	I	1L2
12	/	/	/	NMDCN0002PHE	2016	2016|GZ-18241|China	Sanger	1	I	1L2
13	/	/	/	NMDCN0002PHB	2016	2016|GZ-545|China	Sanger	1	I	1L2
14	/	/	/	NMDCN0002PHF	2017	2017|GZ-29492|China	Sanger	1	I	1L1
15	/	/	/	NMDCN0002PHG	2017	2017|GZ-40277|China	Sanger	1	I	1L1
16	/	/	/	NMDCN0002PHH	2017	2017|GZ-8Y2|China	IonTorrent, Sanger	1	I	1L1
17	/	/	/	NMDCN0002PHI	2017	2017|GZ-8Y3|China	IonTorrent, Sanger	1	I	1L1
18	/	/	/	NMDCN0002PHJ	2017	2017|GZ-8Y4|China	IonTorrent, Sanger	1	I	1L1
19	/	/	/	NMDCN0002PHK	2017	2017|GZ-8Y5|China	IonTorrent, Sanger	1	I	1L1
20	/	/	/	NMDCN0002PHL	2017	2017|GZ-CSH|China	Sanger	1	I	1L1
21	/	/	/	NMDCN0002PHM	2017	2017|GZ-HYY|China	Sanger	1	I	1L1
22	/	/	/	NMDCN0002PHN	2017	2017|GZ-LHQ|China	Sanger	1	I	1L1
23	/	/	/	NMDCN0002PHO	2017	2017|GZ-p1-consensus|China	Sanger	1	I	1L1
24	/	/	/	NMDCN0002PHP	2017	2017|GZ-xue-consensus|China	Sanger	1	I	1L1
25	/	/	/	NMDCN0002PHQ	2017	2017|GZ-YLY|China	Sanger	1	I	1L1
26	/	/	/	NMDCN0002PHR	2017	2017|GZ-ZRH|China	Sanger	1	I	1L1
27	/	/	/	KX621250	2014	2014|KX621250|China	Illumina	1	I	1L2
28	/	/	/	KX225492	2014	2014|KX225492|China	Illumina	1	I	1L2
29	/	/	/	KX225493	2014	2014|KX225493|China	Illumina	1	I	1L2
30	/	/	/	KX621253	2016	2016|KX621253|China	Illumina	1	I	1L2
31	/	/	/	NMDCN0002PHA	2016	2016|GZ-18488|China	Sanger	1	V	5C1

32	/	/	/	KX225487	2013	2013|KX225487|China	Illumina	1	V	5C1
33	/	/	/	KX621249	2014	2014|KX621249|China	Illumina	1	V	5C1
34	/	/	/	KX459391	2014	2014|KX459391|China	Illumina	1	V	5C1
35	/	/	/	KX459389	2014	2014|KX459389|China	Illumina	1	V	5C1
36	/	/	/	KX459386	2014	2014|KX459386|China	Illumina	1	V	5C1
37	/	/	/	KX459387	2014	2014|KX459387|China	Illumina	1	V	5C1
38	/	/	/	KX459388	2014	2014|KX459388|China	Illumina	1	V	5C1
39	/	/	/	KX620451	2014	2014|KX620451|China	Illumina	1	V	5C1
40	/	/	/	KX620453	2014	2014|KX620453|China	Illumina	1	V	5C1
41	/	/	/	KX459390	2014	2014|KX459390|China	Illumina	1	V	5C1
42	/	/	/	KX459392	2014	2014|KX459392|China	Illumina	1	V	5C1
43	SD	Male	26	MN869904	2018	2018|MN869904|China	Illumina	1	I	1L1
44	SD	Female	61	MN869905	2018	2018|MN869905|China	Illumina	1	I	1J7
45	SD	Male	20	MN869906	2018	2018|MN869906|China	Illumina	1	I	1L1
46	DF	Male	67	MN869907	2018	2018|MN869907|China	Illumina	1	I	1L1
47	DF	Male	68	MN869908	2018	2018|MN869908|China	Illumina	1	I	1L1
48	DF	Female	79	MN869909	2018	2018|MN869909|China	Illumina	1	I	1L1
49	DF	Female	68	MN869910	2018	2018|MN869910|China	Illumina	1	I	1L1
50	DF	Male	32	MN869911	2018	2018|MN869911|China	Illumina	1	I	1L1
51	DF	Female	49	MN869912	2018	2018|MN869912|China	Illumina	1	I	1L1
52	DF	Male	23	MN869913	2018	2018|MN869913|China	Illumina	1	I	1L1
53	DF	Male	42	MN869914	2018	2018|MN869914|China	Illumina	1	I	1L1
54	SD	Male	82	MN886843	2014	2014|MN886843|China	Illumina	1	V	5C1
55	SD	Male	41	MN886880	2014	2014|MN886880|China	Illumina	1	I	1L2
56	DF	Female	56	MN886881	2014	2014|MN886881|China	Illumina	1	I	1K1
57	SD	Female	34	MN886844	2014	2014|MN886844|China	Illumina	1	V	5C1
58	DF	Male	64	MN886882	2014	2014|MN886882|China	Illumina	1	I	1L2
59	SD	Male	80	MN886845	2014	2014|MN886845|China	Illumina	1	V	5C1
60	SD	Female	64	MN886883	2014	2014|MN886883|China	Illumina	1	I	1L2
61	SD	Female	72	MN886846	2014	2014|MN886846|China	Illumina	1	V	5C1
62	DF	Female	62	MN886847	2014	2014|MN886847|China	Illumina	1	V	5C1
63	DF	Male	32	MN886848	2014	2014|MN886848|China	Illumina	1	V	5C1
64	SD	Female	84	MN886849	2014	2014|MN886849|China	Illumina	1	V	5C1
65	DF	Female	66	MN886884	2014	2014|MN886884|China	Illumina	1	I	1K1
66	DF	Male	66	MN886850	2014	2014|MN886850|China	Illumina	1	V	5C1
67	DF	Female	57	MN886851	2014	2014|MN886851|China	Illumina	1	V	5C1
68	DF	Female	50	MN886885	2014	2014|MN886885|China	Illumina	1	I	1L2
69	SD	Male	74	MN886852	2014	2014|MN886852|China	Illumina	1	V	5C1
70	SD	Female	29	MN886853	2014	2014|MN886853|China	Illumina	1	V	5C1
71	DF	Male	72	MN886854	2014	2014|MN886854|China	Illumina	1	V	5C1
72	DF	Male	65	MN886886	2014	2014|MN886886|China	Illumina	1	I	1K1
73	DF	Male	63	MN886887	2014	2014|MN886887|China	Illumina	1	I	1K1
74	DF	Female	48	MN886879	2014	2014|MN886879|China	Illumina	1	I	1K1
75	DF	Female	64	MN886855	2014	2014|MN886855|China	Illumina	1	V	5C1
76	SD	Female	36	MN886888	2014	2014|MN886888|China	Illumina	1	I	1K1
77	SD	Female	44	MN886856	2014	2014|MN886856|China	Illumina	1	V	5C1

78	SD	Male	27	MN886857	2014	2014|MN886857|China	Illumina	1	V	5C1
79	SD	Male	74	MN886858	2014	2014|MN886858|China	Illumina	1	V	5C1
80	SD	Female	58	MN886859	2014	2014|MN886859|China	Illumina	1	V	5C1
81	DF	Female	65	MN886860	2014	2014|MN886860|China	Illumina	1	V	5C1
82	DF	Female	63	MN886861	2014	2014|MN886861|China	Illumina	1	V	5C1
83	DF	Male	37	MN886889	2014	2014|MN886889|China	Illumina	1	I	1K1
84	DF	Male	76	MN886890	2014	2014|MN886890|China	Illumina	1	I	1K1
85	SD	Female	17	MN886862	2014	2014|MN886862|China	Illumina	1	V	5C1
86	DF	Female	64	MN886863	2014	2014|MN886863|China	Illumina	1	V	5C1
87	DF	Female	25	MN886891	2014	2014|MN886891|China	Illumina	1	I	1K1
88	SD	Female	65	MN886864	2014	2014|MN886864|China	Illumina	1	V	5C1
89	SD	Female	33	MN886892	2014	2014|MN886892|China	Illumina	1	I	1K1
90	DF	Male	73	MN886900	2013	2013|MN886900|China	Illumina	1	I	1K1
91	DF	Male	87	MN886865	2014	2014|MN886865|China	Illumina	1	V	5C1
92	DF	Male	73	MN886866	2014	2014|MN886866|China	Illumina	1	V	5C1
93	DF	Female	36	MN886867	2014	2014|MN886867|China	Illumina	1	V	5C1
94	DF	Female	48	MN886901	2013	2013|MN886901|China	Illumina	1	I	1K1
95	DF	Female	26	MN886902	2013	2013|MN886902|China	Illumina	1	I	1K1
96	SD	Female	72	MN886903	2013	2013|MN886903|China	Illumina	1	I	1K1
97	DF	Male	73	MN886904	2013	2013|MN886904|China	Illumina	1	I	1K1
98	SD	Male	40	MN886905	2013	2013|MN886905|China	Illumina	1	I	1K1
99	SD	Female	41	MN886893	2014	2014|MN886893|China	Illumina	1	I	1K1
100	SD	Male	73	MN886868	2014	2014|MN886868|China	Illumina	1	V	5C1
101	SD	Male	76	MN886869	2014	2014|MN886869|China	Illumina	1	V	5C1
102	DF	Female	54	MN886870	2014	2014|MN886870|China	Illumina	1	V	5C1
103	SD	Female	45	MN886906	2013	2013|MN886906|China	Illumina	1	I	1L2
104	SD	Female	63	MN886894	2014	2014|MN886894|China	Illumina	1	I	1K1
105	DF	Male	46	MN886871	2014	2014|MN886871|China	Illumina	1	V	5C1
106	SD	Female	88	MN886872	2014	2014|MN886872|China	Illumina	1	V	5C1
107	DF	Male	32	MN886895	2014	2014|MN886895|China	Illumina	1	I	1K1
108	DF	Female	27	MN886896	2014	2014|MN886896|China	Illumina	1	I	1K1
109	SD	Female	59	MN886873	2014	2014|MN886873|China	Illumina	1	V	5C1
110	DF	Female	25	MN886874	2014	2014|MN886874|China	Illumina	1	V	5C1
111	DF	Male	51	MN886907	2013	2013|MN886907|China	Illumina	1	I	1K1
112	SD	Female	90	MN886875	2014	2014|MN886875|China	Illumina	1	V	5C1
113	DF	Male	60	MN886876	2014	2014|MN886876|China	Illumina	1	V	5C1
114	DF	Female	34	MN886908	2013	2013|MN886908|China	Illumina	1	V	5C1
115	DF	Female	37	MN886909	2013	2013|MN886909|China	Illumina	1	I	1K1
116	DF	Female	57	MN886910	2013	2013|MN886910|China	Illumina	1	I	1K1
117	DF	Male	39	MN886911	2013	2013|MN886911|China	Illumina	1	I	1K1
118	DF	Male	28	MN886912	2013	2013|MN886912|China	Illumina	1	I	1K1
119	DF	Female	27	MN886877	2014	2014|MN886877|China	Illumina	1	V	5C1
120	DF	Male	35	MN886824	2017	2017|MN886824|China	Illumina	1	I	1L1
121	SD	Female	70	MN886825	2017	2017|MN886825|China	Illumina	1	I	1L1
122	SD	Female	63	MN886826	2017	2017|MN886826|China	Illumina	1	I	1L1
123	SD	Male	76	MN886827	2017	2017|MN886827|China	Illumina	1	I	1L1

124	DF	Male	28	MN886828	2017	2017|MN886828|China	Illumina	1	I	1L1
125	DF	Male	76	MN886878	2014	2014|MN886878|China	Illumina	1	V	5C1
126	DF	Female	44	MN886829	2017	2017|MN886829|China	Illumina	1	I	1L1
127	DF	Male	67	MN886830	2017	2017|MN886830|China	Illumina	1	I	1L1
128	DF	Female	79	MN886831	2017	2017|MN886831|China	Illumina	1	I	1L1
129	SD	Female	84	MN886832	2017	2017|MN886832|China	Illumina	1	I	1L1
130	DF	Female	80	MN886833	2017	2017|MN886833|China	Illumina	1	I	1L1
131	DF	Female	71	MN886834	2017	2017|MN886834|China	Illumina	1	I	1L1
132	SD	Female	67	MN886835	2017	2017|MN886835|China	Illumina	1	I	1L1
133	SD	Male	70	MN886836	2017	2017|MN886836|China	Illumina	1	I	1L1
134	DF	Male	71	MN886837	2017	2017|MN886837|China	Illumina	1	I	1L1
135	DF	Male	90	MN886838	2017	2017|MN886838|China	Illumina	1	I	1L1
136	SD	Male	30	MN886839	2017	2017|MN886839|China	Illumina	1	I	1E1
137	DF	Male	34	MN886913	2013	2013|MN886913|China	Illumina	1	I	1K1
138	DF	Female	74	MN886840	2017	2017|MN886840|China	Illumina	1	I	1L1
139	DF	Male	66	MN886841	2017	2017|MN886841|China	Illumina	1	I	1L1
140	SD	Female	55	MN886897	2014	2014|MN886897|China	Illumina	1	I	1K1
141	SD	Female	70	MN886842	2017	2017|MN886842|China	Illumina	1	I	1L1
142	DF	Male	63	MN886898	2014	2014|MN886898|China	Illumina	1	I	1K1
143	DF	Male	22	MN886899	2014	2014|MN886899|China	Illumina	1	I	1K1
144	DF	Female	35	MN886915	2006	2006|MN886915|China	Illumina	1	I	1H4
145	DF	Male	42	MN886916	2006	2006|MN886916|China	Illumina	1	I	1E1
146	DF	Male	41	MN886917	2006	2006|MN886917|China	Illumina	1	I	1E1
147	SD	Female	70	MN886914	2013	2013|MN886914|China	Illumina	1	I	1H4
148	DF	Male	56	MN886918	2006	2006|MN886918|China	Illumina	1	I	1E1
149	DF	Female	44	MN886919	2006	2006|MN886919|China	Illumina	1	I	1E1
150	DF	Female	47	MN886920	2006	2006|MN886920|China	Illumina	1	I	1E1
151	DF	Male	43	MN886921	2006	2006|MN886921|China	Illumina	1	I	1E1
152	DF	Male	24	MN886922	2006	2006|MN886922|China	Illumina	1	I	1E1
153	DF	Female	22	MN886923	2006	2006|MN886923|China	Illumina	1	I	1E1
154	DF	Female	32	MN886924	2006	2006|MN886924|China	Illumina	1	I	1H4
155	DF	Male	56	MW261824	2019	2019|MW261824|China	Illumina	1	I	1L1
156	DF	Male	20	MW261844	2019	2019|MW261844|China	Illumina	1	I	1E1
157	DF	Male	22	MW261845	2019	2019|MW261845|China	Illumina	1	I	1E1
158	DF	Female	56	MW261825	2019	2019|MW261825|China	Illumina	1	I	1L1
159	DF	Male	86	MW261826	2019	2019|MW261826|China	Illumina	1	I	1L1
160	DF	Male	23	MW261846	2019	2019|MW261846|China	Illumina	1	I	1E1
161	DF	Female	73	MW261827	2019	2019|MW261827|China	Illumina	1	I	1L1
162	DF	Male	49	MW261828	2019	2019|MW261828|China	Illumina	1	I	1L1
163	DF	Male	30	MW261847	2019	2019|MW261847|China	Illumina	1	I	1E1
164	DF	Male	62	MW261829	2019	2019|MW261829|China	Illumina	1	I	1E1
165	DF	Male	45	MW261830	2019	2019|MW261830|China	Illumina	1	I	1E1
166	DF	Male	50	MW261831	2019	2019|MW261831|China	Illumina	1	I	1E1
167	DF	Female	37	MW261832	2019	2019|MW261832|China	Illumina	1	V	5C1
168	DF	Male	27	MW261833	2019	2019|MW261833|China	Illumina	1	I	1E1
169	DF	Male	56	MW261834	2019	2019|MW261834|China	Illumina	1	I	1E1

170	DF	Female	46	MW261835	2019	2019|MW261835|China	Illumina	1	I	1E1
171	DF	Male	34	MW261836	2019	2019|MW261836|China	Illumina	1	IV	4C3
172	DF	Male	31	MW261837	2019	2019|MW261837|China	Illumina	1	I	1L1
173	SD	Male	44	MW261838	2019	2019|MW261838|China	Illumina	1	I	1E1
174	SD	Female	86	MW261839	2019	2019|MW261839|China	Illumina	1	I	1E1
175	SD	Male	40	MW261848	2019	2019|MW261848|China	Illumina	1	I	1E1
176	SD	Male	60	MW261840	2019	2019|MW261840|China	Illumina	1	I	1J7
177	DF	Male	62	MW261841	2019	2019|MW261841|China	Illumina	1	I	1E1
178	DF	Male	37	MW261842	2019	2019|MW261842|China	Illumina	1	I	1E1
179	DF	Male	20	MW261843	2019	2019|MW261843|China	Illumina	1	I	1E1

* DF = Dengue fever, SD = Severe dengue

** Data is deposited in China National Microbiology Data Center (NMDC, https://nmdc.cn/resource/genomics/sequence/) and National Center for Biotechnology Information (NCBI, https://www.ncbi.nlm.nih.gov/) with accession numbers.
