# Supplementary figures and images for: Phylodynamics unveils invading and diffusing patterns of dengue virus serotype-1 in Guangdong, China from 1990 to 2019 under a global genotyping framework
Source: Infect Dis Poverty. 2024 Jun 11;13:43. doi: 10.1186/s40249-024-01211-6 (PMC11165891; doi:10.1186/s40249-024-01211-6)

**a**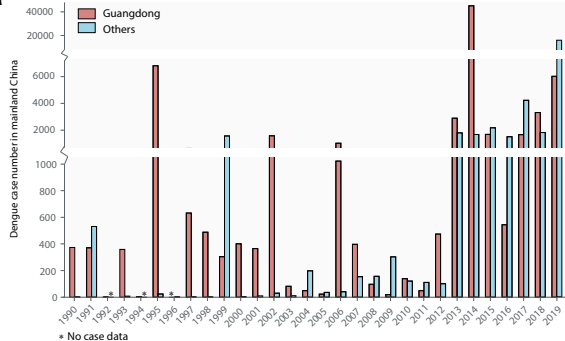**b**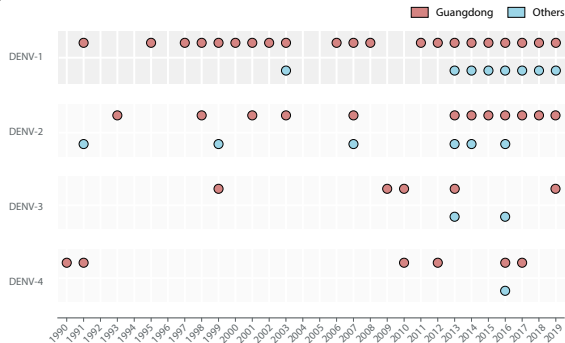**c**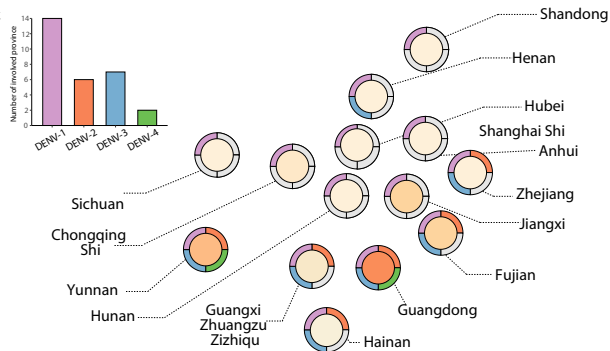**d**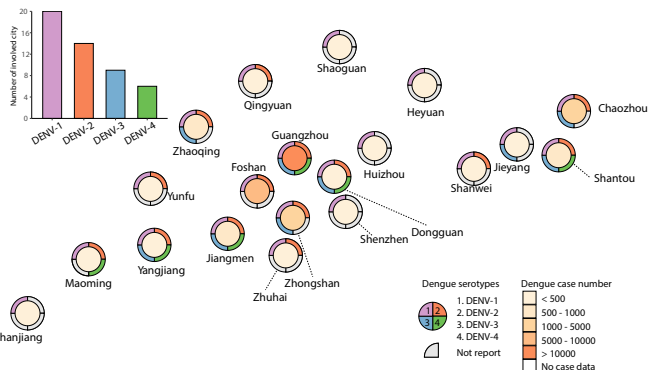

Supplement: Supplementary file 3 — Additional file 3: Figure S1. Epidemiological characteristics of DENVs in Chinese mainland during 1990‒2019. a, Dengue cases number reported in Guangdong (red) and other province (blue) in Chinese mainland during 1990‒2019. b, Serotypes of indigenous dengue reported in Guangdong (red) and other provinces (blue) in Chinese mainland during 1990‒2019. Serotypes data were obtained from local authorities archived in Guangdong Provincial Center for Diseases Control and Guangzhou Center for Diseases Control. Serotype identification is accomplished through serum testing techniques or molecular identification methods employed by local authorities. c, Epidemic provinces with indigenous dengue cases in Chinese mainland during 1990‒2019. The left bar shows the number of involved provinces of 4 serotypes of DENV, respectively. d, Epidemic cities with indigenous dengue cases in Guangdong Province during 1990‒2019. The left bar shows the number of involved cities in Guangdong Province circulating 4 serotypes of DENV, respectively. The color scale in inner circle core represents case number. Red, green, blue, and pink quarter pies represent 4 serotypes of DENV, respectively. [file 40249_2024_1211_MOESM3_ESM.pdf]

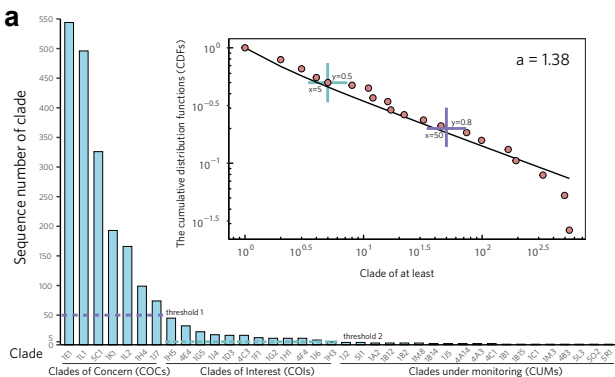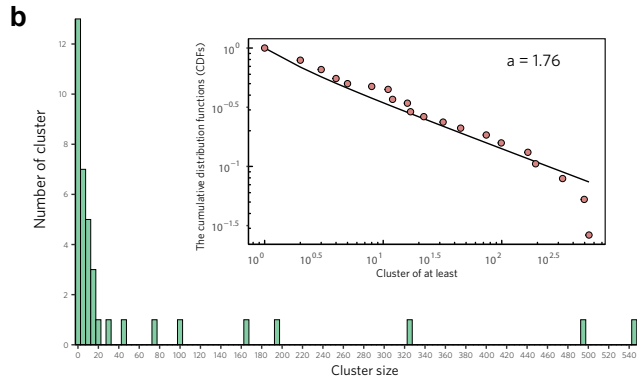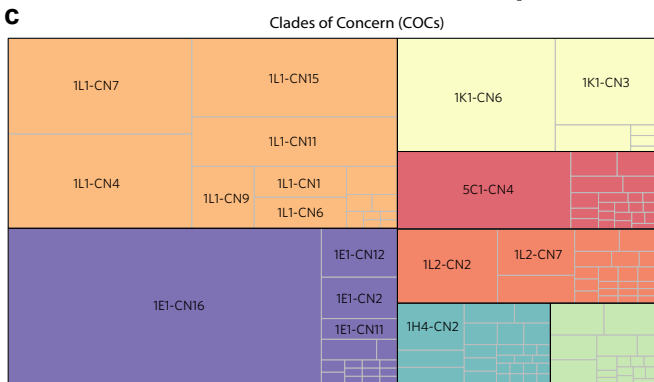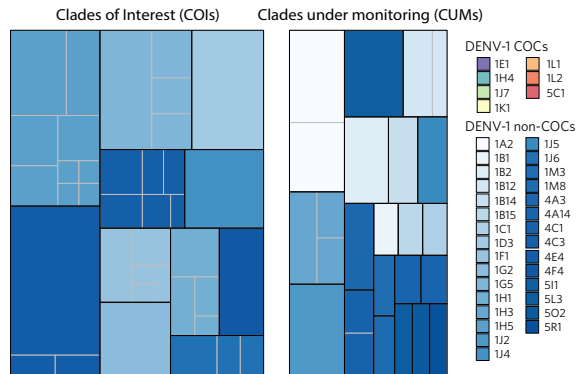

Supplement: Supplementary file 4 — Additional file 4: Figure S2. Distribution of the sequence number in the designated clades and clusters circulated in Chinese mainland. a, Blue bars show the sequence number in each clade. The inset shows the corresponding cumulative distribution functions (CDFs) of clade sequence number on double logarithmic axes. Values show coefficients of power-law distributions fitted to the sequence number in clades. Clades of Concern (COCs), Clades of Interest (COIs), and Clades under monitoring (CUMs) were classified based on the thresholds of 50 and 5 sequences, corresponding the respective cumulative 80% and 50% sequences. b, Green bars and inset show the sequence number in each designated transmission cluster and the corresponding CDFs of cluster sequence number on double logarithmic axes. c, Partition of the designated clusters sized by their sequence numbers. [file 40249_2024_1211_MOESM4_ESM.pdf]

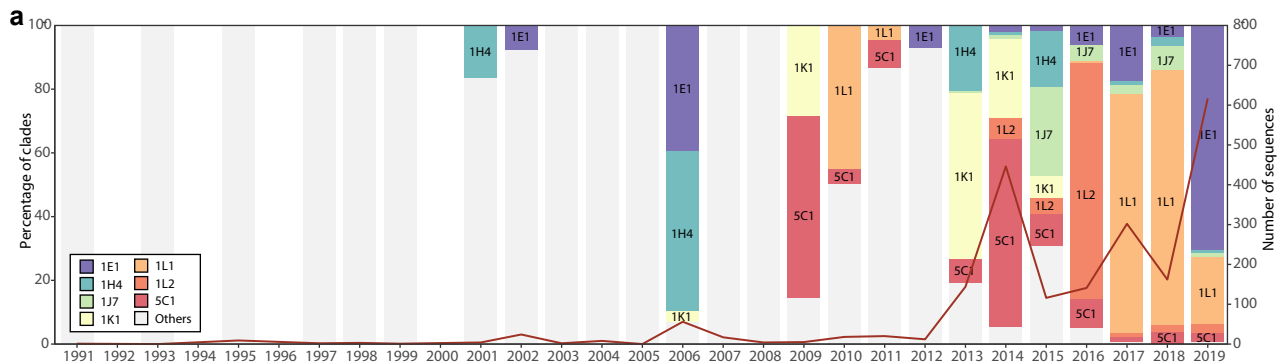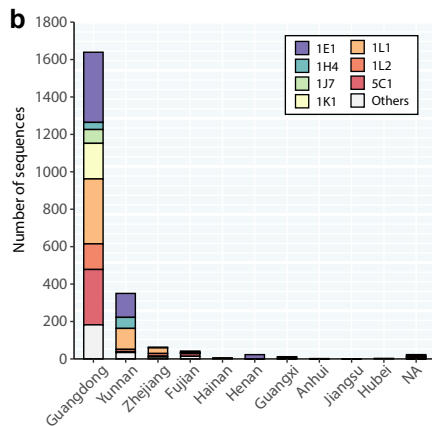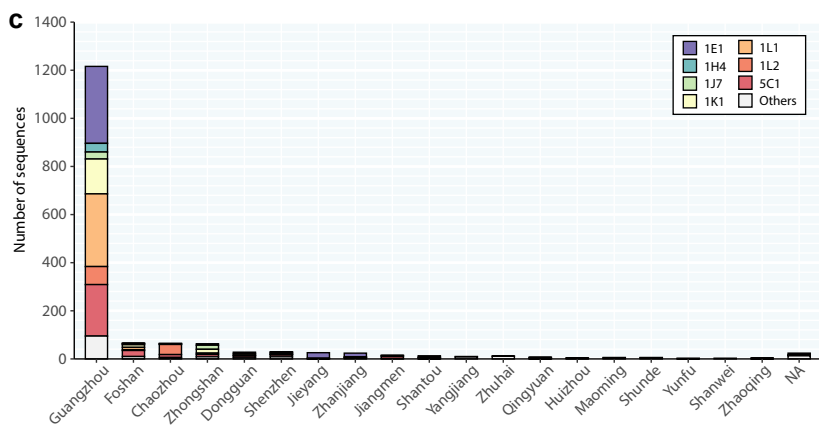

Supplement: Supplementary file 7 — Additional file 7: Figure S3. The composition and distribution of seven COCs of DENV-1 in Chinese mainland. Stacked barplots of proportion are colored by seven COCs along time from 1990 to 2019 (a). Stacked barplots of sequence number are colored by seven COCs showing their distribution in provinces in Chinese mainland (b) and cities in Guangdong Province (c). [file 40249_2024_1211_MOESM7_ESM.pdf]

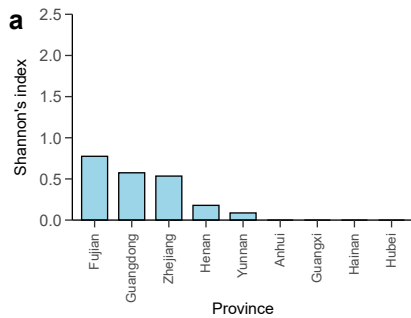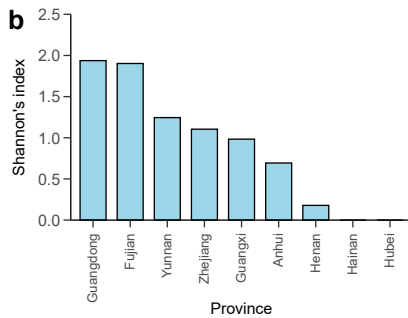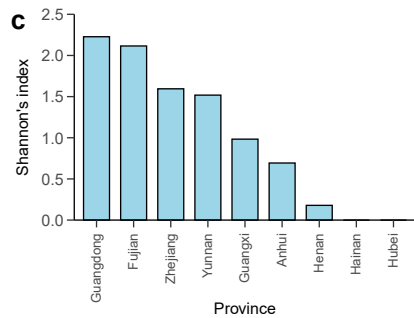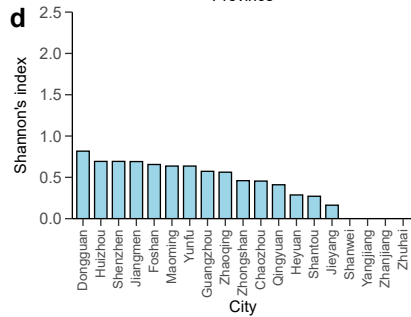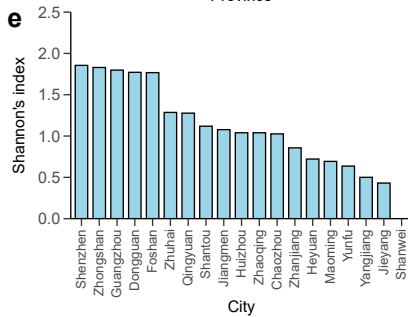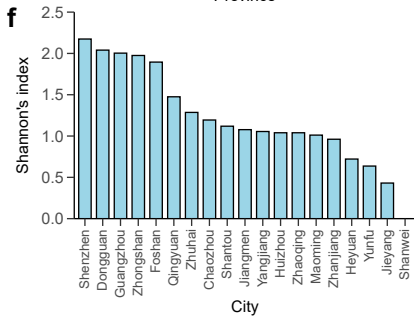

Supplement: Supplementary file 8 — Additional file 8: Figure S4. Shannon’s index of genotype (a, d), subgenotype (b, e), and clade (c, f) of DENV-1 circulated in each province in Chinese mainland (a-c) / city of Guangdong Province (d-f). [file 40249_2024_1211_MOESM8_ESM.pdf]

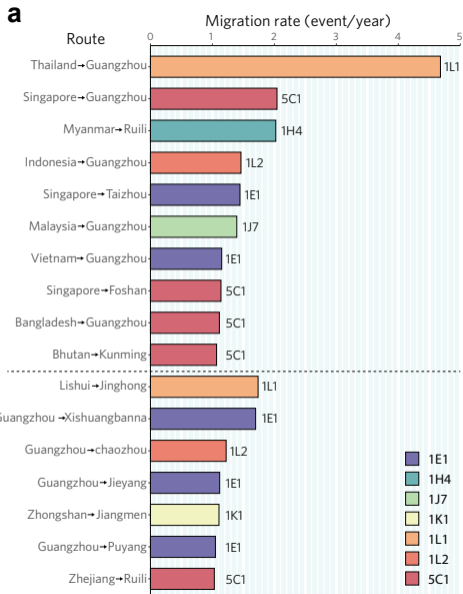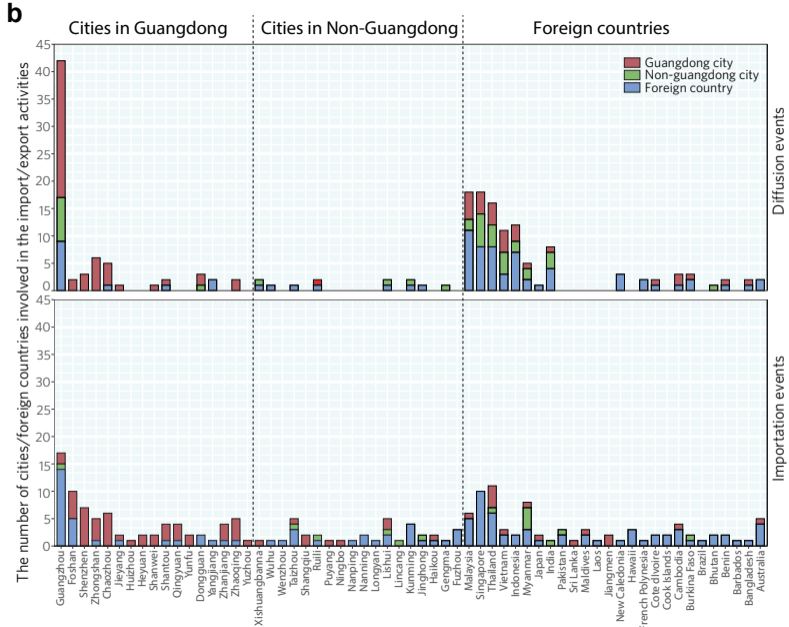

Supplement: Supplementary file 12 — Additional file 12: Figure S7. Summarizing the migration events of seven COCs of DENV-1 inferred by BSSVS. a. The estimated migration routes of seven COCs with migration rate > 1.0 events/year. b. Involved countries or cities number of the estimated importation and diffusion routes of seven COCs. [file 40249_2024_1211_MOESM12_ESM.pdf]

**a**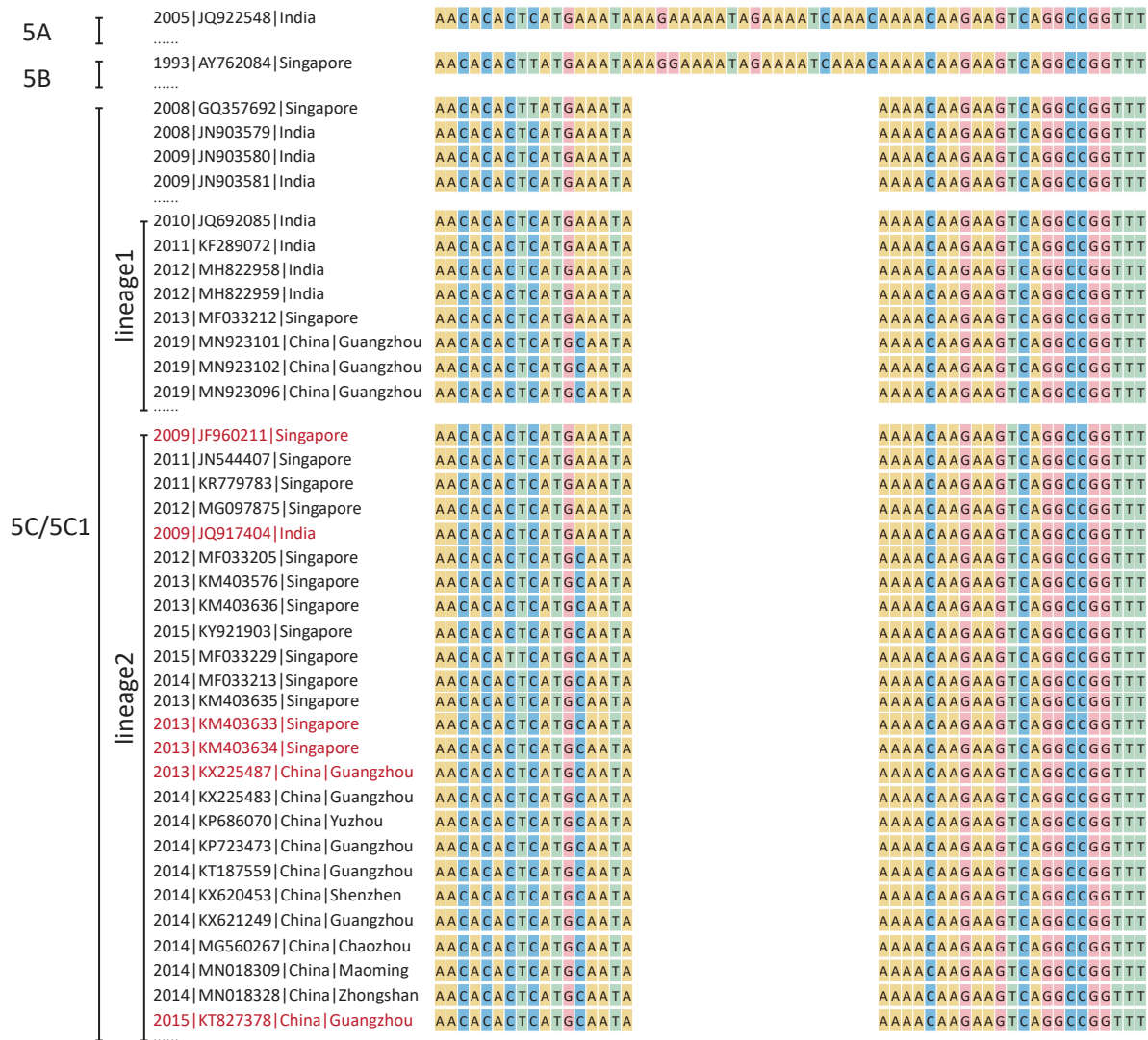**b**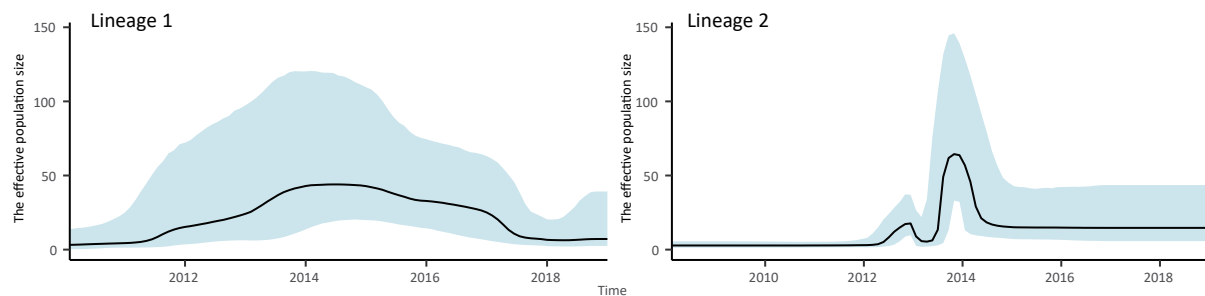

Supplement: Supplementary file 13 — Additional file 13: Figure S8. Sequence alignment and population size estimation of the COC 5C1. a. Multiple sequence alignment for 3’-UTR deletion region of 5C1. b. The dynamic description of the estimated population sizes for 5C1 Lineage 1 (left) and Lineage 2 (right). [file 40249_2024_1211_MOESM13_ESM.pdf]

**a**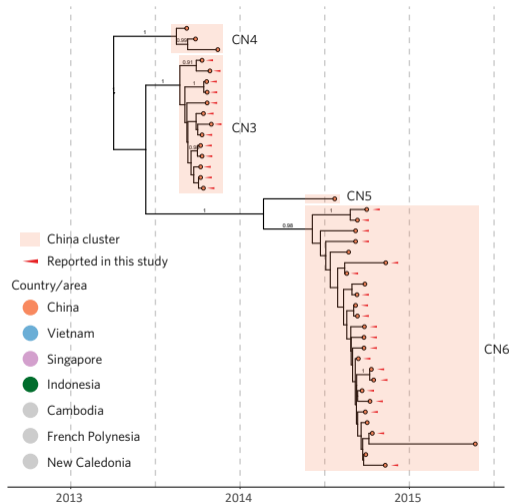**b**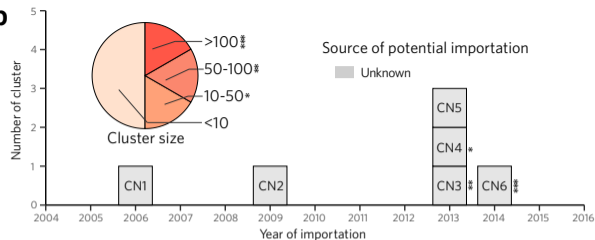**c**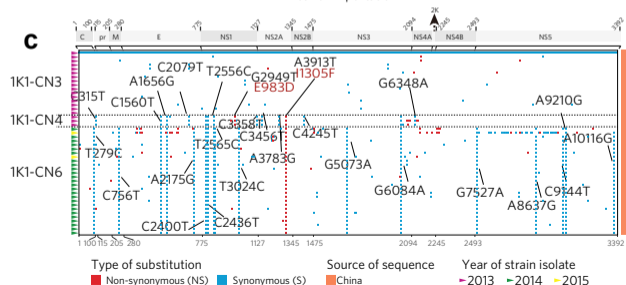

Supplement: Supplementary file 14 — Additional file 14: Figure S9. Invasion and diffusion of the COC 1K1 of DENV-1 in Chinese mainland. a. Maximum clade credibility phylogeny of the COC 1K1 based on the genomes. b. Temporal dynamics of the designated transmission clusters of 1K1 based on its importing source and year. The inserted pie chart shows the composition ratio of the size of the clusters of 1K1. c. Genome-wide SNPs analysis of the cluster 1K1-CN6. Only positions with specific SNPs found in the strains of cluster 1K1-CN6 isolated in Chinese mainland were shown, the SNPs of non-synonymous (NS) in blue and synonymous (S) in red. [file 40249_2024_1211_MOESM14_ESM.pdf]

**a**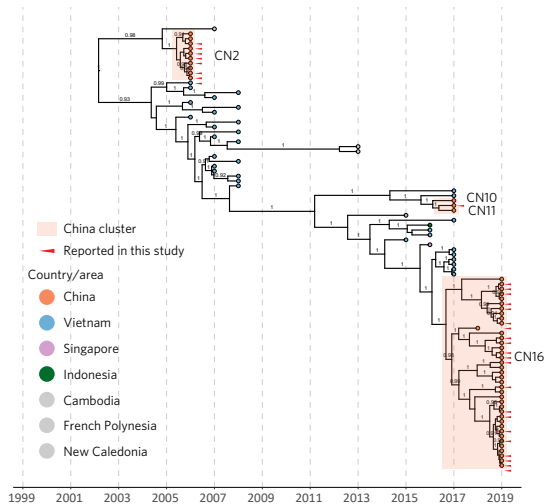**b**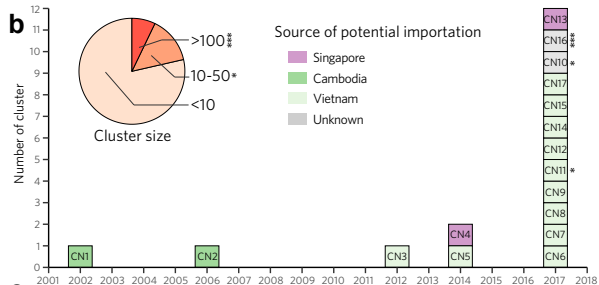**c**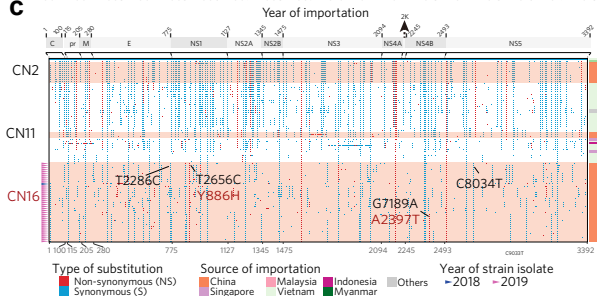

Supplement: Supplementary file 15 — Additional file 15: Figure S10. Invasion and diffusion of the COC 1E1 of DENV-1 in Chinese mainland. a. Maximum clade credibility phylogeny of the COC 1E1 based on the genomes. b. Temporal dynamics of the designated transmission clusters of 1E1 based on its importing source and year. The inserted pie chart shows the composition ratio of the size of the clusters of 1E1. c. Genome-wide SNPs analysis of the cluster 1E1-CN16. Only positions with specific SNPs found in the strains of cluster 1E1-CN16 isolated in Chinese mainland were shown, the SNPs of non-synonymous (NS) in blue and synonymous (S) in red. [file 40249_2024_1211_MOESM15_ESM.pdf]

**a**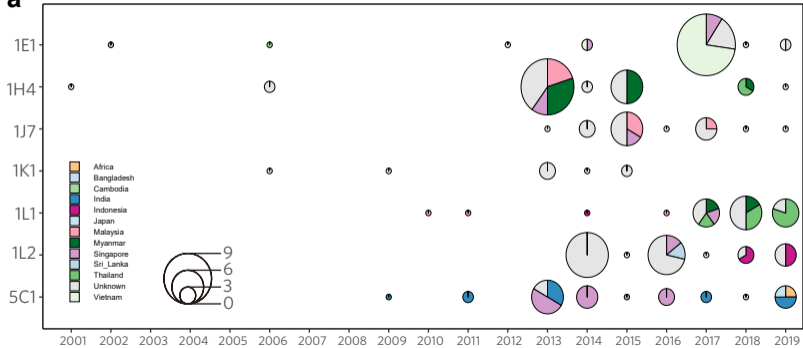**b**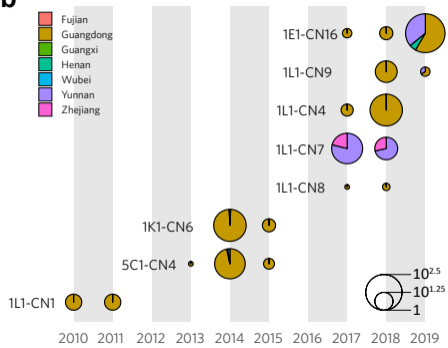

Supplement: Supplementary file 16 — Additional file 16: Figure S11. Characterizing the introduction sources and cross-year transmission of the seven COCs in Chinese mainland. a. Composition of introduction sources were inferred from the transmission clusters of the seven COCs from 2001 to2019. b. Schematic diagram of the transmission clusters of COCs with strains isolated in consecutive years from 2010 to 2019. The isolates number are indicated by pie chart size, and the provinces reported the isolates are showed by pie chart color. [file 40249_2024_1211_MOESM16_ESM.pdf]
