# Supplementary material for: Phylodynamics unveils invading and diffusing patterns of dengue virus serotype-1 in Guangdong, China from 1990 to 2019 under a global genotyping framework
Source: Infect Dis Poverty. 2024 Jun 11;13:43. doi: 10.1186/s40249-024-01211-6 (PMC11165891; doi:10.1186/s40249-024-01211-6)
Supplement: Supplementary file 5 — Additional file 5: Table S3. Summary of the Clades of Concern (COCs), Clades of Interest (COIs), and under monitoring (CUMs) of DENV-1 circulated in Chinese mainland. [file 40249_2024_1211_MOESM5_ESM.pdf]

Table S3. Summary of DENV-1 Clades of Concern (COCs), Clades of Interest (COIs), and Clades under monitoring (CUMs) in mainland China.

Group	No.	Clade	Sequence numbers	First report year	Outbreak	Location (Province)	Potential source
COCs	01	1E1	544	2002	2017, 2019	Fujian, Guangdong, Guangxi, Henan, Yunnan, Zhejiang	Vietnam, Myanmar
	02	1L1	496	2010	2017-2019	Fujian, Guangdong, Hainan, Yunnan, Zhejiang	Myanmar, Thailand
	03	5C1	326	2009	2013-2014, 2019	Anhui, Fujian, Guangdong, Guangxi, Henan, Hubei, Yunnan, Zhejiang	Singapore, India
	04	1K1	193	2006	2013-2014	Fujian, Guangdong, Guangxi	Unknown
	05	1L2	166	2014	2014	Fujian, Guangdong, Yunnan, Zhejiang	Singapore, Malaysia
	06	1H4	99	2001	2006,2013, 2015	Fujian, Guangdong, Yunnan	Singapore, Indonesia
	07	1J7	74	2013	2015	Guangdong	Singapore, Malaysia, Indonesia
COIs	08	1H5	45	2006	/	Fujian, Guangdong, Yunnan, Zhejiang	/
	09	4E4	32	1993	/	Guangdong	/
	10	1G5	22	2013	/	Guangdong, Guangxi, Yunnan	/
	11	1J4	17	2007	/	Guangdong	/
	12	1D3	16	2011	/	Guangdong	/
	13	4C3	16	2007	/	Guangdong, Zhejiang	/
	14	1F1	12	2011	/	Fujian, Guangdong	/
	15	1G2	11	2014	/	Guangdong, Yunnan	/
	16	1H1	11	2001	/	Guangdong, Zhejiang	/
	17	4F4	11	1995	/	Guangdong	/
	18	1J6	8	2004	/	Fujian, Guangdong	/
	19	1H3	5	2010	/	Fujian, Guangdong, Yunnan	/
CUMs	20	1J2	4	2012	/	Guangdong	/
	21	5I1	4	2013	/	Fujian, Zhejiang	/

	22	1A2	3	1985	/	Guangdong	/
	23	1B12	3	1998	/	Guangdong	/
	24	1B2	3	1997	/	Guangdong	/
	25	1M8	3	2016	/	Guangdong	/
	26	1B14	2	1998	/	Guangdong	/
	27	1J5	2	2008	/	Guangdong	/
	28	4A14	2	2013	/	Fujian, Guangdong	/
	29	4A3	2	1991	/	Guangdong	/
	30	4C1	2	2010	/	Guangdong, Jiangsu	/
	31	1B1	1	1991	/	Guangdong	/
	32	1B15	1	1997	/	Guangdong	/
	33	1C1	1	2008	/	Guangdong	/
	34	1M3	1	2009	/	Guangdong	/
	35	4B3	1	2010	/	Guangdong	/
	36	5L3	1	2015	/	Guangdong	/
	37	5O2	1	2013	/	Zhejiang	/
	38	5R1	1	2016	/	Anhui	/

COCs = Clades of Concern, COIs = Clades of Interest, CUMs = Clades under monitoring
