# Supplementary material for: Phylodynamics unveils invading and diffusing patterns of dengue virus serotype-1 in Guangdong, China from 1990 to 2019 under a global genotyping framework
Source: Infect Dis Poverty. 2024 Jun 11;13:43. doi: 10.1186/s40249-024-01211-6 (PMC11165891; doi:10.1186/s40249-024-01211-6)
Supplement: Supplementary file 6 — Additional file 6: Table S4. Characterizing the spatio-temporal distribution and inferring the importing sources of the transmission clusters epidemic in Chinese mainland. [file 40249_2024_1211_MOESM6_ESM.pdf]

Table S4. Spatio-temporal distribution characterizations and import source of the transmission lineages (TL) epidemic in mainland China.

No.	Clade	TL	Year	First report year	Province	Sequence number	City number	Import source	Import source support level
1	1E1	CN1	2002	2002	Guangdong	2	1	Cambodia	Es
2	1E1	CN2	2006	2006	Guangdong	22	1	Cambodia	Es
3	1E1	CN3	2012	2012	Fujian	1	1	Vietnam	Ps
4	1E1	CN4	2014	2014	Guangxi	8	1	Singapore	Ps
5	1E1	CN5	2014	2014	Fujian	1	1	Vietnam	Ps
6	1E1	CN6	2017	2017	Guangdong	1	1	Vietnam	Ss
7	1E1	CN7	2017	2017	Zhejiang	1	1	Vietnam	Ps
8	1E1	CN8	2017	2017	Fujian	3	1	Vietnam	Ps
9	1E1	CN9	2017	2017	Guangdong	1	1	Vietnam	Ps
10	1E1	CN10	2017	2017	Guangdong	1	1	Unknown	El
11	1E1	CN11	2017	2017	Guangdong	11	2	Vietnam	Ps
12	1E1	CN12	2017	2017	Guangdong	26	2	Vietnam	Ps
13	1E1	CN13	2017	2017	Zhejiang	1	1	Singapore	Ss
14	1E1	CN14	2017	2017	Zhejiang	1	1	Vietnam	Ss
15	1E1	CN15	2017	2017	Guangdong	2	2	Vietnam	Ss
16	1E1	CN16	2017-2019	2017	Guangdong, Yunnan, Henan	343	5	Unknown	El
17	1E1	CN17	2019	2019	Guangdong	1	1	Vietnam	Es
18	1H4	CN1	2001	2001	Guangdong	1	1	Unknown	El
19	1H4	CN2	2006	2006	Guangdong	22	2	Unknown	El
20	1H4	CN3	2006	2006	Guangdong	7	1	Unknown	El
21	1H4	CN4	2013	2013	Yunnan	4	1	Myanmar	Ss
22	1H4	CN5	2013	2013	Fujian	1	1	Malaysia	Ps
23	1H4	CN6	2013	2013	Yunnan	3	1	Malaysia	Ps
24	1H4	CN7	2013	2013	Yunnan	8	1	Myanmar	Ps
25	1H4	CN8	2013	2013	Yunnan	1	1	Unknown	El
26	1H4	CN9	2013	2013	Guangdong	1	1	Singapore	Ps
27	1H4	CN10	2013	2013	Yunnan	5	1	Unknown	El
28	1H4	CN11	2013	2013	Yunnan	1	1	Unknown	El
29	1H4	CN12	2013	2013	Yunnan	1	1	Unknown	El
30	1H4	CN13	2013	2013	Yunnan	1	1	Myanmar	Ss
31	1H4	CN14	2014	2014	Yunnan	1	1	Unknown	El
32	1H4	CN15	2014	2014	Yunnan	4	1	Unknown	El
33	1H4	CN16	2015	2015	Yunnan	5	1	Unknown	El
34	1H4	CN17	2015	2015	Yunnan	1	1	Unknown	El
35	1H4	CN18	2015	2015	Yunnan	1	1	Unknown	El
36	1H4	CN19	2015	2015	Yunnan	1	1	Myanmar	Ss
37	1H4	CN20	2015	2015	Yunnan	8	2	Myanmar	Ss
38	1H4	CN21	2015	2015	Yunnan	2	1	Myanmar	Ps
39	1H4	CN22	2018	2018	Guangdong	1	1	Myanmar	Ps
40	1H4	CN23	2018	2018	Yunnan	1	1	Thailand	Ps
41	1H4	CN24	2018	2018	Yunnan	3	1	Thailand	Ps

42	1H4	CN25	2019	2019	Guangdong	3	1	Unknown	El
43	1J7	CN1	2013	2013	Guangdong	1	1	Unknown	El
44	1J7	CN2	2014	2014	Guangdong	3	1	Unknown	El
45	1J7	CN3	2014	2014	Guangdong	1	1	Unknown	El
46	1J7	CN4	2014	2014	Guangdong	1	1	Unknown	El
47	1J7	CN5	2015	2015	Guangdong	6	1	Unknown	El
48	1J7	CN6	2015	2015	Guangdong	1	1	Unknown	El
49	1J7	CN7	2015	2015	Guangdong	2	1	Unknown	El
50	1J7	CN8	2015	2015	Guangdong	12	1	Malaysia	Ps
51	1J7	CN9	2015	2015	Guangdong	1	1	Malaysia	Ps
52	1J7	CN10	2015	2015	Guangdong	8	4	Singapore	Ss
53	1J7	CN11	2016	2016	Guangdong	1	1	Unknown	El
54	1J7	CN12	2017	2017	Guangdong	1	1	Unknown	Es
55	1J7	CN13	2017	2017	Guangdong	1	1	Unknown	Es
56	1J7	CN14	2017	2017	Guangdong	1	1	Unknown	Es
57	1J7	CN15	2017	2017	Guangdong	1	1	Malaysia	Ps
58	1J7	CN16	2015-2018	2015	Guangdong	12	5	Unknown	Es
59	1J7	CN17	2019	2019	Guangdong	8	1	Unknown	Es
60	1K1	CN1	2006	2006	Guangdong	2	2	Unknown	El
61	1K1	CN2	2009	2009	Guangdong	2	1	Unknown	El
62	1K1	CN3	2013-2015	2013	Guangdong	64	3	Unknown	El
63	1K1	CN4	2013	2013	Guangdong	14	3	Unknown	El
64	1K1	CN5	2013	2013	Guangdong	1	1	Unknown	El
65	1K1	CN6	2014-2015	2014	Guangdong, Fujian, Guangxi	125	11	Unknown	El
66	1L1	CN1	2010-2011	2010	Guangdong	20	3	Malaysia	Ps
67	1L1	CN2	2014	2014	Fujian	1	1	Indonesia	Es
68	1L1	CN3	2016	2016	Guangdong	1	1	Malaysia	Ss
69	1L1	CN4	2017-2018	2017	Guangdong	121	9	Unknown	El
70	1L1	CN5	2017	2017	Guangdong	2	1	Thailand	Es
71	1L1	CN6	2017	2017	Guangdong	20	1	Singapore	Ss
72	1L1	CN7	2017-2018	2017	Zhejiang, Yunnan	123	2	Myanmar	Ps
73	1L1	CN8	2017-2018	2017	Guangdong	3	1	Unknown	El
74	1L1	CN9	2018-2019	2018	Guangdong, Yunnan	27	1	Thailand	Ss
75	1L1	CN10	2018	2018	Guangdong	1	1	Unknown	El
76	1L1	CN11	2018	2018	Guangdong, Zhejiang	71	2	Thailand	Ss
77	1L1	CN12	2019	2019	Guangdong, Hainan	10	2	Thailand	Ss
78	1L1	CN13	2019	2019	Guangdong	3	1	Unknown	El
79	1L1	CN14	2019	2019	Guangdong	1	1	Thailand	Ss
80	1L1	CN15	2019	2019	Guangdong	113	1	Thailand	Ss
81	1L2	CN1	2014	2014	Guangdong	1	1	Unknown	El
82	1L2	CN2	2014,2016	2014	Guangdong	52	3	Unknown	El
83	1L2	CN3	2014	2014	Guangdong	7	2	Unknown	El
84	1L2	CN4	2014	2014	Guangdong	2	1	Unknown	El
85	1L2	CN5	2014	2014	Guangdong	1	1	Unknown	El

86	1L2	CN6	2014	2014	Guangdong	2	1	Unknown	El
87	1L2	CN7	2014	2014	Guangdong	25	1	Unknown	El
88	1L2	CN8	2014	2014	Guangdong	2	1	Unknown	El
89	1L2	CN9	2015	2015	Guangdong	1	1	Unknown	El
90	1L2	CN10	2016	2016	Guangdong	2	1	Unknown	El
91	1L2	CN11	2016	2016	Guangdong	2	1	Unknown	El
92	1L2	CN12	2016	2016	Guangdong	1	1	Sir Lanka	Ps
93	1L2	CN13	2016	2016	Zhejiang	3	1	Singapore	Ps
94	1L2	CN14	2016	2016	Guangdong	1	1	Unknown	El
95	1L2	CN15	2016	2016	Guangdong	15	3	Unknown	El
96	1L2	CN16	2017	2017	Yunnan	4	1	Unknown	El
97	1L2	CN17	2018	2018	Guangdong	1	1	Indonesia	Ss
98	1L2	CN18	2018	2018	Guangdong	1	1	Indonesia	Ss
99	1L2	CN19	2018	2018	Guangdong	2	1	Unknown	El
100	1L2	CN20	2019	2019	Guangdong	1	1	Indonesia	Ss
101	1L2	CN21	2019	2019	Zhejiang	1	1	Indonesia	Ss
102	1L2	CN22	2019	2019	Yunnan	2	1	Unknown	El
103	1L2	CN23	2019	2019	Yunnan	6	1	Unknown	El
104	5C1	CN1	2009	2009	Guangdong	1	1	India	Ps
105	5C1	CN2	2011	2011	Guangdong	1	1	India	Ps
106	5C1	CN3	2011	2011	Guangdong	2	1	India	Ps
107	5C1	CN4	2013-2015	2013	Guangdong, Henan, Hubei	94	12	Singapore	Ps
108	5C1	CN5	2013	2013	Guangdong	1	1	Singapore	Ps
109	5C1	CN6	2013	2013	Yunnan	3	1	Singapore	Ps
110	5C1	CN7	2013	2013	Zhejiang, Yunnan	2	2	India	Ps
111	5C1	CN8	2013	2013	Guangdong, Zhejiang	2	2	India	Ps
112	5C1	CN9	2013	2013	Guangdong	1	1	Unknown	El
113	5C1	CN10	2014	2014	Guangdong	1	1	Singapore	Ss
114	5C1	CN11	2014	2014	Guangdong	1	1	Singapore	Ss
115	5C1	CN12	2014	2014	Zhejiang, Guangdong	8	3	Singapore	Es
116	5C1	CN13	2016	2016	Anhui	1	1	Singapore	Ps
117	5C1	CN14	2016	2016	Guangdong	1	1	Singapore	Ss
118	5C1	CN15	2016	2016	Guangdong	6	1	Singapore	Es
119	5C1	CN16	2017	2017	Guangdong	1	1	India	Ss
120	5C1	CN17	2017	2017	Zhejiang	1	1	India	Ss
121	5C1	CN18	2018	2018	Guangdong	4	1	Bangladesh	Es
122	5C1	CN19	2019	2019	Guangdong	1	1	India	Ps
123	5C1	CN20	2019	2019	Guangdong	1	1	India	Ss
124	5C1	CN21	2019	2019	Guangdong	7	1	Japan	Ps
125	5C1	CN22	2019	2019	Guangdong	1	1	Africa	Es
126	5C1	CN23	2019	2019	Guangzhou	1	1	Bangladesh	Ss
127	1H5	CN1	2006	2006	Guangdong	4	1	Unknown	El
128	1H5	CN2	2013	2013	Zhejiang	1	1	Sri Lanka	Ps
129	1H5	CN3	2013	2013	Guangdong	1	1	Myanmar	Es
130	1H5	CN4	2013	2013	Guangdong	1	1	Thailand	Ps

131	1H5	CN5	2013	2013	Yunnan	6	1	Malaysia	Ps
132	1H5	CN6	2014	2014	Yunnan	10	1	Myanmar	Es
133	1H5	CN7	2015	2015	Fujian	4	1	Sri Lanka	Es
134	1H5	CN8	2015	2015	Yunnan	5	2	Myanmar	Es
135	1H5	CN9	2017	2017	Zhejiang	1	1	Unknown	El
136	4E4	CN1	1993	1993	Guangdong	2	1	Unknown	El
137	4E4	CN2	2002-2003	2002	Guangdong	28	1	Unknown	El
138	4E4	CN3	2004,2007	2004	Guangdong	1	2	Unknown	El
139	1G5	CN1	2013	2013	Guangdong	4	1	Thailand	Es
140	1G5	CN2	2014	2014	Guangdong, Guangxi	3	2	Unknown	El
141	1G5	CN3	2015	2015	Yunnan	3	1	Myanmar	Es
142	1G5	CN4	2015	2015	Guangdong	13	2	Unknown	El
143	1J4	CN1	2007	2007	Guangdong	13	4	Unknown	El
144	1D3	CN1	2011	2011	Guangdong	18	1	Vietnam	Ps
145	4C3	CN1	2007	2007	Guangdong	4	1	Unknown	El
146	4C3	CN2	2010	2010	Guangdong	3	1	Philippines	Ps
147	4C3	CN3	2013	2013	Zhejiang	2	1	Philippines	Es
148	4C3	CN4	2016	2016	Guangdong	2	1	Philippines	Ss
149	4C3	CN5	2017	2017	Guangdong	1	1	Philippines	Ss
150	4C3	CN6	2019	2019	Guangdong	2	1	Philippines	Ss
151	1F1	CN1	2011-2012	2011	Guangdong	3	1	Vietnam	Es
152	1F1	CN2	2012	2012	Guangdong	1	1	Cambodia	Es
153	1F1	CN3	2012	2012	Guangdong	1	1	Unknown	El
154	1F1	CN4	2012-2013	2012	Guangdong	5	2	Cambodia	Ps
155	1F1	CN5	2013	2013	Guangdong	1	1	Thailand	Es
156	1G2	CN1	2014	2014	Guangdong, Yunnan	11	2	Unknown	El
157	1H1	CN1	2001	2001	Guangdong	5	1	Thailand	Ps
158	1H1	CN2	2004	2004	Zhejiang	2	1	Thailand	Es
159	1H1	CN3	2008,2010	2008	Guangdong	3	1	Thailand	Ps
160	1H1	CN4	2010	2010	Guangdong	1	1	Thailand	Ps
161	4F4	CN1	1995	1995	Guangdong	10	3	Unknown	El
162	1J6	CN1	2004	2004	Guangdong	2	2	Singapore	Es
163	1J6	CN2	2004	2004	Fujian	4	1	Malaysia	Es
164	1J6	CN3	2005	2005	Guangdong	2	1	Singapore	Es
165	1H3	CN1	2010	2010	Guangdong	2	1	Laos	Ps
166	1H3	CN2	2010	2010	Fujian	1	1	Laos	Ss
167	1H3	CN3	2010	2010	Guangdong	1	1	Laos	Es
168	1J2	CN1	2012-2013	2012	Guangdong	4	1	Unknown	El
169	5I1	CN1	2013	2013	Zhejiang, Fujian	4	2	Angola	Es
170	1A2	CN1	1985	1985	Guangdong	3	1	Unknown	El
171	1A2	CN2	1998	1998	Guangdong	4	1	Thailand	Es
172	1B12	CN1	1998	1998	Guangdong	1	1	Thailand	Es
173	1B12	CN2	1999	1999	Guangdong	2	1	Thailand	Es
174	1B2	CN1	1997	1997	Guangdong	3	2	Unknown	El
175	1M8	CN1	2016	2016	Guangdong	1	1	Indonesia	Es

176	1B14	CN1	1998-1999	1998	Guangdong	2	1	Unknown	El
177	1J5	CN1	2008	2008	Guangdong	2	1	Thailand	Es
178	4A14	CN1	2013	2013	Fujian	1	1	Philippines	Es
179	4A3	CN1	1991,2010	1991	Guangdong	2	1	Unknown	El
180	4C1	CN1	2010	2010	Guangdong	1	1	Philippines	Es
181	4C1	CN2	2012	2012	Jiangsu	1	1	Philippines	Es
182	1B1	CN1	1991	1991	Guangdong	1	1	Thailand	Es
183	1B15	CN1	1997	1997	Guangdong	1	1	Unknown	El
184	1C1	CN1	2008	2008	Guangdong	1	1	Vietnam	Es
185	1M3	CN1	2009	2009	Guangdong	1	1	Indonesia	Es
186	4B3	CN1	2010	2010	Guangdong	1	1	Indonesia	Es
187	5L3	CN1	2015	2015	Guangdong	1	1	Brazil	Es
188	5O2	CN1	2013	2013	Zhejiang	1	1	USA	Es
189	5R1	CN1	2016	2016	Anhui	1	1	Brazil	Es

TL = Transmission lineage, Ss = Strong Supported, Es = Evidence Supported, Ps = Partially Supported, El = Evidence limited
