# Supplementary material for: Phylodynamics unveils invading and diffusing patterns of dengue virus serotype-1 in Guangdong, China from 1990 to 2019 under a global genotyping framework
Source: Infect Dis Poverty. 2024 Jun 11;13:43. doi: 10.1186/s40249-024-01211-6 (PMC11165891; doi:10.1186/s40249-024-01211-6)
Supplement: Supplementary file 10 — Additional file 10: Figure S6. phylogenetic trees of COCs 1E1 (a), 1H4 (b), 1J7 (c), 1K1 (d), 1L1 (e), 1L2 (f), and 5C1 (g) established under the E-gene-based global unified framework of DENV-1. The designated transmission clusters circulated in Chinese mainland are shaded in apricot. The colored circles indicated the locations of strains. [file 40249_2024_1211_MOESM10_ESM.pdf]

a 1E1

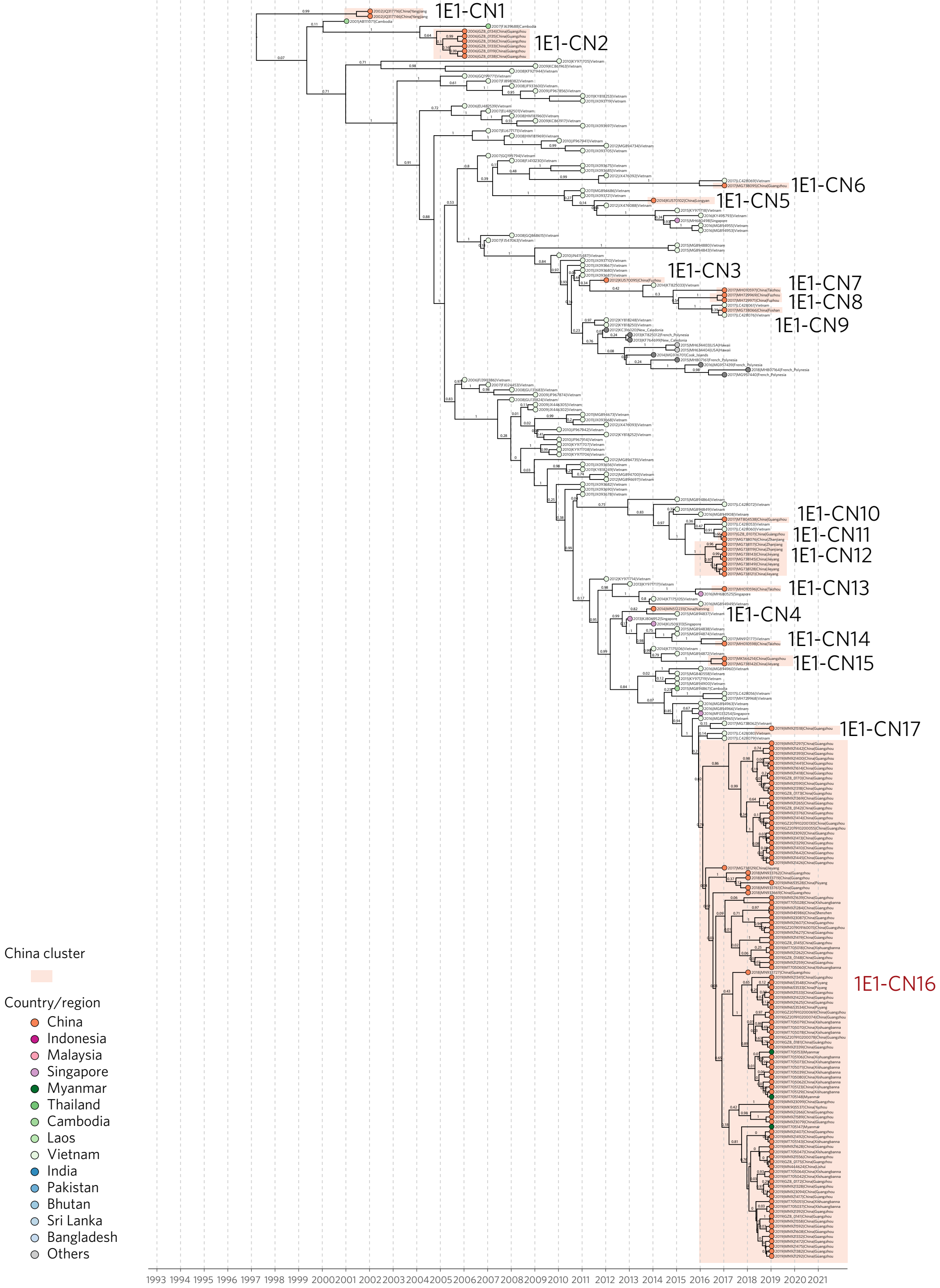
1E1-CN1
1E1-CN2
1E1-CN6
1E1-CN5
1E1-CN3
1E1-CN7
1E1-CN8
1E1-CN9
1E1-CN10
1E1-CN11
1E1-CN12
1E1-CN13
1E1-CN4
1E1-CN14
1E1-CN15
1E1-CN17
1E1-CN16
China cluster
Country/region
China
Indonesia
Malaysia
Singapore
Myanmar
Thailand
Cambodia
Laos
Vietnam
India
Pakistan
Bhutan
Sri Lanka
Bangladesh
Others
1993 1994 1995 1996 1997 1998 1999 2000 2001 2002 2003 2004 2005 2006 2007 2008 2009 2010 2011 2012 2013 2014 2015 2016 2017 2018 2019 2020 2021

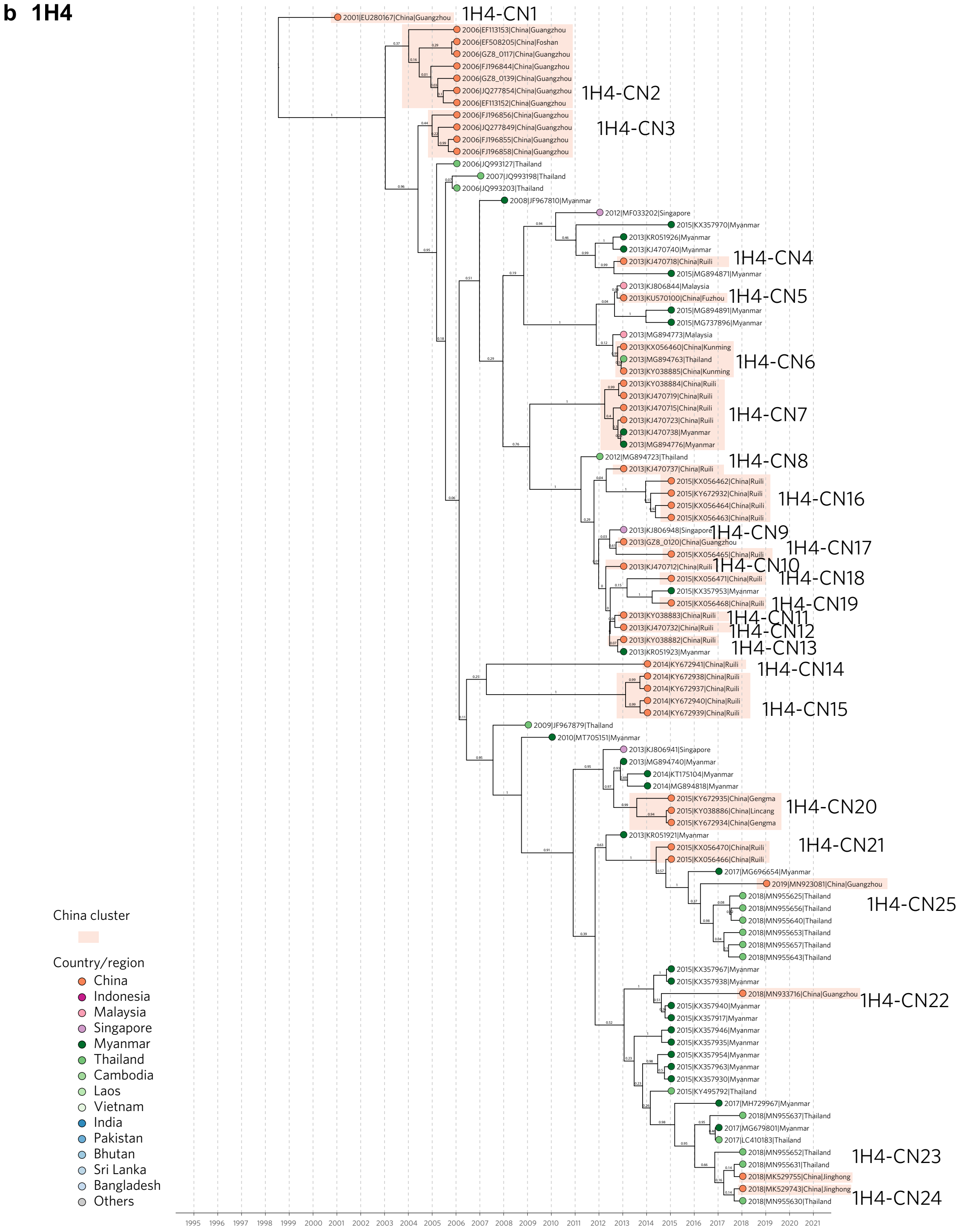
b 1H4
2001|EU280167|China|Guangzhou
1H4-CN1
2006|EF113153|China|Guangzhou
2006|EF508205|China|Foshan
2006|GZ8_0117|China|Guangzhou
2006|FJ196844|China|Guangzhou
2006|GZ8_0139|China|Guangzhou
2006|JQ277854|China|Guangzhou
2006|EF113152|China|Guangzhou
1H4-CN2
2006|FJ196856|China|Guangzhou
2006|JQ277849|China|Guangzhou
2006|FJ196855|China|Guangzhou
2006|FJ196858|China|Guangzhou
1H4-CN3
2006|JQ993127|Thailand
2007|JQ993198|Thailand
2006|JQ993203|Thailand
2008|JF967810|Myanmar
2012|MF033202|Singapore
2015|KX357970|Myanmar
2013|KR051926|Myanmar
2013|KJ470740|Myanmar
2013|KJ470718|China|Ruili
1H4-CN4
2015|MG894871|Myanmar
2013|KJ806844|Malaysia
2013|KU570100|China|Fuzhou
1H4-CN5
2015|MG894891|Myanmar
2015|MG737896|Myanmar
2013|MG894773|Malaysia
2013|KX056460|China|Kunming
2013|MG894763|Thailand
2013|KY038885|China|Kunming
1H4-CN6
2013|KY038884|China|Ruili
2013|KJ470719|China|Ruili
2013|KJ470715|China|Ruili
2013|KJ470723|China|Ruili
2013|KJ470738|Myanmar
2013|MG894776|Myanmar
1H4-CN7
2012|MG894723|Thailand
2013|KJ470737|China|Ruili
1H4-CN8
2015|KX056462|China|Ruili
2015|KY672932|China|Ruili
2015|KX056464|China|Ruili
2015|KX056463|China|Ruili
1H4-CN16
2013|KJ806948|Singapore
1H4-CN9
2013|GZ8_0120|China|Guangzhou
2015|KX056465|China|Ruili
1H4-CN17
2013|KJ470712|China|Ruili
1H4-CN10
2015|KX056471|China|Ruili
1H4-CN18
2015|KX357953|Myanmar
2015|KX056468|China|Ruili
1H4-CN19
2013|KY038883|China|Ruili
1H4-CN11
2013|KJ470732|China|Ruili
1H4-CN12
2013|KY038882|China|Ruili
2013|KR051923|Myanmar
1H4-CN13
2014|KY672941|China|Ruili
1H4-CN14
2014|KY672938|China|Ruili
2014|KY672937|China|Ruili
2014|KY672940|China|Ruili
2014|KY672939|China|Ruili
1H4-CN15
2009|JF967879|Thailand
2010|MT705151|Myanmar
2013|KJ806941|Singapore
2013|MG894740|Myanmar
2014|KT175104|Myanmar
2014|MG894818|Myanmar
2015|KY672935|China|Gengma
2015|KY038886|China|Lincang
2015|KY672934|China|Gengma
1H4-CN20
2013|KR051921|Myanmar
2015|KX056470|China|Ruili
2015|KX056466|China|Ruili
1H4-CN21
2017|MG696654|Myanmar
2019|MN923081|China|Guangzhou
2018|MN955625|Thailand
2018|MN955656|Thailand
2018|MN955640|Thailand
2018|MN955653|Thailand
2018|MN955657|Thailand
2018|MN955643|Thailand
1H4-CN25
2015|KX357967|Myanmar
2015|KX357938|Myanmar
2018|MN933716|China|Guangzhou
1H4-CN22
2015|KX357940|Myanmar
2015|KX357917|Myanmar
2015|KX357946|Myanmar
2015|KX357935|Myanmar
2015|KX357954|Myanmar
2015|KX357963|Myanmar
2015|KX357930|Myanmar
2015|KY495792|Thailand
2017|MH729967|Myanmar
2018|MN955637|Thailand
2017|MG679801|Myanmar
2017|LC410183|Thailand
2018|MN955652|Thailand
1H4-CN23
2018|MN955631|Thailand
2018|MK529755|China|Jinghong
2018|MK529743|China|Jinghong
2018|MN955630|Thailand
1H4-CN24
China cluster
Country/region
China
Indonesia
Malaysia
Singapore
Myanmar
Thailand
Cambodia
Laos
Vietnam
India
Pakistan
Bhutan
Sri Lanka
Bangladesh
Others
1995
1996
1997
1998
1999
2000
2001
2002
2003
2004
2005
2006
2007
2008
2009
2010
2011
2012
2013
2014
2015
2016
2017
2018
2019
2020
2021

c 1J7

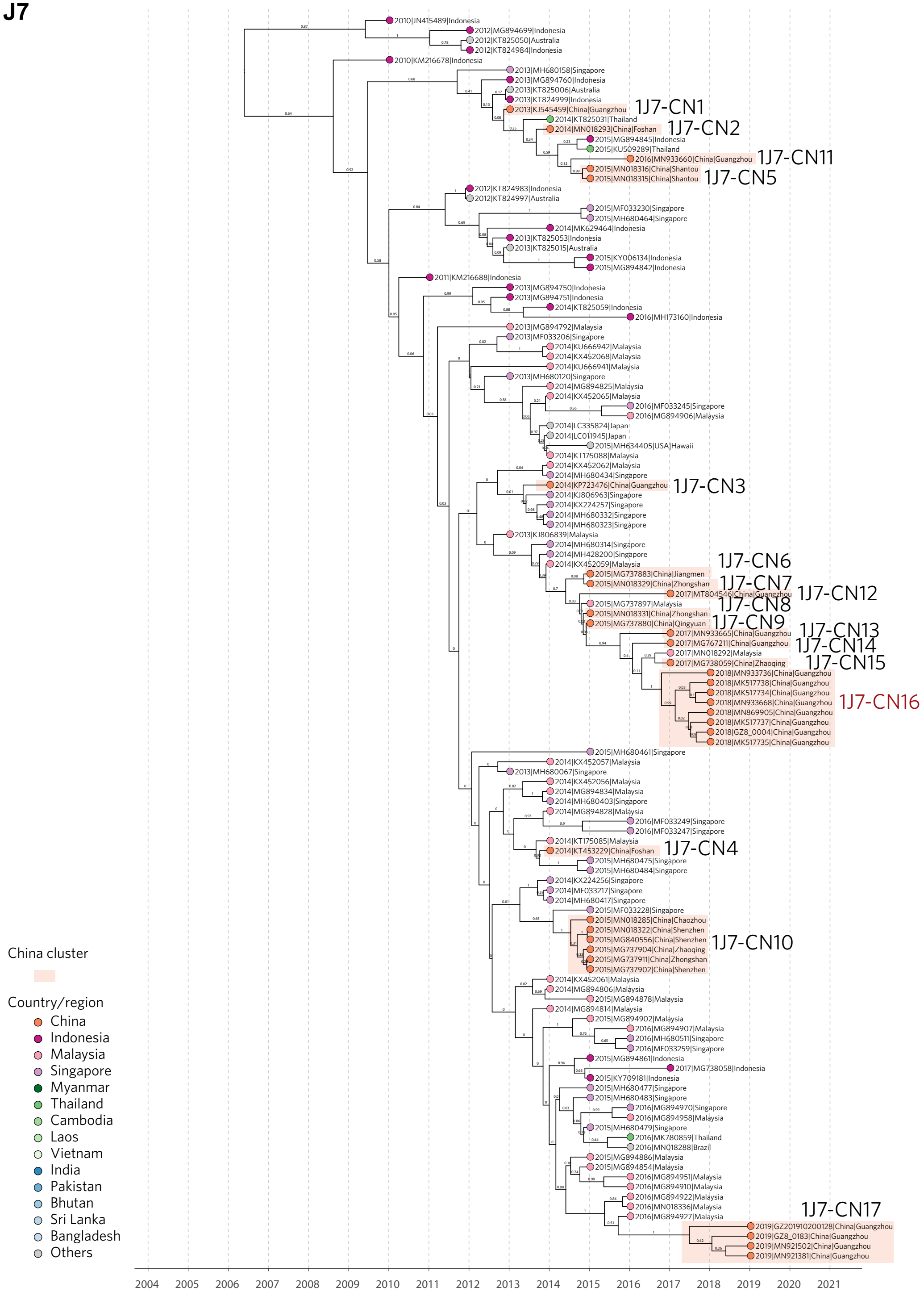
2010|JN415489|Indonesia
2012|MG894699|Indonesia
2012|KT825050|Australia
2012|KT824984|Indonesia
2010|KM216678|Indonesia
2013|MH680158|Singapore
2013|MG894760|Indonesia
2013|KT825006|Australia
2013|KT824999|Indonesia
2013|KJ545459|China|Guangzhou
1J7-CN1
2014|KT825031|Thailand
2014|MN018293|China|Foshan
1J7-CN2
2015|MG894845|Indonesia
2015|KU509289|Thailand
2016|MN933660|China|Guangzhou
1J7-CN11
2015|MN018316|China|Shantou
2015|MN018315|China|Shantou
1J7-CN5
2012|KT824983|Indonesia
2012|KT824997|Australia
2015|MF033230|Singapore
2015|MH680464|Singapore
2014|MK629464|Indonesia
2013|KT825053|Indonesia
2013|KT825015|Australia
2015|KY006134|Indonesia
2015|MG894842|Indonesia
2011|KM216688|Indonesia
2013|MG894750|Indonesia
2013|MG894751|Indonesia
2014|KT825059|Indonesia
2016|MH173160|Indonesia
2013|MG894792|Malaysia
2013|MF033206|Singapore
2014|KU666942|Malaysia
2014|KX452068|Malaysia
2014|KU666941|Malaysia
2013|MH680120|Singapore
2014|MG894825|Malaysia
2014|KX452065|Malaysia
2016|MF033245|Singapore
2016|MG894906|Malaysia
2014|LC335824|Japan
2014|LC011945|Japan
2015|MH634405|USA|Hawaii
2014|KT175088|Malaysia
2014|KX452062|Malaysia
2014|MH680434|Singapore
2014|KP723476|China|Guangzhou
1J7-CN3
2014|KJ806963|Singapore
2014|KX224257|Singapore
2014|MH680332|Singapore
2014|MH680323|Singapore
2013|KJ806839|Malaysia
2014|MH680314|Singapore
2014|MH428200|Singapore
2014|KX452059|Malaysia
1J7-CN6
2015|MG737883|China|Jiangmen
2015|MN018329|China|Zhongshan
1J7-CN7
2017|MT804546|China|Guangzhou
1J7-CN12
1J7-CN8
2015|MG737897|Malaysia
2015|MN018331|China|Zhongshan
2015|MG737880|China|Qingyuan
1J7-CN9
2017|MN933665|China|Guangzhou
1J7-CN13
2017|MG767211|China|Guangzhou
1J7-CN14
2017|MN018292|Malaysia
2017|MG738059|China|Zhaoqing
1J7-CN15
2018|MN933736|China|Guangzhou
2018|MK517738|China|Guangzhou
2018|MK517734|China|Guangzhou
2018|MN933668|China|Guangzhou
2018|MN869905|China|Guangzhou
2018|MK517737|China|Guangzhou
2018|GZ8_0004|China|Guangzhou
2018|MK517735|China|Guangzhou
1J7-CN16
2015|MH680461|Singapore
2014|KX452057|Malaysia
2013|MH680067|Singapore
2014|KX452056|Malaysia
2014|MG894834|Malaysia
2014|MH680403|Singapore
2014|MG894828|Malaysia
2016|MF033249|Singapore
2016|MF033247|Singapore
2014|KT175085|Malaysia
2014|KT453229|China|Foshan
1J7-CN4
2015|MH680475|Singapore
2015|MH680484|Singapore
2014|KX224256|Singapore
2014|MF033217|Singapore
2014|MH680417|Singapore
2015|MF033228|Singapore
2015|MN018285|China|Chaozhou
2015|MN018322|China|Shenzhen
2015|MG840556|China|Shenzhen
2015|MG737904|China|Zhaoqing
2015|MG737911|China|Zhongshan
2015|MG737902|China|Shenzhen
1J7-CN10
2014|KX452061|Malaysia
2014|MG894806|Malaysia
2015|MG894878|Malaysia
2014|MG894814|Malaysia
2015|MG894902|Malaysia
2016|MG894907|Malaysia
2016|MH680511|Singapore
2016|MF033259|Singapore
2015|MG894861|Indonesia
2017|MG738058|Indonesia
2015|KY709181|Indonesia
2015|MH680477|Singapore
2015|MH680483|Singapore
2016|MG894970|Singapore
2016|MG894958|Malaysia
2015|MH680479|Singapore
2016|MK780859|Thailand
2016|MN018288|Brazil
2015|MG894886|Malaysia
2015|MG894854|Malaysia
2016|MG894951|Malaysia
2016|MG894910|Malaysia
2016|MG894922|Malaysia
2016|MN018336|Malaysia
2016|MG894927|Malaysia
1J7-CN17
2019|GZ201910200128|China|Guangzhou
2019|GZ8_0183|China|Guangzhou
2019|MN921502|China|Guangzhou
2019|MN921381|China|Guangzhou
China cluster
Country/region
China
Indonesia
Malaysia
Singapore
Myanmar
Thailand
Cambodia
Laos
Vietnam
India
Pakistan
Bhutan
Sri Lanka
Bangladesh
Others
2004
2005
2006
2007
2008
2009
2010
2011
2012
2013
2014
2015
2016
2017
2018
2019
2020
2021

d 1K1

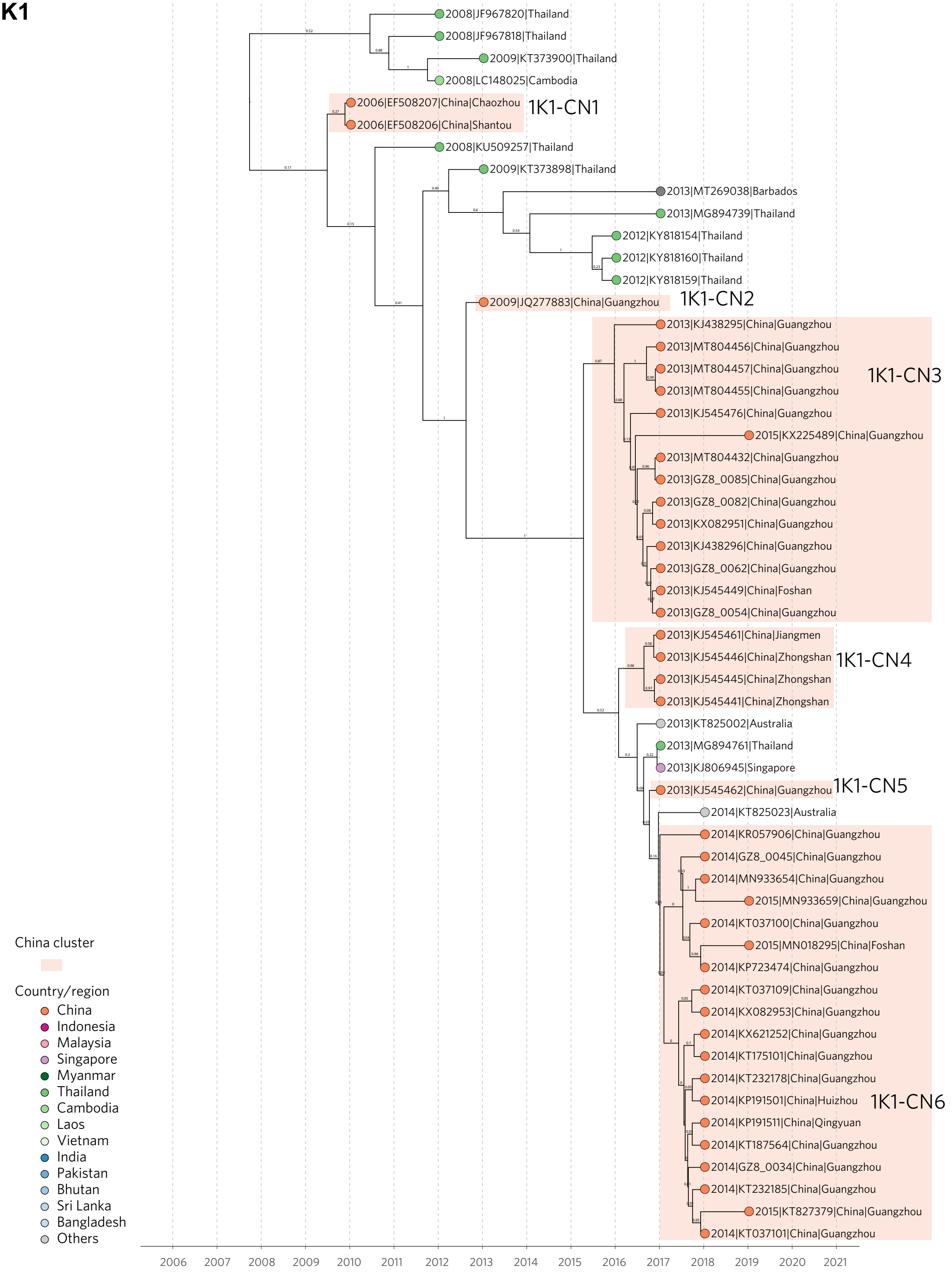
2008|JF967820|Thailand
2008|JF967818|Thailand
2009|KT373900|Thailand
2008|LC148025|Cambodia
2006|EF508207|China|Chaozhou
2006|EF508206|China|Shantou
1K1-CN1
2008|KU509257|Thailand
2009|KT373898|Thailand
2013|MT269038|Barbados
2013|MG894739|Thailand
2012|KY818154|Thailand
2012|KY818160|Thailand
2012|KY818159|Thailand
2009|JQ277883|China|Guangzhou
1K1-CN2
2013|KJ438295|China|Guangzhou
2013|MT804456|China|Guangzhou
2013|MT804457|China|Guangzhou
2013|MT804455|China|Guangzhou
1K1-CN3
2013|KJ545476|China|Guangzhou
2015|KX225489|China|Guangzhou
2013|MT804432|China|Guangzhou
2013|GZ8_0085|China|Guangzhou
2013|GZ8_0082|China|Guangzhou
2013|KX082951|China|Guangzhou
2013|KJ438296|China|Guangzhou
2013|GZ8_0062|China|Guangzhou
2013|KJ545449|China|Foshan
2013|GZ8_0054|China|Guangzhou
2013|KJ545461|China|Jiangmen
2013|KJ545446|China|Zhongshan
1K1-CN4
2013|KJ545445|China|Zhongshan
2013|KJ545441|China|Zhongshan
2013|KT825002|Australia
2013|MG894761|Thailand
2013|KJ806945|Singapore
2013|KJ545462|China|Guangzhou
1K1-CN5
2014|KT825023|Australia
2014|KR057906|China|Guangzhou
2014|GZ8_0045|China|Guangzhou
2014|MN933654|China|Guangzhou
2015|MN933659|China|Guangzhou
2014|KT037100|China|Guangzhou
2015|MN018295|China|Foshan
2014|KP723474|China|Guangzhou
2014|KT037109|China|Guangzhou
2014|KX082953|China|Guangzhou
2014|KX621252|China|Guangzhou
2014|KT175101|China|Guangzhou
2014|KT232178|China|Guangzhou
2014|KP191501|China|Huizhou
1K1-CN6
2014|KP191511|China|Qingyuan
2014|KT187564|China|Guangzhou
2014|GZ8_0034|China|Guangzhou
2014|KT232185|China|Guangzhou
2015|KT827379|China|Guangzhou
2014|KT037101|China|Guangzhou
China cluster
Country/region
China
Indonesia
Malaysia
Singapore
Myanmar
Thailand
Cambodia
Laos
Vietnam
India
Pakistan
Bhutan
Sri Lanka
Bangladesh
Others
2006
2007
2008
2009
2010
2011
2012
2013
2014
2015
2016
2017
2018
2019
2020
2021

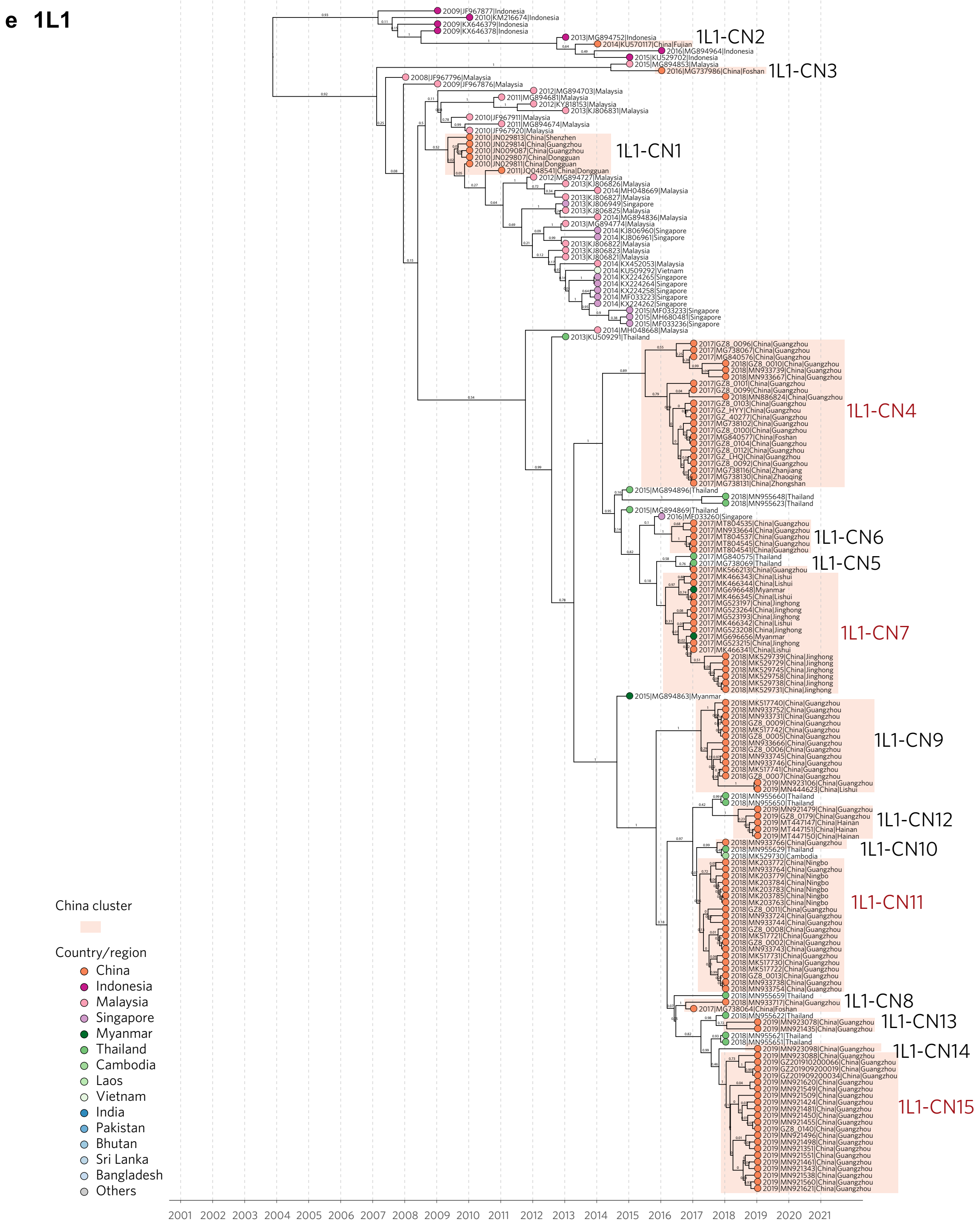
e 1L1
2009|JF967877|Indonesia
2010|KM216674|Indonesia
2009|KX646379|Indonesia
2009|KX646378|Indonesia
2013|MG894752|Indonesia
2014|KU570117|China|Fujian
1L1-CN2
2016|MG894964|Indonesia
2015|KU529702|Indonesia
2015|MG894853|Malaysia
2016|MG737986|China|Foshan
1L1-CN3
2008|JF967796|Malaysia
2009|JF967876|Malaysia
2012|MG894703|Malaysia
2011|MG894681|Malaysia
2012|KY818153|Malaysia
2013|KJ806831|Malaysia
2010|JF967911|Malaysia
2011|MG894674|Malaysia
2010|JF967920|Malaysia
2010|JN029813|China|Shenzhen
2010|JN029814|China|Guangzhou
2010|JN009087|China|Guangzhou
2010|JN029807|China|Dongguan
2010|JN029811|China|Dongguan
2011|JQ048541|China|Dongguan
1L1-CN1
2012|MG894727|Malaysia
2013|KJ806826|Malaysia
2014|MH048669|Malaysia
2013|KJ806827|Malaysia
2013|KJ806949|Singapore
2013|KJ806825|Malaysia
2014|MG894836|Malaysia
2013|MG894774|Malaysia
2014|KJ806960|Singapore
2014|KJ806961|Singapore
2013|KJ806822|Malaysia
2013|KJ806823|Malaysia
2013|KJ806821|Malaysia
2014|KX452053|Malaysia
2014|KU509292|Vietnam
2014|KX224265|Singapore
2014|KX224264|Singapore
2014|KX224258|Singapore
2014|MF033223|Singapore
2014|KX224262|Singapore
2015|MF033233|Singapore
2015|MH680481|Singapore
2015|MF033236|Singapore
2014|MH048668|Malaysia
2013|KU509291|Thailand
2017|GZ8_0096|China|Guangzhou
2017|MG738067|China|Guangzhou
2017|MG840576|China|Guangzhou
2018|GZ8_0010|China|Guangzhou
2018|MN933739|China|Guangzhou
2018|MN933667|China|Guangzhou
2017|GZ8_0101|China|Guangzhou
2017|GZ8_0099|China|Guangzhou
2018|MN886824|China|Guangzhou
2017|GZ8_0103|China|Guangzhou
2017|GZ_HYY|China|Guangzhou
2017|GZ_40277|China|Guangzhou
2017|MG738102|China|Guangzhou
2017|GZ8_0100|China|Guangzhou
2017|MG840577|China|Foshan
2017|GZ8_0104|China|Guangzhou
2017|GZ8_0112|China|Guangzhou
2017|GZ_LHQ|China|Guangzhou
2017|GZ8_0092|China|Guangzhou
2017|MG738116|China|Zhanjiang
2017|MG738130|China|Zhaoqing
2017|MG738131|China|Zhongshan
1L1-CN4
2015|MG894896|Thailand
2018|MN955648|Thailand
2018|MN955623|Thailand
2015|MG894869|Thailand
2016|MF033260|Singapore
2017|MT804535|China|Guangzhou
2017|MN933664|China|Guangzhou
2017|MT804537|China|Guangzhou
2017|MT804545|China|Guangzhou
2017|MT804541|China|Guangzhou
1L1-CN6
2017|MG840575|Thailand
2017|MG738069|Thailand
1L1-CN5
2017|MK566213|China|Guangzhou
2017|MK466343|China|Lishui
2017|MK466344|China|Lishui
2017|MG696648|Myanmar
2017|MK466345|China|Lishui
2017|MG523197|China|Jinghong
2017|MG523264|China|Jinghong
2017|MG523193|China|Jinghong
2017|MK466342|China|Lishui
2017|MG523208|China|Jinghong
2017|MG696656|Myanmar
2017|MG523215|China|Jinghong
2017|MK466341|China|Lishui
2018|MK529739|China|Jinghong
2018|MK529729|China|Jinghong
2018|MK529745|China|Jinghong
2018|MK529758|China|Jinghong
2018|MK529738|China|Jinghong
2018|MK529731|China|Jinghong
1L1-CN7
2015|MG894863|Myanmar
2018|MK517740|China|Guangzhou
2018|MN933752|China|Guangzhou
2018|MN933731|China|Guangzhou
2018|GZ8_0009|China|Guangzhou
2018|MK517742|China|Guangzhou
2018|GZ8_0005|China|Guangzhou
2018|MN933666|China|Guangzhou
2018|GZ8_0006|China|Guangzhou
2018|MN933745|China|Guangzhou
2018|MN933746|China|Guangzhou
2018|MK517741|China|Guangzhou
2018|GZ8_0007|China|Guangzhou
2019|MN923106|China|Guangzhou
2019|MN444623|China|Lishui
1L1-CN9
2018|MN955660|Thailand
2018|MN955650|Thailand
2019|MN921479|China|Guangzhou
2019|GZ8_0179|China|Guangzhou
2019|MT447147|China|Hainan
2019|MT447151|China|Hainan
2019|MT447150|China|Hainan
1L1-CN12
2018|MN933766|China|Guangzhou
2018|MN955629|Thailand
1L1-CN10
2018|MK529730|Cambodia
2018|MK203772|China|Ningbo
2018|MN933764|China|Guangzhou
2018|MK203779|China|Ningbo
2018|MK203784|China|Ningbo
2018|MK203783|China|Ningbo
2018|MK203785|China|Ningbo
2018|MK203763|China|Ningbo
2018|GZ8_0011|China|Guangzhou
2018|MN933724|China|Guangzhou
2018|MN933744|China|Guangzhou
2018|GZ8_0008|China|Guangzhou
2018|MK517721|China|Guangzhou
2018|GZ8_0002|China|Guangzhou
2018|MN933743|China|Guangzhou
2018|MK517731|China|Guangzhou
2018|MK517730|China|Guangzhou
2018|MK517722|China|Guangzhou
2018|GZ8_0013|China|Guangzhou
2018|MN933738|China|Guangzhou
2018|MN933754|China|Guangzhou
1L1-CN11
2018|MN955659|Thailand
2018|MN933717|China|Guangzhou
2017|MG738064|China|Foshan
1L1-CN8
2018|MN955622|Thailand
2019|MN923078|China|Guangzhou
2019|MN921435|China|Guangzhou
1L1-CN13
2018|MN955621|Thailand
2018|MN955651|Thailand
2019|MN923098|China|Guangzhou
2019|MN923088|China|Guangzhou
1L1-CN14
2019|GZ201910200066|China|Guangzhou
2019|GZ201909200019|China|Guangzhou
2019|GZ201909200034|China|Guangzhou
2019|MN921620|China|Guangzhou
2019|MN921549|China|Guangzhou
2019|MN921509|China|Guangzhou
2019|MN921424|China|Guangzhou
2019|MN921481|China|Guangzhou
2019|MN921450|China|Guangzhou
2019|MN921455|China|Guangzhou
2019|GZ8_0140|China|Guangzhou
2019|MN921496|China|Guangzhou
2019|MN921498|China|Guangzhou
2019|MN921351|China|Guangzhou
2019|MN921551|China|Guangzhou
2019|MN921461|China|Guangzhou
2019|MN921343|China|Guangzhou
2019|MN921538|China|Guangzhou
2019|MN921560|China|Guangzhou
2019|MN921621|China|Guangzhou
1L1-CN15
China cluster
Country/region
China
Indonesia
Malaysia
Singapore
Myanmar
Thailand
Cambodia
Laos
Vietnam
India
Pakistan
Bhutan
Sri Lanka
Bangladesh
Others
2001
2002
2003
2004
2005
2006
2007
2008
2009
2010
2011
2012
2013
2014
2015
2016
2017
2018
2019
2020
2021

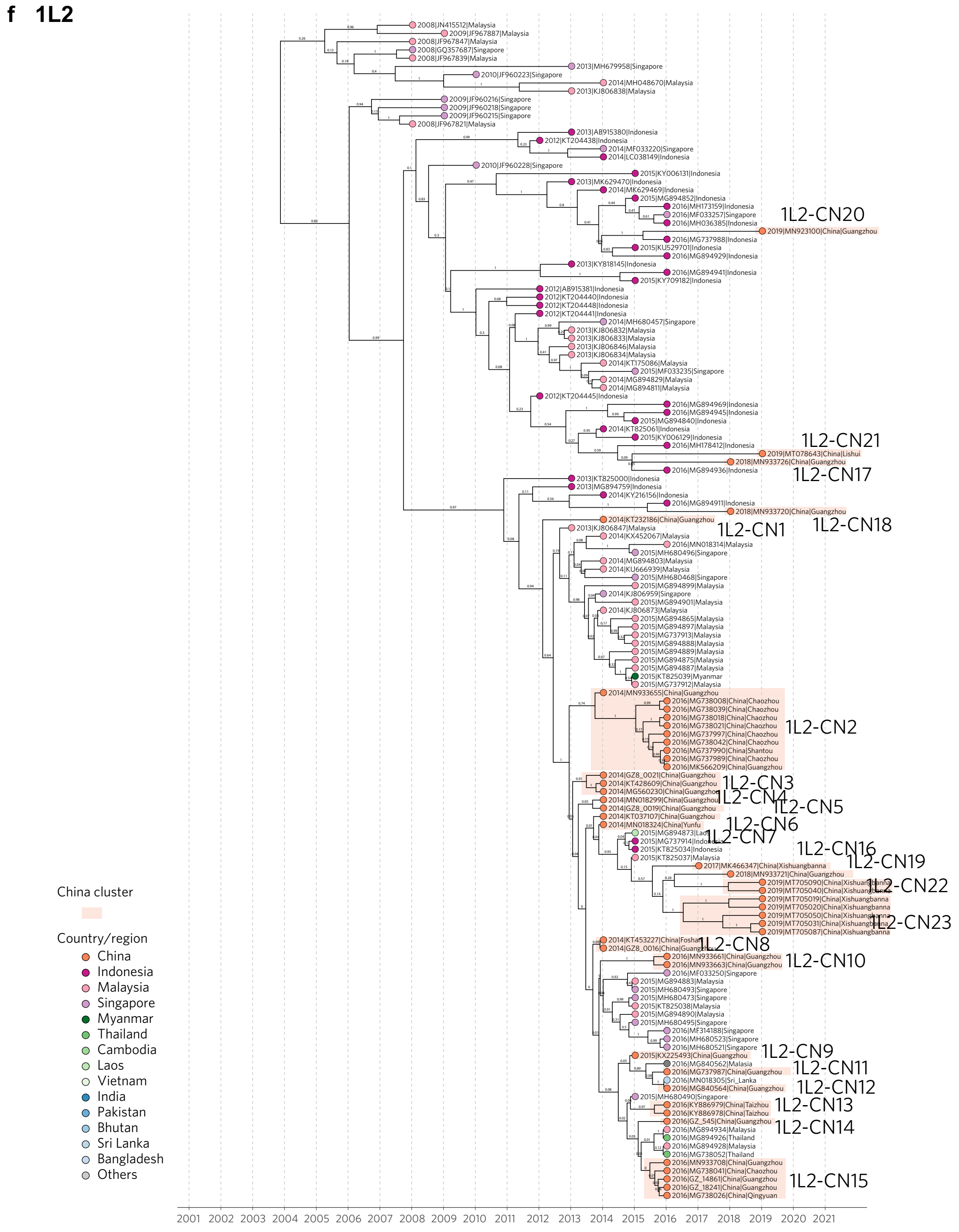
f 1L2
2008|JN415512|Malaysia
2009|JF967887|Malaysia
2008|JF967847|Malaysia
2008|GQ357687|Singapore
2008|JF967839|Malaysia
2013|MH679958|Singapore
2010|JF960223|Singapore
2014|MH048670|Malaysia
2013|KJ806838|Malaysia
2009|JF960216|Singapore
2009|JF960218|Singapore
2009|JF960215|Singapore
2008|JF967821|Malaysia
2013|AB915380|Indonesia
2012|KT204438|Indonesia
2014|MF033220|Singapore
2014|LC038149|Indonesia
2010|JF960228|Singapore
2015|KY006131|Indonesia
2013|MK629470|Indonesia
2014|MK629469|Indonesia
2015|MG894852|Indonesia
2016|MH173159|Indonesia
2016|MF033257|Singapore
2016|MH036385|Indonesia
1L2-CN20
2019|MN923100|China|Guangzhou
2016|MG737988|Indonesia
2015|KU529701|Indonesia
2016|MG894929|Indonesia
2013|KY818145|Indonesia
2016|MG894941|Indonesia
2015|KY709182|Indonesia
2012|AB915381|Indonesia
2012|KT204440|Indonesia
2012|KT204448|Indonesia
2012|KT204441|Indonesia
2014|MH680457|Singapore
2013|KJ806832|Malaysia
2013|KJ806833|Malaysia
2013|KJ806846|Malaysia
2013|KJ806834|Malaysia
2014|KT175086|Malaysia
2015|MF033235|Singapore
2014|MG894829|Malaysia
2014|MG894811|Malaysia
2012|KT204445|Indonesia
2016|MG894969|Indonesia
2016|MG894945|Indonesia
2015|MG894840|Indonesia
2014|KT825061|Indonesia
2015|KY006129|Indonesia
2016|MH178412|Indonesia
1L2-CN21
2019|MT078643|China|Lishui
2018|MN933726|China|Guangzhou
2016|MG894936|Indonesia
1L2-CN17
2013|KT825000|Indonesia
2013|MG894759|Indonesia
2014|KY216156|Indonesia
2016|MG894911|Indonesia
2018|MN933720|China|Guangzhou
1L2-CN18
2014|KT232186|China|Guangzhou
1L2-CN1
2013|KJ806847|Malaysia
2014|KX452067|Malaysia
2016|MN018314|Malaysia
2015|MH680496|Singapore
2014|MG894803|Malaysia
2014|KU666939|Malaysia
2015|MH680468|Singapore
2015|MG894899|Malaysia
2014|KJ806959|Singapore
2015|MG894901|Malaysia
2014|KJ806873|Malaysia
2015|MG894865|Malaysia
2015|MG894897|Malaysia
2015|MG737913|Malaysia
2015|MG894888|Malaysia
2015|MG894889|Malaysia
2015|MG894875|Malaysia
2015|MG894887|Malaysia
2015|KT825039|Myanmar
2015|MG737912|Malaysia
2014|MN933655|China|Guangzhou
2016|MG738008|China|Chaozhou
2016|MG738039|China|Chaozhou
2016|MG738018|China|Chaozhou
2016|MG738021|China|Chaozhou
2016|MG737997|China|Chaozhou
2016|MG738042|China|Chaozhou
2016|MG737990|China|Shantou
2016|MG737989|China|Chaozhou
2016|MK566209|China|Guangzhou
1L2-CN2
2014|GZ8_0021|China|Guangzhou
2014|KT428609|China|Guangzhou
2014|MG560230|China|Guangzhou
1L2-CN3
2014|MN018299|China|Guangzhou
1L2-CN4
2014|GZ8_0019|China|Guangzhou
1L2-CN5
2014|KT037107|China|Guangzhou
2014|MN018324|China|Yunfu
1L2-CN6
2015|MG894873|Laos
2015|MG737914|Indonesia
1L2-CN7
2015|KT825034|Indonesia
2015|KT825037|Malaysia
1L2-CN16
2017|MK466347|China|Xishuangbanna
1L2-CN19
2018|MN933721|China|Guangzhou
2019|MT705090|China|Xishuangbanna
2019|MT705040|China|Xishuangbanna
1L2-CN22
2019|MT705019|China|Xishuangbanna
2019|MT705020|China|Xishuangbanna
2019|MT705050|China|Xishuangbanna
2019|MT705031|China|Xishuangbanna
2019|MT705087|China|Xishuangbanna
1L2-CN23
2014|KT453227|China|Foshan
2014|GZ8_0016|China|Guangzhou
1L2-CN8
2016|MN933661|China|Guangzhou
2016|MN933663|China|Guangzhou
1L2-CN10
2016|MF033250|Singapore
2015|MG894883|Malaysia
2015|MH680493|Singapore
2015|MH680473|Singapore
2015|KT825038|Malaysia
2015|MG894890|Malaysia
2015|MH680495|Singapore
2016|MF314188|Singapore
2016|MH680523|Singapore
2016|MH680521|Singapore
2015|KX225493|China|Guangzhou
1L2-CN9
2016|MG840562|Malasia
2016|MG737987|China|Guangzhou
1L2-CN11
2016|MN018305|Sri_Lanka
2016|MG840564|China|Guangzhou
1L2-CN12
2015|MH680490|Singapore
2016|KY886979|China|Taizhou
2016|KY886978|China|Taizhou
1L2-CN13
2016|GZ_545|China|Guangzhou
1L2-CN14
2016|MG894934|Malaysia
2016|MG894926|Thailand
2016|MG894928|Malaysia
2016|MG738052|Thailand
2016|MN933708|China|Guangzhou
2016|MG738041|China|Chaozhou
2016|GZ_14861|China|Guangzhou
2016|GZ_18241|China|Guangzhou
2016|MG738026|China|Qingyuan
1L2-CN15
China cluster
Country/region
China
Indonesia
Malaysia
Singapore
Myanmar
Thailand
Cambodia
Laos
Vietnam
India
Pakistan
Bhutan
Sri Lanka
Bangladesh
Others
2001 2002 2003 2004 2005 2006 2007 2008 2009 2010 2011 2012 2013 2014 2015 2016 2017 2018 2019 2020 2021

g 5C1

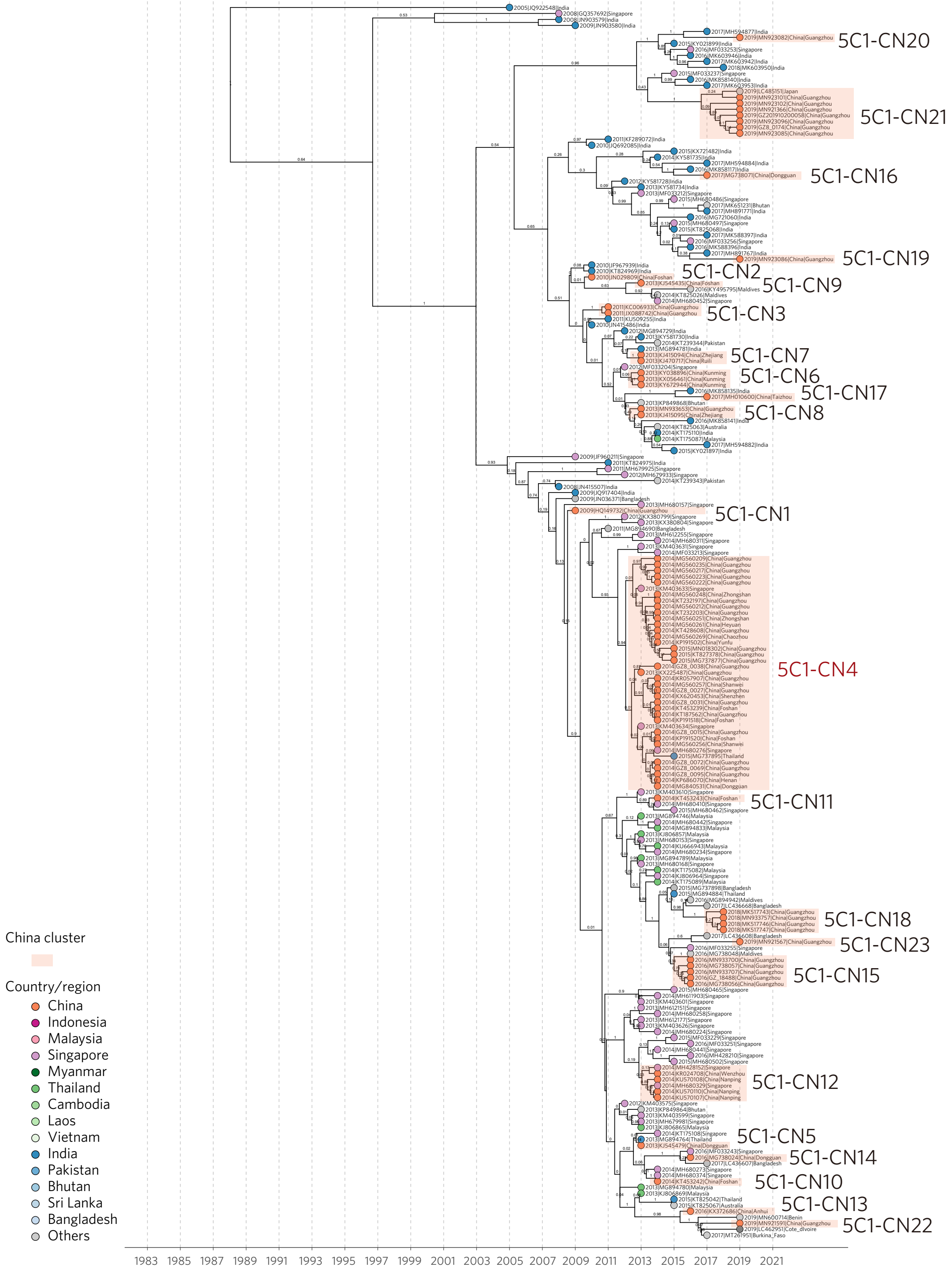
5C1-CN20
5C1-CN21
5C1-CN16
5C1-CN19
5C1-CN2
5C1-CN9
5C1-CN3
5C1-CN7
5C1-CN6
5C1-CN17
5C1-CN8
5C1-CN1
5C1-CN4
5C1-CN11
5C1-CN18
5C1-CN23
5C1-CN15
5C1-CN12
5C1-CN5
5C1-CN14
5C1-CN10
5C1-CN13
5C1-CN22
China cluster
Country/region
China
Indonesia
Malaysia
Singapore
Myanmar
Thailand
Cambodia
Laos
Vietnam
India
Pakistan
Bhutan
Sri Lanka
Bangladesh
Others
1983
1985
1987
1989
1991
1993
1995
1997
1999
2001
2003
2005
2007
2009
2011
2013
2015
2017
2019
2021
