# Supplementary material for: Phylodynamics unveils invading and diffusing patterns of dengue virus serotype-1 in Guangdong, China from 1990 to 2019 under a global genotyping framework
Source: Infect Dis Poverty. 2024 Jun 11;13:43. doi: 10.1186/s40249-024-01211-6 (PMC11165891; doi:10.1186/s40249-024-01211-6)
Supplement: Supplementary file 11 — Additional file 11: Table S5. The introduction and diffusion routs of the seven COCs in Chinese mainland inferred by BSSVS. [file 40249_2024_1211_MOESM11_ESM.pdf]

Table S5. The introduction and diffusion transmission route in mainland China of seven Clades of Concern (CoCs) inferred by BSSVS.

No.	Clade	From	To	Bayes factor	Posterior probability	Migration rate
1	1E1	Cambodia	Guangzhou	7.6506	0.2737	0.8314
2	1E1	Cambodia	Vietnam	10.8422	0.3482	0.9127
3	1E1	Cambodia	Yangjiang	20.9524	0.5079	0.8973
4	1E1	French Polynesia	Hawaii	35.7784	0.6380	0.9589
5	1E1	Guangzhou	Jieyang	42.4843	0.6767	1.1175
6	1E1	Guangzhou	Lishui	15.0266	0.4254	0.6532
7	1E1	Guangzhou	Myanmar	6.2727	0.2361	0.7843
8	1E1	Guangzhou	Puyang	7922.9086	0.9974	1.0884
9	1E1	Guangzhou	Shenzhen	108.5420	0.8425	0.6588
10	1E1	Guangzhou	Xishuangbanna	182673.4333	1.0000	1.6977
11	1E1	Guangzhou	Yuzhou	125.1597	0.8605	0.6462
12	1E1	Guangzhou	Zhanjiang	18.4339	0.4759	0.8055
13	1E1	Jieyang	Zhanjiang	11.6480	0.3646	0.9859
14	1E1	Singapore	Taizhou	32.1559	0.6130	1.4451
15	1E1	Vietnam	Zhanjiang	8.7388	0.3010	0.6177
16	1E1	Vietnam	Cambodia	38.5794	0.6553	0.5429
17	1E1	Yangjiang	Cambodia	11.3492	0.3586	0.9179
18	1E1	French Polynesia	Cook Islands	8.0100	0.2830	0.7566
19	1E1	New Caledonia	Cook Islands	37.1719	0.6468	1.0913
20	1E1	Vietnam	Foshan	41.0507	0.6691	0.3819
21	1E1	New Caledonia	French Polynesia	131.3160	0.8661	1.2624
22	1E1	Vietnam	Fuzhou	197.9750	0.9070	0.5187
23	1E1	Vietnam	Guangzhou	222.6467	0.9165	1.1502
24	1E1	New Caledonia	Hawaii	9.5451	0.3199	0.8291
25	1E1	Vietnam	Jieyang	10.6732	0.3446	0.7623
26	1E1	Xishuangbanna	Lishui	20.6657	0.5045	0.8617
27	1E1	Vietnam	Longyan	9.3178	0.3146	0.4950
28	1E1	Xishuangbanna	Myanmar	91.1016	0.8178	1.7916
29	1E1	Singapore	Nanning	24.9130	0.5510	0.9325
30	1E1	Vietnam	Nanning	10.0054	0.3302	0.5123
31	1E1	Vietnam	New Caledonia	17.7404	0.4664	0.4719
32	1E1	Vietnam	Singapore	182673.4333	0.9999	1.2914
33	1E1	Vietnam	Taizhou	24.3386	0.5453	0.6786
34	1E1	Yangjiang	Vietnam	26.7890	0.5689	1.0114
35	1H4	Gengma	Lincang	93.0688	0.9005	0.9339
36	1H4	Guangzhou	Thailand	128.7628	0.9260	0.8760
37	1H4	Kunming	Thailand	6.5707	0.3897	0.8058
38	1H4	Myanmar	Ruili	210.2072	0.9533	2.0225
39	1H4	Myanmar	Singapore	109.8258	0.9143	0.8723
40	1H4	Myanmar	Thailand	60.9484	0.8556	1.1968
41	1H4	Guangzhou	Foshan	651.1989	0.9844	0.7917
42	1H4	Malaysia	Fuzhou	9.4278	0.4782	0.7996

43	1H4	Myanmar	Gengma	11.3286	0.5241	0.5354
44	1H4	Myanmar	Guangzhou	15.7835	0.6054	0.7999
45	1H4	Ruili	Guangzhou	8.8334	0.4619	0.8742
46	1H4	Thailand	Jinghong	112.2090	0.9160	0.6982
47	1H4	Thailand	Kunming	15.8645	0.6066	0.6897
48	1H4	Thailand	Malaysia	10.3874	0.5024	0.6579
49	1H4	Ruili	Myanmar	9.4530	0.4788	1.0572
50	1H4	Thailand	Myanmar	134.8653	0.9291	2.3367
51	1J7	Guangzhou	Zhaoqing	15.7949	0.5080	0.7833
52	1J7	Indonesia	Malaysia	44.7156	0.7451	0.8835
53	1J7	Indonesia	Singapore	9.8084	0.3907	0.8130
54	1J7	Indonesia	Thailand	12.6463	0.4526	0.7016
55	1J7	Malaysia	Singapore	71.3950	0.8236	3.1065
56	1J7	Malaysia	Zhaoqing	7.9048	0.3407	0.7020
57	1J7	Malaysia	Zhongshan	16.4107	0.5176	0.6683
58	1J7	Shenzhen	Zhaoqing	7.1520	0.3186	0.8032
59	1J7	Shenzhen	Zhongshan	6.2358	0.2896	0.7704
60	1J7	Indonesia	Australia	137647.0027	0.9999	1.4583
61	1J7	Singapore	Brazil	10.6212	0.4098	0.5994
62	1J7	Shenzhen	Chaozhou	9.3192	0.3786	0.7558
63	1J7	Zhaoqing	Chaozhou	6.0521	0.2835	0.7600
64	1J7	Zhongshan	Chaozhou	7.2034	0.3202	0.7646
65	1J7	Indonesia	Foshan	10.0115	0.3956	0.7022
66	1J7	Singapore	Foshan	6.3509	0.2934	0.7137
67	1J7	Indonesia	Guangzhou	8.0980	0.3462	0.7060
68	1J7	Malaysia	Guangzhou	229.2215	0.9375	1.3940
69	1J7	Japan	Hawaii	41.7325	0.7318	1.0183
70	1J7	Malaysia	Japan	46.1348	0.7510	0.4770
71	1J7	Zhongshan	Jiangmen	10.6652	0.4108	0.8056
72	1J7	Zhongshan	Qingyuan	12.7602	0.4548	0.7916
73	1J7	Thailand	Shantou	11.3795	0.4266	0.7876
74	1J7	Zhaoqing	Shenzhen	8.4408	0.3556	0.8128
75	1J7	Zhongshan	Shenzhen	9.3589	0.3796	0.8262
76	1J7	Zhongshan	Zhaoqing	6.6581	0.3033	0.8012
77	1K1	Australia	Singapore	7.2203	0.3901	0.8574
78	1K1	Chaozhou	Shantou	9.3766	0.4631	0.8972
79	1K1	Chaozhou	Thailand	8.8451	0.4393	0.9712
80	1K1	Guangzhou	Huizhou	38.7454	0.7744	0.6324
81	1K1	Guangzhou	Qingyuan	36.7809	0.7651	0.6408
82	1K1	Guangzhou	Zhongshan	8.8850	0.4404	0.6495
83	1K1	Shantou	Thailand	11.0542	0.4947	1.0127
84	1K1	Guangzhou	Australia	8.6046	0.4325	0.6819
85	1K1	Thailand	Barbados	15.6868	0.5815	0.7011
86	1K1	Thailand	Cambodia	93,2594	0.8920	0.8017
87	1K1	Shantou	Chaozhou	13.0156	0.5355	0.9597

88	1K1	Guangzhou	Foshan	725.1023	0.9847	0.9909
89	1K1	Thailand	Guangzhou	13.0797	0.5367	0.7623
90	1K1	Zhongshan	Jiangmen	56.5938	0.8337	1.1063
91	1L1	Dongguan	Guangzhou	15.5477	0.4734	0.8651
92	1L1	Dongguan	Shenzhen	55.5868	0.7627	0.9856
93	1L1	Guangzhou	Haikou	28.2107	0.6199	0.5478
94	1L1	Guangzhou	Lishui	181.0186	0.9128	0.6628
95	1L1	Guangzhou	Ningbo	87.6079	0.8351	0.4859
96	1L1	Guangzhou	Zhanjiang	20.0370	0.5367	0.5732
97	1L1	Guangzhou	Zhaoqing	22.7448	0.5680	0.5244
98	1L1	Guangzhou	Zhongshan	18.9590	0.5229	0.5520
99	1L1	Indonesia	Malaysia	13.7034	0.4421	0.7015
100	1L1	Jinghong	Myanmar	7.4701	0.3016	0.8684
101	1L1	Lishui	Myanmar	58.2390	0.7710	1.5497
102	1L1	Malaysia	Singapore	155659.2717	1.0000	1.6997
103	1L1	Malaysia	Thailand	40.6415	0.7015	0.7479
104	1L1	Singapore	Vietnam	94.5411	0.8454	0.9586
105	1L1	Thailand	Cambodia	100.1963	0.8528	0.6493
106	1L1	Malaysia	Dongguan	24.0309	0.5815	0.6189
107	1L1	Guangzhou	Foshan	199.2227	0.9201	0.7805
108	1L1	Malaysia	Foshan	16.1978	0.4836	0.6363
109	1L1	Indonesia	Fuzhou	724.0215	0.9767	0.7748
110	1L1	Thailand	Guangzhou	3159.7773	0.9946	4.6801
111	1L1	Thailand	Haikou	6.9381	0.2863	0.6495
112	1L1	Lishui	Jinghong	220.0163	0.9271	1.7373
113	1L1	Thailand	Lishui	6.7435	0.2805	0.7150
114	1L1	Thailand	Myanmar	27.8282	0.6167	0.6492
115	1L1	Thailand	Singapore	50.1846	0.7437	0.6856
116	1L2	Chaozhou	Guangzhou	8.6763	0.3620	0.7795
117	1L2	Chaozhou	Shantou	26.2957	0.6322	0.8875
118	1L2	Guangzhou	Malaysia	55.5563	0.7841	1.9011
119	1L2	Guangzhou	Qingyuan	9.4608	0.3822	0.6601
120	1L2	Guangzhou	Shantou	8.2701	0.3510	0.7115
121	1L2	Guangzhou	Sri Lanka	176.7035	0.9203	0.6988
122	1L2	Guangzhou	Taizhou	11.2151	0.4231	0.6994
123	1L2	Guangzhou	Thailand	12.8692	0.4569	0.7227
124	1L2	Guangzhou	Yunfu	39.2256	0.7195	0.6817
125	1L2	Indonesia	Lishui	11.2662	0.4242	0.7530
126	1L2	Indonesia	Malaysia	10.0487	0.3965	0.9240
127	1L2	Indonesia	Singapore	40.2597	0.7247	0.7837
128	1L2	Malaysia	Myanmar	113.6032	0.8813	0.5116
129	1L2	Malaysia	Singapore	137647.0027	1.0000	4.7733
130	1L2	Guangzhou	Chaozhou	280.7538	0.9483	1.2218
131	1L2	Guangzhou	Foshan	27.6582	0.6439	0.6665
132	1L2	Indonesia	Guangzhou	855.9862	0.9824	1.4607

133	1L2	Malaysia	Indonesia	18.4384	0.5466	0.7741
134	1L2	Singapore	Indonesia	11.2662	0.4242	0.9519
135	1L2	Malaysia	Laos	6.5883	0.3011	0.6027
136	1L2	Thailand	Qingyuan	6.3816	0.2944	0.7853
137	5C1	Wuhu	Burkina Faso	11.5323	0.2895	0.8301
138	5C1	Australia	Thailand	7.3163	0.2054	0.6981
139	5C1	Bangladesh	Guangzhou	13.6442	0.3253	1.1127
140	5C1	Bangladesh	Maldives	8.3937	0.2288	0.9053
141	5C1	Benin	Cote dIvorie	9.6964	0.2552	0.8623
142	5C1	Benin	Guangzhou	10.8587	0.2773	0.8488
143	5C1	Bhutan	Kunming	18.2595	0.3922	1.0672
144	5C1	Burkina Faso	Cote dIvoire	24.3295	0.4623	0.9087
145	5C1	Burkina Faso	Guangzhou	9.8215	0.2576	0.8880
146	5C1	Chaozhou	Heyuan	9.1214	0.2438	0.6595
147	5C1	Cote dIvoire	Guangzhou	8.5850	0.2328	0.8355
148	5C1	Dongguan	Shangqiu	6.7961	0.1936	0.6503
149	5C1	Foshan	Shanwei	6.3757	0.1839	0.8529
150	5C1	Foshan	Shenzhen	17.1545	0.3774	0.8210
151	5C1	Guangzhou	Shangqiu	8.4997	0.2310	0.6147
152	5C1	Guangzhou	Heyuan	24.9234	0.4683	0.5488
153	5C1	Guangzhou	Japan	37.5719	0.5704	0.5953
154	5C1	Guangzhou	Maldives	12.9314	0.3136	0.7992
155	5C1	Guangzhou	Shanwei	94.9944	0.7705	0.7833
156	5C1	Guangzhou	Shenzhen	13.5066	0.3231	0.7069
157	5C1	Guangzhou	Yunfu	89.0853	0.7589	0.5742
158	5C1	Guangzhou	Zhongshan	2493.7350	0.9888	0.8551
159	5C1	India	Kunming	11.3710	0.2866	0.5579
160	5C1	India	Pakistan	28.2181	0.4993	0.5831
161	5C1	India	Singapore	254697.2077	1.0000	3.1235
162	5C1	India	Taizhou	13.0989	0.3164	0.4966
163	5C1	India	Hangzhou	329.4609	0.9209	0.4960
164	5C1	Kunming	Taizhou	6.6133	0.1894	0.7745
165	5C1	Malaysia	Thailand	19.5991	0.4092	1.1893
166	5C1	Shanwei	Shenzhen	6.2394	0.1806	0.6577
167	5C1	Singapore	Thailand	54.0026	0.6561	0.9189
168	5C1	Singapore	Wenzhou	9.2760	0.2469	0.6552
169	5C1	Malaysia	Wuhu	13.5409	0.3236	0.7990
170	5C1	India	Australia	6.5465	0.1879	0.5923
171	5C1	Malaysia	Australia	12.8514	0.3123	1.0171
172	5C1	Thailand	Australia	50.0292	0.6387	1.0600
173	5C1	Singapore	Bangladesh	3004.1468	0.9907	1.2452
174	5C1	Burkina Faso	Benin	21.7250	0.4343	0.8303
175	5C1	Cote dIvoire	Benin	9.9704	0.2605	0.7283
176	5C1	India	Bhutan	9.9474	0.2601	0.6433
177	5C1	Singapore	Bhutan	75.2894	0.7268	0.7084

178	5C1	Malaysia	Burkina Faso	7.0053	0.1984	0.7584
179	5C1	Guangzhou	Chaozhou	29.9233	0.5139	0.5987
180	5C1	Singapore	Dongguan	3196.0738	0.9912	0.9877
181	5C1	Guangzhou	Foshan	22.2210	0.4398	0.7683
182	5C1	Singapore	Foshan	510.2320	0.9475	1.1384
183	5C1	India	Guangzhou	25.0571	0.4696	0.8389
184	5C1	Singapore	Guangzhou	289.7097	0.9110	2.0424
185	5C1	Taizhou	India	6.6468	0.1902	0.7205
186	5C1	Singapore	Kunming	6.6038	0.1892	0.6227
187	5C1	Singapore	Malaysia	3610.6361	0.9922	2.3362
188	5C1	Singapore	Maldives	40.4521	0.5884	0.7613
189	5C1	Singapore	Nanping	18.3192	0.3930	0.5312
190	5C1	Singapore	Pakistan	6.8494	0.1949	0.6566
191	5C1	Hangzhou	Pakistan	6.2628	0.1812	0.8423
192	5C1	Hangzhou	Ruili	255.0435	0.9001	1.0310
