# Supplementary material for: Phylodynamics unveils invading and diffusing patterns of dengue virus serotype-1 in Guangdong, China from 1990 to 2019 under a global genotyping framework
Source: Infect Dis Poverty. 2024 Jun 11;13:43. doi: 10.1186/s40249-024-01211-6 (PMC11165891; doi:10.1186/s40249-024-01211-6)
Supplement: Supplementary file 9 — Additional file 9: Figure S5. The cities were involved in the circulation of seven COCs transmission clusters in Chinese mainland from 1990 to 2019. Different cities are represented by color. [file 40249_2024_1211_MOESM9_ESM.pdf]

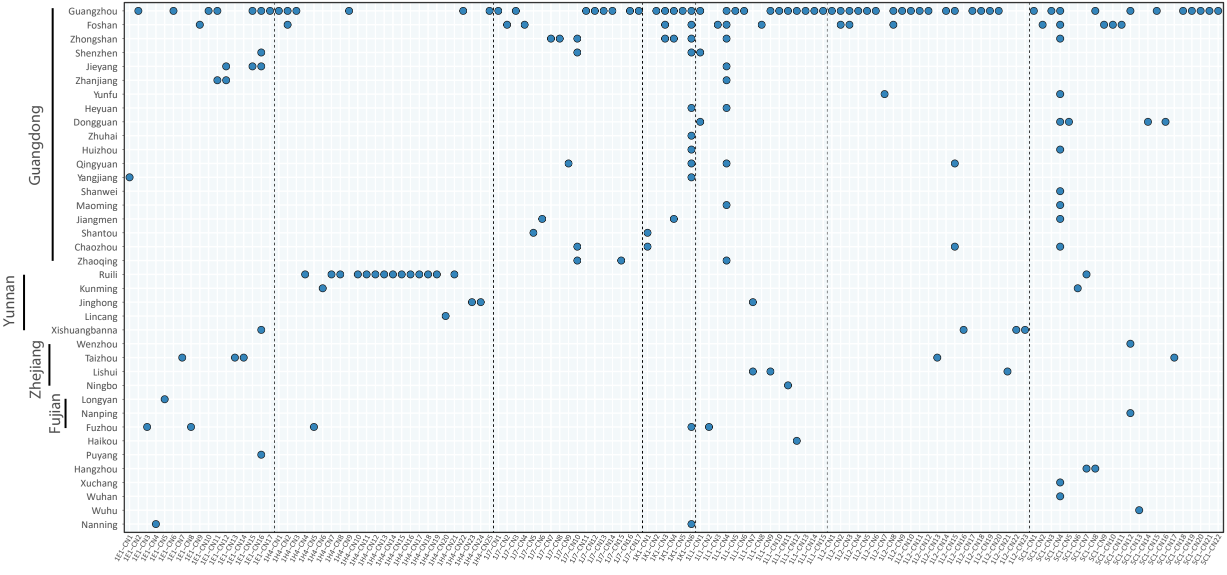
Guangdong
Yunnan
Zhejiang
Fujian
Guangzhou
Foshan
Zhongshan
Shenzhen
Jieyang
Zhanjiang
Yunfu
Heyuan
Dongguan
Zhuhai
Huizhou
Qingyuan
Yangjiang
Shanwei
Maoming
Jiangmen
Shantou
Chaozhou
Zhaoqing
Ruili
Kunming
Jinghong
Lincang
Xishuangbanna
Wenzhou
Taizhou
Lishui
Ningbo
Longyan
Nanping
Fuzhou
Haikou
Puyang
Hangzhou
Xuchang
Wuhan
Wuhu
Nanning
